# Effects of Synbiotics, Probiotics, and Prebiotics on Liver Enzymes of Patients With Non-alcoholic Fatty Liver Disease: A Systematic Review and Network Meta-Analysis

**DOI:** 10.3389/fnut.2022.880014

**Published:** 2022-05-20

**Authors:** Sukrit Kanchanasurakit, Chayanis Kositamongkol, Kamonnat Lanoi, Monnaree Nunta, Thaksaporn Saetuan, Nathorn Chaiyakunapruk, Surasak Saokaew, Pochamana Phisalprapa

**Affiliations:** ^1^Unit of Excellence on Clinical Outcomes Research and IntegratioN (UNICORN), School of Pharmaceutical Sciences, University of Phayao, Phayao, Thailand; ^2^Center of Health Outcomes Research and Therapeutic Safety (Cohorts), School of Pharmaceutical Sciences, University of Phayao, Phayao, Thailand; ^3^Unit of Excellence on Herbal Medicine, School of Pharmaceutical Sciences, University of Phayao, Phayao, Thailand; ^4^Division of Clinical Pharmacy, Department of Pharmaceutical Care, School of Pharmaceutical Sciences, University of Phayao, Phayao, Thailand; ^5^Division of Pharmaceutical Care, Department of Pharmacy, Phrae Hospital, Phrae, Thailand; ^6^Division of Ambulatory Medicine, Department of Medicine, Faculty of Medicine Siriraj Hospital, Mahidol University, Bangkok, Thailand; ^7^Department of Pharmacotherapy, College of Pharmacy, University of Utah, Salt Lake City, UT, United States; ^8^IDEAS Center, Veterans Affairs Salt Lake City Healthcare System, Salt Lake City, UT, United States; ^9^Division of Social and Administrative Pharmacy, Department of Pharmaceutical Care, School of Pharmaceutical Sciences, University of Phayao, Phayao, Thailand

**Keywords:** non-alcoholic fatty liver disease (NAFLD), non-alcoholic steatohepatitis (NASH), meta-analysis, synbiotic, probiotic, prebiotic, liver enzymes

## Abstract

**Background:**

A systematic review and network meta-analysis was primarily conducted to compare the effects of synbiotics, probiotics, and prebiotics on aspartate aminotransferase (AST) and alanine aminotransferase (ALT). Moreover, their effects on body mass index (BMI), waist circumference (WC), lipid profile, fasting blood sugar (FBS), and homeostatic model assessment-insulin resistance (HOMA-IR) of patients with non-alcoholic fatty liver disease (NAFLD) were investigated and analyzed as secondary outcomes.

**Methods:**

The randomized controlled trials (RCTs), limited to the English language, were searched through PubMed, the Web of Science, Embase, CLINAHL Plus, and the Cochrane Library from inception to February 2, 2022. The eligible studies were reviewed and their risk-of-bias and heterogeneity were assessed. Both direct and indirect evidence were assembled using a random-effects model. The effects of the intervention were presented as weighted mean differences (WMD) with 95% confidence interval (95% CI).

**Results:**

Of 3,864 identified records, a total of 1,389 patients with NAFLD from 26 RCTs were included in the analyses. Among these, 241 were diagnosed with non-alcoholic steatohepatitis. The quality assessment reported a moderate risk of bias from most studies. Among adult patients with NAFLD, when compared with placebo, synbiotics provided the largest effect on reductions of AST (−12.71 IU/L; 95% CI: −16.95, −8.47), WC (−2.26 cm; 95% CI: −2.98, −1.54), total cholesterol (−22.23 mg/dl; 95% CI: −29.55, −14.90), low-density lipoproteins (−17.72 mg/dl; 95% CI: −25.23, −10.22), and FBS (−6.75 mg/dl; 95% CI: −10.67, −2.84). Probiotics lowered ALT (−14.46 IU/L; 95% CI: −21.33, −7.59) and triglycerides (−20.97 mg/dl; 95% CI: −40.42, −1.53) the most. None had significant impact on BMI, high-density lipoproteins, and HOMA-IR changes.

**Conclusion:**

Synbiotics and probiotics are likely to be the most potential effective treatments for AST and ALT reduction in adult patients with NAFLD, respectively. Although liver enzymes cannot exactly define the severity of NAFLD, unlike the results from biopsy or imaging tests, they are important indicators that can monitor the status of the disease and provide benefits for clinical management.

**Systematic Review Registration:**

[https://www.crd.york.ac.uk/prospero/display_reco rd.php?ID], identifier [CRD42020200301].

## Introduction

Non-alcoholic fatty liver disease (NAFLD) is a chronic fatty liver disease found in approximately 25% of the population worldwide (1). The incidence of NAFLD varied from 19 to 86 per 1,000 person-year (2). Patients with a metabolic syndrome are considered a high-risk group facing NAFLD (3). NAFLD covers both non-alcoholic fatty liver (NAFL) and non-alcoholic steatohepatitis (NASH). NAFL is defined as the presence of hepatic steatosis without hepatocellular injury, whereas NASH is a NAFL with hepatocellular injury which may involve fibrosis. NAFLD can lead to other severe diseases such as cirrhosis, liver failure, liver cancer, and non-liver-related conditions, e.g., cardiovascular diseases, chronic kidney disease, etc. (3–7). In order to prevent complications and treat the disease, the etiology and pathophysiology of it should be understood. The mechanism of NAFLD involves various pathways, including gut microbiota. Its association with liver disease has been demonstrated through animal models. The samples that were intervened with antimicrobials and controls were compared to investigated gut microbial metabolic phenotypes. Notably, more than 200 microbial-related metabolites were identified in fingerprints of urine and feces of animals exposed to antimicrobials (8). Some of microbiota-derived metabolites may trigger hepatic metabolism alteration and inflammatory reaction (9). Although the issue on a relationship between liver and intestine is not fully clarified, various studies showed that dysbiosis results in malfunction of hepatic fat deposition (10, 11).

Currently, the only treatments for NAFLD recommended in the guidelines (3, 5, 6) are lifestyle modifications including diet control, exercise, and weight reduction. These methods, especially weight reduction, are hard to achieve and maintain. All other pharmacological treatments are reserved for patients with biopsy-proven NASH and liver fibrosis. According to previous studies, numerous pathophysiologic mechanisms relating the gut microbiome and NAFLD have been indicated, including the dysbiosis-induced dysregulation of the gut endothelial barrier function that allows for the translocation of bacterial components, leading to the accumulation fat and hepatic inflammation (12, 13). Thus, using microbial therapy, including synbiotics, probiotics, and prebiotics, may help to restore the unbalanced microbiomes. Also, as proven by many randomized controlled trials (RCTs), microbial therapy is classified as one of the non-pharmacological treatments which may provide the clinical benefit of slowing down the progression of NAFLD. Nevertheless, the recommendation of using these agents in clinical practice is still inconclusive (5). The primary objective of this study was to compare the effects among synbiotics, probiotics, and prebiotics by focusing on the modification of liver enzymes, including aspartate aminotransferase (AST) and alanine aminotransferase (ALT), in patients with NAFLD. Moreover, for the secondary objectives, we explored the effects of microbial therapies on body mass index (BMI), waist circumference (WC), lipid profile, fasting blood sugar (FBS), and homeostatic model assessment-insulin resistance (HOMA-IR) in patients with NAFLD.

## Methods

### Protocol and Registration

A systematic review and network meta-analysis (NMA) were performed and reported according to the Preferred Reporting Items for Systematic Reviews and Meta-Analyses (PRISMA) extension statement for NMA (14). This study was registered with the trial registration number CRD42020200301 under the international prospective register of systematic reviews (PROSPERO: www.crd.york.ac.uk/PROSPERO).

### Eligibility Criteria

The RCTs that included participants with NAFLD and that which compared the effects of synbiotics, probiotics, or prebiotics against each other or with a placebo were included in the analysis. The diagnosis method of NAFLD was not restricted only to liver biopsy. Reliable imaging techniques such as ultrasound, transient elastography (Fibroscan), and proton density fat fraction on magnetic resonance imaging (MRI-PDFF) were also acceptable to include in the analyses. The primary interested effects of the interventions were the reduction of AST and ALT since they were basic biomarkers that could be used to monitor the severity of the disease. Furthermore, the studies that showed the results in other secondary outcomes that consisted of BMI, WC, lipid profile, FBS, and/or HOMA-IR were included. Our protocol had no limitations on the length of follow-up period for each trial in the inclusion criteria. We excluded studies that consisted of only abstracts presented at conferences, along with editorials, any type of reviews, and meta-analyses.

### Information Sources and Search Strategy

We searched for relevant published articles from five electronic databases, namely, PubMed, the Web of Science, Embase, CLINAHL Plus, and the Cochrane Library, from the inception of the databases to February 2, 2022. The keywords included “synbiotic,” “probiotic,” “Lactobacillus*,” “Bifidobacterium*,” “*Enterococcus faecium*,” “*Streptococcus thermophiles*,” “*Bacillus clausii*,” “*Saccharomyces cerevisiae*,” “*Saccharomyces boulardii*,” “*Escherichia coli* Nissle 1917,” “prebiotic,” “FOS,” “Fruc-tooligosaccharide*,” “Fructo-oligosaccharide*,” “GOS,” “Galactooligosaccharide*,” “Galacto-oligosaccharide*,” “XOS,” “Xylooligosaccharide*,” “Xylo-oligosaccharide*,” “TOS,” “Transgalactooligosaccharide*,” “*Trans-*galactooligosaccharide*,” “Inulin,” “Lactitol,” “Lactulose,” “Lactosucrose,” “Soy oligosaccharide*,” “NAFLD,” “NASH,” “Fatty liver*,” “Non-alcoholic fatty liver disease,” “Non-alcoholic fatty liver disease,” “Non-alcoholic fatty liver disease,” “Non-alcoholic fatty liver*,” and “Non-alcoholic steatohepati*.” Bibliographic lists of related articles were also explored. The complete search strategy is provided in the Supplementary Appendix 1.

### Study Selection

Four investigators independently screened the titles and the abstracts of the retrieved citations to identify potentially eligible studies. Only English articles were included. Any conflict was resolved through a subsequent team discussion and an expert consultation. Adults and children with the disease have different characteristic (15). Also, the interventions might act differently regarding age of the patients and there was a limited number of studies in children. Our network meta-analysis would only include adult patients with NAFLD. The data from studies involving with pediatric patients would be extracted, summarized, and reported descriptively.

### Data Extraction and Study Appraisal

Each potentially relevant study was accessed in a full-text manner against the eligible criteria and then adopted in a data-extraction process by the same four investigators. Any inconsistent opinion along this process was settled through a discussion. We extracted the data, including the study design, the details of the interventions, such as the regimens and treatment durations, the study size, and the population characteristics and treatments’ outcomes, i.e., the reported mean and/or standard deviation (SD) values of age, AST, ALT, BMI, WC, total cholesterol (TC), triglycerides (TG), low-density lipoproteins (LDL), high-density lipoproteins (HDL), FBS, and HOMA-IR, which were the representative parameters of the effects of the interventions. When mean and/or SD were not reported, continuous outcomes were estimated by using the reported statistics (e.g., median, interquartile range, etc.) (16). Furthermore, we had contacted study authors to acquire the missing outcomes of pertinent studies. However, if the authors did not respond within a month, the study was, then, excluded from the analyses.

### Risk-of-Bias Assessments

The risk of bias in each individual study was assessed independently by four investigators using the instructions from the revised Cochrane risk-of-bias tool for randomized trials (RoB 2.0) (17). This tool addresses specific bias domains, including methods for generating the random sequence, allocation concealment, blinding of participants and investigators, blinding of the outcome assessment, incompleteness of the outcome data, and selective outcome reporting. Each item is adjudicated within each study, and the results are represented in the risk-of-bias summary graph and risk-of-bias summary itself. The adjudication of the risk of bias was achieved by answering pre-specified questions about the methods reported by each study in relation to the risk domain, such that the conclusion consists of a low risk of bias, an unclear risk of bias, or a high risk of bias. All disagreements among four investigators were resolved by consensus or with the consultation of the expert.

### Outcomes and Definitions

The primary outcomes were the effects of synbiotics, probiotics, and prebiotics on the reduction of the AST and ALT levels in patients with NAFLD. The secondary outcomes were the effects of synbiotics, probiotics, and prebiotics on patients’ BMI, WC, lipid profiles (i.e., TC, TG, LDL, and HDL), FBS, and HOMA-IR. The definitions of NASH that would be later used to classify patients for sensitivity analyses were given according to what was defined in the included studies. Those studies which did not obviously specify that they included patients with NASH in the trial would be categorized as the studies which were conducted in patients with NAFLD (5).

### Synthesis and Statistical Analysis

First, we conducted pairwise meta-analyses by using the DerSimonian and Laird random effects model (18) to estimate the outcomes. Then, we reported them in weighted mean differences and 95% confidence intervals (95% CIs). We assessed the statistical heterogeneity in each pairwise comparison by using *I*-squared statistic and Chi-squared statistic. Heterogeneity was indicated when the *p*-value was less than 0.1. We also performed a random-effects NMA to combine direct and indirect evidence of all relative options effects by using the *network* command in the Stata Statistical Software: Release 16 (StataCorp LP, College Station, TX, United States) and the methods of the NMA described by Lu and Ades (19). To rank the options hierarchy of competing for intervention in the NMA, the rankogram, the surface under the cumulative ranking (SUCRA) curves, the mean ranks, and the league tables were used (20). Network inconsistency between direct and indirect evidence was assessed using a global inconsistency test (*p*-value ≥ 0.05 indicated consistency). We also used a comparison-adjusted funnel plot to detect any small-study effects and publication bias.

In addition, to determine whether the results were affected by the variety in the studies’ characteristics, we also performed sensitivity analyses, focusing on the above-mentioned outcomes of synbiotics, probiotics, and prebiotics. Multiple sensitivity analyses were performed to assess the robustness of the findings. These were based on (1) the subgroup of participants with liver biopsy-proven NASH and (2) the duration of treatment that were less than and at least 12 weeks in patients with NAFLD and patients with NASH. We use two-sided statistical testing with *p*-values < 0.05 to indicate the statistical significance.

## Results

**FIGURE 1:**
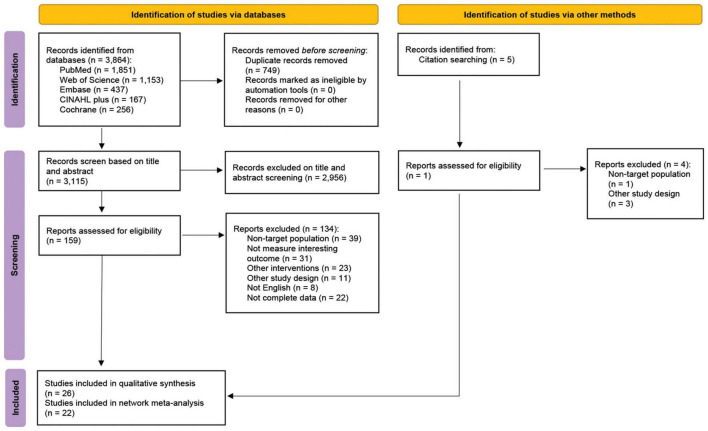
Preferred reporting items for systematic review and meta-analyses (PRISMA) flow diagram.

A total of 3,864 articles were identified from PubMed, the Web of Science, Embase, CLINAHL Plus, and the Cochrane Library. Seven-hundred and forty-nine duplicated articles were removed. The full texts of 159 articles were assessed and 134 studies were excluded due to the reasons described in Figure 1. In addition, 1 RCT identified from reference lists was included. Ultimately, we obtained 26 eligible articles: 22 RCTs were performed focusing on the adult patients with NAFLD (21–42), and the other 4 RCTs were performed concerning the pediatric patients with NAFLD (43–46; Table 1). The study-selection-process flow is summarized in the PRISMA flow diagram (Figure 1).

**TABLE 1:** Details of included trials.

ID	First author, publication year	Country	Studied population	Age (years)	Diagnosis	Study design	Interventions	Sample size	Treatment duration (weeks)	Outcomes
1	Aller et al. (21)	Spain	Adults, NAFLD	29–60	Liver biopsy	Double-blind RCT	Probiotics	14	12	AST, ALT, BMI, TC, TG, LDL, HDL, FBS, HOMA-IR
							Placebo	14		
2	Vajro et al. (43)	Italy	Children, NAFLD	11 ± 2	Ultrasound	Pilot double-blind RCT	Probiotics	10	8	ALT, BMI
							Placebo	10		
3	Malaguarnera et al. (22)	Italy	Adults, NASH	30–65	Liver biopsy	Double-blind RCT	Synbiotics	34	24	AST, ALT, BMI, TC, TG, LDL, HDL, FBS, HOMA-IR
							Placebo	32		
4	Wong et al. (23)	Hong Kong	Adults, NASH	18–70	Liver biopsy	Open-label RCT	Probiotics	10	24	AST, ALT, BMI, WC, TC, TG, LDL, HDL, FBS
							Placebo	10		
5	Alisi et al. (44)	Italy	Children, NAFLD	6–12	Liver biopsy	Double-blind RCT	Probiotics	22	16	ALT, BMI, TG, HOMA-IR
							Placebo	22		
6	Eslamparast et al. (24)	Iran	Adults, NAFLD	≥18	Fibroscan	Double-blind RCT	Synbiotics	26	28	AST, ALT, HOMA-IR
							Placebo	26		
7	Miccheli et al. (45)	Italy	Children, NAFLD	6–12	Ultrasound	Double-blind RCT	Probiotics	15	16	AST, ALT, BMI, TC, TG, LDL, HDL, FBS, HOMA-IR
							Placebo	16		
8	Sepideh et al. (25)	Iran	Adult, NAFLD	18–65	Ultrasound	Double-blind RCT	Probiotics	21	8	FBS, HOMA-IR
							Placebo	21		
9	Akbarzadeh et al. (26)	Iran	Adults, NAFLD	18–77	Fibroscan	Double-blind RCT	Prebiotics	38	10	AST, ALT, BMI, WC
							Placebo	37		
10	Asgharian et al. (27)	Iran	Adults, NAFLD	18–60	Ultrasound	Double-blind RCT	Synbiotics	38	8	AST, ALT, BMI, WC
							Placebo	36		
11	Ekhlasi et al. (28)	Iran	Adults, NAFLD	25–64	Ultrasound	Double-blind RCT	Synbiotics	15	8	AST, ALT, BMI, WC, TC, TG, LDL, HDL, FBS, HOMA-IR
							Placebo	15		
12	Ferolla et al. (29)	Brazil	Adults, NASH	25–74	Liver biopsy	Double-blind RCT	Synbiotics	27	12	AST, ALT, BMI, WC, TC, TG, LDL, HDL, FBS
							Placebo	23		
13	Asgharian et al. (30)	Iran	Adults, NAFLD	18–60	Ultrasound	Double-blind RCT	Synbiotics	38	8	BMI, WC, TC, TG, LDL, HDL, FBS
							Placebo	36		
14	Behrouz et al. (31)	Iran	Adults, NAFLD	20–60	Ultrasound	Double-blind RCT	Probiotics	30	12	BMI, FBS, HOMA-IR
							Prebiotics	29		
							Placebo	30		
15	Famouri et al. (46)	Iran	Children, NAFLD	10–18	Ultrasound	Triple-blind RCT	Probiotics	32	12	AST, ALT, WC, TC, TG, LDL, HDL
							Placebo	32		
16	Javadi et al. (32)	Iran	Adults, NAFLD	20–60	Ultrasound	Double-blind RCT	Synbiotics	17	12	AST, ALT, BMI
							Probiotics	20		
							Prebiotics	19		
							Placebo	19		
17	Javadi et al. (33)	Iran	Adults, NAFLD	20–60	Ultrasound	Double-blind RCT	Synbiotics	17	12	BMI, WC, TC, TG, LDL, HDL, FBS, HOMA-IR
							Probiotics	20		
							Prebiotics	19		
							Placebo	19		
18	Manzhalii et al. (34)	Ukraine	Adults, NASH	30–60	Ultrasound and elevated hepatic enzymes	Non-blinded RCT	Synbiotics	38	12	AST, ALT, BMI, TC, TG, LDL, FBS
							Placebo	37		
19	Mofidi et al. (35)	Iran	Adults, NAFLD	≥18	Fibroscan	Double-blind RCT	Synbiotics	21	28	AST, ALT, TC, TG, LDL, HDL, FBS, HOMA-IR
							Placebo	21		
20	Monem et al. (36)	Egypt	Adults, NASH	44 ± 6	Liver biopsy	RCT	Probiotics	15	4	AST, ALT
							Placebo	15		
21	Bakhshimoghaddam et al. (37)	Iran	Adults, NAFLD	≥18	Ultrasound	Open-label RCT	Synbiotics	34	24	AST, ALT, HOMA-IR
							Placebo	34		
22	Ahn et al. (38)	South Korea	Adults, NAFLD	19–75	MRI-PDFF	Double-blind RCT	Probiotics	30	12	AST, ALT, BMI, TC, TG, HDL, FBS, HOMA-IR
							Placebo	35		
23	Duseja et al. (39)	India	Adults, NAFLD	≥18	Liver biopsy	Double-blind RCT	Probiotics	17	48	AST, ALT
							Placebo	13		
24	Abhari et al. (40)	Iran	Adults, NAFLD	18–75	Fibroscan	Double-blind RCT	Synbiotics	22	12	AST, ALT, BMI, WC, TC, TG, LDL, HDL, FBS, HOMA-IR
							Placebo	24		
25	Behrouz et al. (41)	Iran	Adults, NAFLD	20–60	Ultrasound	Double-blind RCT	Probiotics	30	12	AST, ALT, BMI, WC, TC, TG, LDL, HDL, FBS
							Prebiotics	29		
							Placebo	30		
26	Chong et al. (42)	United Kingdom	Adults, NAFLD	25–70	Liver biopsy	Double-blind RCT	Probiotics	19	10	AST, ALT, TC, TG, LDL, HDL, HOMA-IR
							Placebo	16		

*ALT, alanine aminotransferase; AST, aspartate aminotransferase; BMI, body mass index; FBS, fasting blood sugar; HDL, high-density lipoproteins; HOMA-IR, homeostatic model assessment-insulin resistance; LDL, low-density lipoproteins; NAFL, non-alcoholic fatty liver; NASH, non-alcoholic steatohepatitis; MRI-PDFF, proton density fat fraction on magnetic resonance imaging; RCT, randomized controlled trial; TC, total cholesterol; TG, triglycerides; WC, waist circumference.*

### Characteristics and Quality of the Included Studies

**FIGURE 2:**
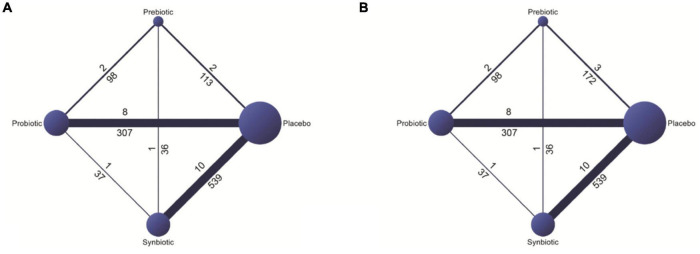
Networks of all options comparisons for reduction in **(A)** aspartate aminotransferase (AST) and **(B)** alanine aminotransferase (ALT).

The included studies are comprised of 1,389 participants with NAFLD (1,230 adults, age ≥ 18 years and 159 children, age 6–18 years). Of 1,230 adults with NAFLD, 241 were confirmed as NASH by either liver biopsy or ultrasound. Liver biopsy was done in 8 of 26 RCTs (21–23, 29, 36, 39, 42, 44). Others were diagnosed the disease by ultrasound (13 studies), Fibroscan (4 studies), and MRI-PDFF (1 study). Four studies that involved pediatric patients (age < 18 years) focused only on the effects of probiotics (43–46). Otherwise, the studies involving adult patients focused on probiotics, prebiotics, or synbiotics. The probiotics assessed in this systematic review included *Lactobacillus* spp., *Bifidiobacterium*spp., *Streptococcus thermophilies*, and *Pediococcuspentosaceus*. Included prebiotics were fructooligosaccharides, inulin, and oligofructose. Synbiotics were defined as interventions when they contained both probiotics and prebiotics. Details of the general characteristics of all included microbial therapy interventions are given in the Supplementary Appendix 2. The duration of treatment varied between 4 and 48 weeks. The details regarding the interventions and the baseline characteristics of included patients in each study are shown in the Supplementary Appendices 3, 4, respectively. The networks of all option comparisons for primary and secondary outcomes were illustrated in Figure 2 and Supplementary Appendix 5, respectively. A quality assessment of the risk of bias revealed some concern in most of the studies. There were 6 RCTs considered as having a low risk of bias (21, 29, 32, 33, 45, 46) and 3 RCTs had a high risk of bias (26, 34, 37), while the rest (17 studies) was categorized as moderate-risk studies (Supplementary Appendix 6). All data extracted for systematic review and network meta-analyses were detailed in Supplementary Appendices 7, 8.

### Pediatric Patients With Non-alcoholic Fatty Liver Disease

#### Primary Outcomes

##### Aspartate Aminotransferase and Alanine Aminotransferase [4 Studies]

Only two studies conducted by Miccheli et al. (45) and Famouri et al. (46) investigated the effect of probiotics on AST change in pediatric patients. Both studies indicated AST level was significantly reduced after the treatment as compared to the level of the enzyme at baseline. Moreover, the effect on AST lowering were significantly greater in probiotics group than placebo group. All 4 studies focusing on pediatric patients with NAFLD (43–46) evaluated the ALT change, but 2 out of 4 reported that probiotics might not be capable to reduce ALT level compared with a placebo (44, 45).

#### Secondary Outcomes

##### Body Mass Index [4 Studies]

Half of the studies showed that probiotics did not lower the BMI of the pediatric patients with NAFLD (43, 46). The other two studies (44, 45), which conducted in the same cohort of patients, indicated that BMI of the intervention group was significantly lowered at the end of the trial.

##### Waist Circumference [1 Study]

Only one study by Famouri et al. (46) measured the effect of probiotics on WC change in children with obesity who were diagnosed with NAFLD. They reported that probiotics had a significant effect on WC reduction, as compared to a placebo.

##### Lipid Profile [Total Cholesterol: 2 Studies, Triglycerides: 3 Studies, Low-Density Lipoproteins: 2 Studies, and High-Density Lipoproteins: 2 Studies]

The study by Miccheli et al. (45) pointed out that probiotics did not have an impact on TC. In addition, even if Famouri et al. (46) reported that their intervention could significantly reduce TC, a median baseline TC level of the control group was significantly lower than the probiotics group.

All tree studies by Alisi et al. (44), Miccheli et al. (45), and Famouri et al. (46) concluded that probiotics did not provide any additional benefit over a placebo in TG reduction among obese children with NAFLD.

Micheli et al. (45) did not see the effect of probiotics on LDL lowering. Nonetheless, the median LDL of the intervention group of the study by Famouri et al. (46) was significantly lower at the end of the trial than the value at the baseline. Moreover, the magnitude of LDL reduction in the intervention group was larger than the control group.

Both trials by Miccheli et al. (45) and Famouri et al. (46) did not observe any significant change in HDL level of the participants.

##### Fasting Blood Sugar and Homeostatic Model Assessment-Insulin Resistance [1 Study]

Only one of four included studies in children investigated the effect of probiotics on diabetes-related outcomes. Miccheli et al. (45) could not conclude any benefit of probiotics based on the outcomes of the trial.

### Adults Patients With Non-alcoholic Fatty Liver Disease

#### Primary Outcomes

##### Aspartate Aminotransferase and Alanine Aminotransferase

###### Adults With Non-alcoholic Fatty Liver Disease [18 Studies]

**FIGURE 3:**
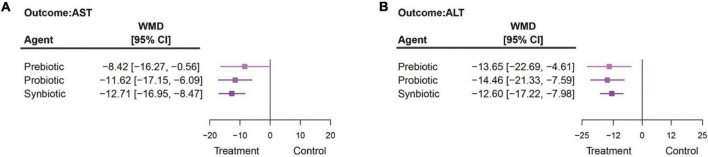
The summarized results of using synbiotics, probiotics, and prebiotics for the reduction in **(A)** AST and **(B)** ALT.

Our NMA found that when compared with a placebo, all three interventions significantly decreased the levels of both AST and ALT. Synbiotics provided the best effect on AST. They reduced the AST by −12.71 IU/L (95% CI: −16.95, −8.47). The second and third best interventions were probiotics (AST: −11.62 IU/L; 95% CI: −17.15, −6.09) and prebiotics (AST: −8.42 IU/L; 95% CI: −16.27, −0.56), respectively (Figure 3A). When the interventions were compared against each other, there was no specific intervention that could be considered better than another (Supplementary Appendix 9).

Probiotics provided the most impact on ALT reduction when compared with placebo (ALT: −14.46 IU/L; 95% CI: −21.33, −7.59). Synbiotics and prebiotics significantly reduced ALT by −12.60 IU/L (95% CI: −17.22, −7.98) and −13.65 IU/L (95% CI: −22.69, −4.61), respectively (Figure 3B). When compared among interventions, the statistical difference did not show in any pair of interventions (Supplementary Appendix 9).

When interventions, including placebos, were compared with one another, as shown in SUCRA, synbiotics had the highest likelihood of being ranked first in the analysis of the effects on AST reduction, followed by probiotics, prebiotics, and placebo (Supplementary Appendix 10). Contrastingly, SUCRA showed that probiotics had the highest likelihood of being ranked first for ALT reduction, followed by prebiotics and synbiotics. The results indicated no possibility that placebo would provide better outcomes than other interventions (Supplementary Appendix 10).

###### Adults With Biopsy-Proven Non-alcoholic Steatohepatitis [4 Studies]

In the subgroup of patients with biopsy-proven NASH, synbiotics provided the best effects, in terms of AST reductions when compared to placebo (−22.34 IU/L; 95% CI: −38.02, −6.67). However, when synbiotics were compared against probiotics, no significance difference of AST reductions was seen. Probiotics had the most impact on ALT reduction in this subgroup. It significantly decreased more ALT than both placebo (−34.10 IU/L; 95% CI: −46.43, −21.77) and synbiotics (−17.70 IU/L; 95% CI: −34.61, −0.79). Synbiotics also significantly reduced ALT in patients with biopsy-proven NASH. When compared with a placebo, they reduced ALT by −16.40 IU/L (95% CI: −27.96, −4.83). More details were shown in Supplementary Appendices 13, 14.

Further results of the sensitivity analyses, which were restricted to the effects of interventions in the studies in which durations of treatments were less than 12 weeks and at least 12 weeks, separately, are presented in Supplementary Appendices 13, 14. Most of the sensitivity analyses showed similar results to the main analyses. Particularly, the interventions could significantly reduce hepatic enzymes when compared with placebo. However, there was no specific intervention that could considered better than the others in terms of liver enzymes reductions. Prebiotics provided the lowest magnitude of effect on AST reduction. All three microbial treatments did not provide significant effect on AST level among patients with NAFLD compared to a placebo unless the treatments were given at least 12 weeks. Both probiotics and synbiotics significantly reduced AST in patients with NASH who were treated for not less than 12 weeks, but only synbiotics could significantly decrease ALT in this subgroup.

#### Secondary Outcomes

##### Body Mass Index [13 Studies]

**FIGURE 4:**
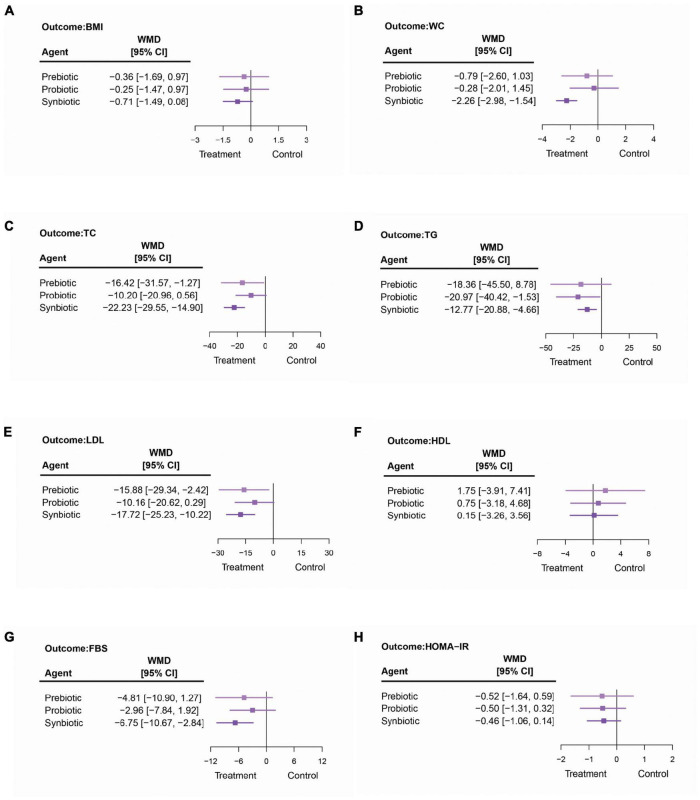
The summarized results of using synbiotics, probiotics, and prebiotics for the modifications in **(A)** body mass index (BMI), **(B)** waist circumference (WC), **(C)** total cholesterol (TC), **(D)** triglycerides (TG), **(E)** low-density lipoproteins (LDL), **(F)** high-density lipoproteins (HDL), **(G)** fasting blood sugar (FBS), and **(H)** homeostatic model assessment-insulin resistance (HOMA-IR).

The pooled results showed that the interventions did not have a significant impact on BMI in adult patients with NAFLD, as shown in Figure 4A and Supplementary Appendix 9. The results from subgroup among patients with biopsy-proven NASH also showed no statistically different effect when a comparison was made between interventions and placebo. The sensitivity analyses, including the analyses among adult patients who were treated for not less than 12 weeks, revealed no statistical differences between all pairs of options. The details are shown in Supplementary Appendix 15.

##### Waist Circumference [8 Studies]

Among three microbial therapies, only synbiotics significantly reduced the WC of adults with NAFLD (synbiotics vs. placebo: −2.26 cm; 95% CI: −2.98, −1.54 and synbiotics vs. probiotics: −1.98 cm; 95% CI: −3.84, −0.11), as shown in Figure 4B and Supplementary Appendix 9. Nonetheless, this statistically significant result was not seen in any sensitivity analysis. Further details are shown in Supplementary Appendix 16.

##### Lipid Profile [Total Cholesterol: 13 Studies, Triglycerides: 13 Studies, Low-Density Lipoproteins: 11 Studies, and High-Density Lipoproteins: 12 Studies]

Synbiotics had significant effects on TC, TG, and LDL reduction when compared with a placebo (TC: −22.23 mg/dl; 95% CI: −29.55, −14.90; TG: −12.77 mg/dl; 95% CI: −20.88, −4.66; and LDL: −17.72 mg/dl; 95% CI: −25.23, −10.22), as shown in Figures 4C–E. When compared among the interventions, there was no specific one that could be considered significantly better than others (Supplementary Appendix 9). Prebiotics also significantly decreased TC by −16.42 mg/dl (95% CI: −31.57, −1.27) and LDL by −15.88 mg/dl (95% CI: −29.34, −2.42) more than placebo. Probiotics provided the largest impact on TG reduction (−20.97 mg/dl, 95% CI: −40.42, −1.53), but did not have an effect on other parameters related to patients’ lipid profile. Moreover, this NMA showed that neither prebiotics, probiotics, nor synbiotics had an effect on increasing the HDL level (Figure 4F). The results are shown in Supplementary Appendix 9.

Surprisingly in the sensitivity analysis involving the biopsy-proven NASH, probiotics provided a significant reduction of the HDL level (−3.86 mg/dl; 95% CI: −7.25, −0.47), but this only involved one study. Nevertheless, the other microbial therapies did not show the significant effects on HDL, TC, TG, and LDL in patients with biopsy-proven NASH. The analyses of the studies that treated the patients for at least 12 weeks demonstrated that when compared to a placebo, synbiotics could significantly reduce the TC, TG, and LDL levels among patients with NAFLD (TC −18.04 mg/dl; 95% CI: −33.00, −3.09; TG: −16.16 mg/dl; 95% CI: −31.42, −0.90; and LDL: −14.85 mg/dl; 95% CI: −26.31, −3.38). Probiotics significantly reduced TG by −25.34 mg/dl (95% CI: −46.42, −4.27) and LDL by −11.88 mg/dl (95% CI: −21.69, −2.08) when compared to a placebo. When treated with prebiotics for at least 12 weeks, the pooled outcomes showed that in adult patients with NAFLD, prebiotics could reduce TC by −16.04 mg/dl (95% CI: −32.03, −0.05), and LDL by −16.40 mg/dl (95% CI: −27.16, −5.63) compared to a placebo. Among the studies with treatment duration of less than 12 weeks, only synbiotics could significantly lower TC, TG, and LDL in patients with NAFLD when compared to a placebo. Other details are shown in Supplementary Appendix 17.

##### Fasting Blood Sugar [14 Studies]

The pooled outcomes showed that synbiotics were the only intervention that significantly lowered the FBS by −6.75 mg/dl (95% CI: −10.67, −2.84) in patients with NAFLD when compared to a placebo (Figure 4G and Supplementary Appendix 9). Nonetheless, when compared to another microbial therapy, synbiotics did not provide any additional favorable effect on FBS. The sensitivity analyses demonstrated that in both groups of cohorts treated with at least 12 weeks and less than 12 weeks of the interventions, synbiotics would still be the only treatment option that provided a significant effect, particularly when comparing their effect with a placebo in adults with NAFLD. Also regarding the sensitivity analysis, none of the interventions had an effect on FBS, specifically for patients with NASH. The magnitudes of effects are shown in Supplementary Appendix 18.

##### Homeostatic Model Assessment-Insulin Resistance [12 Studies]

Both main and sensitivity analyses showed that there was no significant difference in the HOMA-IR change in any pair of the options; neither when compared with a placebo nor among interventions (Figure 4H and Supplementary Appendix 9). Further details can be found in Supplementary Appendix 19.

**FIGURE 5:**
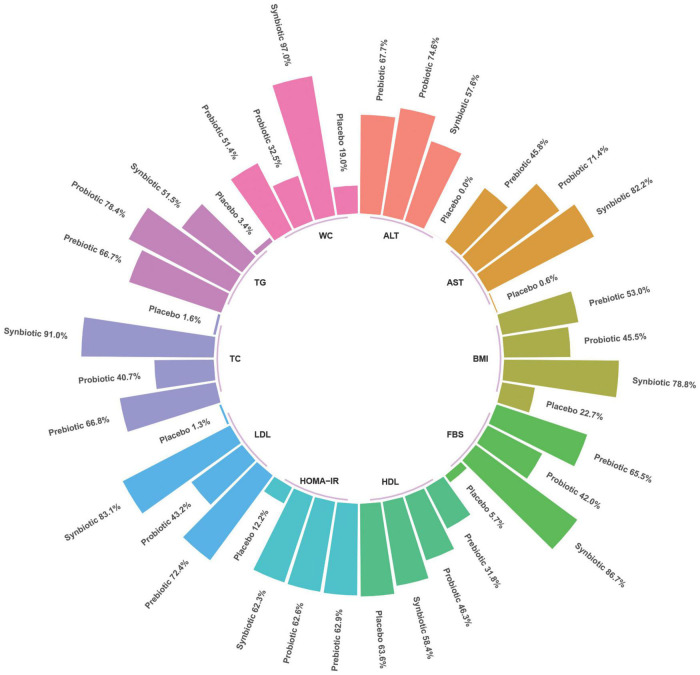
Rank-bar chart with surface under the cumulative ranking (SUCRA) values for outcomes associated with synbiotics, probiotics, and prebiotics use in patients with non-alcoholic fatty liver disease (NAFLD).

The rank-bar chart which illustrated SUCRA cumulative probabilities of all outcomes associated with synbiotics, probiotics, prebiotics, and placebo used in patients with NAFLD are illustrated in Figure 5.

### Network Consistency and Small-Study Effects

There was no evidence of any inconsistency in the results of our network meta-analysis. The results of the global-inconsistency assessment are shown in the Supplementary Appendix 11. The comparison-adjusted funnel plots revealed no evidence of small-study effects for AST, ALT, BMI, WC, TC, TG, FBS, and HOMA-IR, but there was evidence of small-study effects on LDL and HDL outcomes (Supplementary Appendix 12).

## Discussion

This systematic review summarized the data from 26 RCTs by comparing the effects of synbiotics, probiotics, and prebiotics in 1,389 patients with NAFLD. Trials conducted in adult and pediatric patients were separately analyzed and reported. The number of studies in pediatric patients was too small to draw any conclusion about the effect of probiotics on NAFLD. Additionally, network meta-analyses were performed to demonstrate the pooled outcomes related to NAFLD among adult patients. There was no evidence of inconsistency in our analysis. Thus, we compared the effects of synbiotics, probiotics, and prebiotics by using a consistency model. Our primary findings were that when compared to a placebo, all three interventions could significantly reduce AST and ALT. The effects of liver enzymes reduction in patients with NAFLD when microbial therapy was competed with one another was inconclusive. According to the results, there was no specific intervention that could be considered better than others. The sensitivity analyses showed similar effects. However, no study had investigated the effect of prebiotics on hepatic enzymes, particularly in patients with NASH. Interestingly, probiotics did provide a significantly superior ability to decrease ALT as compared to synbiotics among patients with biopsy-proven NASH.

The secondary outcomes showed that some interventions might improve WC, lipid profile (only TC, TG, and LDL), and FBS of patients with NAFLD. Synbiotics showed significant effects in most biomarkers including WC, TC, LDL, and FBS. Probiotics could lower only TG in adults with NAFLD. In addition, prebiotics provided the abilities of TC and LDL decrements. Neither of the interventions increased the HDL level of patients.

Regarding AST and ALT reductions, when sensitivity analyses were performed on studies which included only patients with biopsy-proven NASH, the significant results were only seen in synbiotics and probiotics. Furthermore, when sensitivity analyses were exclusively done in trials that examined the effects of interventions which were given at least a 12 week-duration, the microbial therapies significantly performed better than a placebo in most outcomes (i.e., AST, ALT, TC, TG, LDL, and FBS).

Our results are mostly consistent with those of previous studies (47–50) which have demonstrated a significant reduction of AST and ALT by microbial therapies in patients with NAFLD, though our systematic review and NMA included more up-to-date RCTs with an overall larger sample size than previous meta-analyses. Five new RCTs were reported after the latest meta-analysis of the efficacy of microbiome-targeted therapies in NAFLD by Sharpton et al. (49) was published. A meta-analysis by Loman et al. (47) indicated that only prebiotics and probiotics, but not synbiotics, significantly decrease ALT in patients with NAFLD. The significant benefit of synbiotics in ALT modification was additionally seen in our present analysis. Moreover, Loman et al. demonstrated that all three microbial interventions could significantly decrease BMI in patients with NAFLD. However, our study showed that when incorporating indirect effects in the analysis, none of the interventions was considered to be an effective treatment for BMI reduction. Currently, the mechanism underlying NAFLD in human is not clearly known and varies with regard to the disease heterogeneity. However, one of the etiologic pathways that has been demonstrated in pre-clinical models is involved with gut microbiota (9–11).

This NMA has several strengths. First, we included both direct and indirect evidence of all comparisons relating to the interested outcomes. Second, we only included RCTs to compare the effects of synbiotics, probiotics, and prebiotics. Finally, sensitivity analyses were performed for every outcome associated with NAFLD. They were likely to yield similar results as those from the main analysis. This confirms the robustness of the study.

There were a few limitations in this study. First, the number of studies focusing on pediatric patients was too small to be pooled and to summarize the effects of microbial therapies on the interested outcomes. The sample size of adult patients was also relatively small for an NMA. Second, we did not explore the effect of each subtype of microbial therapy or the relative dose-response relationship, which may have affected the results. There were multiple types of microbial therapies and dosage recommendations. Furthermore, the dosage varied depending on a type of microbial therapy. We were not able to perform subgroup analyses due to a limited number of studies. However, according to results from the test of global inconsistency, they indicated no heterogeneity. Hence, we could infer that even if there were variations in type and dosage, the effect sizes and outcomes might be interpreted the same way as they were. On the other hand, these results should be able to apply in general. Third, most studies were considered to have at least moderate risk-of-bias. Three of which were considered high-risk-of-bias studies. Finally, our outcomes of interest were surrogate outcomes, such as liver-enzyme levels, which cannot exactly define the severity, prognosis, and treatment outcomes of NAFLD. Moreover, it is important to remark that some patients may develop the disease through different pathways. Thus, the interventions may not provide good efficacy in every patient with NAFLD. Other numerous risk factors associated with NAFLD and its complications were reported, such as age, sex, ethnicity, genetic variants, comorbidities, sociocultural, and so on (9). This might lead to some difficulty of result interpretations when the data from various studies with a variety of enrolled patients were pooled together. However, these surrogate outcomes are important basic indicators that can primarily monitor status of the disease, and which should result in higher accessibility rates of early appropriate treatment for patients. Liver fibrosis is another unfavorable outcome in patients with NAFLD. Due to limited data, in this study, we did not examine the outcomes of interested interventions on liver fibrosis. In combination with other parameters, these indicators will help both the patients and clinicians make the best choices regarding treatment. Also, currently, there is no evidence pertaining to the adverse events of taking these agents. Nevertheless, we should always carefully consider every factor, including the potential benefits, risks, and costs, before deciding to use these agents.

## Conclusion

In conclusion, we found that synbiotics, probiotics, and prebiotics could significantly reduce hepatic enzymes of adult patients with NAFLD. However, the question of which microbial therapy provides the best effect on AST and ALT reduction is yet to be answered. The effect on other clinical parameters including WC, lipid profile, and FBS varied regarding types of microbial therapies. There was limited information about the efficacy of microbial therapy in pediatric patients with NAFLD.

## Data Availability Statement

The original contributions presented in the study are included in the article/Supplementary Material, further inquiries can be directed to the corresponding authors.

## Author Contributions

SK, CK, KL, MN, TS, NC, SS, and PP: study concept and design. SK, KL, MN, TS, and SS: acquisition of data. SK, CK, KL, MN, TS, and SS: analysis and interpretation of data. SK, CK, KL, MN, TS, SS, and PP: drafting of the manuscript. SK, CK, NC, SS, and PP: critical revision of the manuscript. All authors contributed to the article and approved the submitted version.

## Conflict of Interest

The authors declare that the research was conducted in the absence of any commercial or financial relationships that could be construed as a potential conflict of interest.

## Publisher’s Note

All claims expressed in this article are solely those of the authors and do not necessarily represent those of their affiliated organizations, or those of the publisher, the editors and the reviewers. Any product that may be evaluated in this article, or claim that may be made by its manufacturer, is not guaranteed or endorsed by the publisher.

## Abbreviations

ALT: alanine aminotransferase
AST: aspartate aminotransferase
BMI: body mass index
CI: confidence interval
FBS: fasting blood sugar
HDL: high-density lipoproteins
HOMA-IR: homeostatic model assessment-insulin resistance
LDL: low-density lipoproteins
NAFL: non-alcoholic fatty liver
NAFLD: non-alcoholic fatty liver disease
NASH: non-alcoholic steatohepatitis
PRISMA: preferred reporting items for systematic reviews and meta-analyses
RCTs: randomized controlled trails
SUCRA curves: surface under the cumulative ranking curves
TC: total cholesterol
TG: triglycerides
WC: waist circumference
WMD: weighted mean difference.
