# Supplementary material for: Effects of Synbiotics, Probiotics, and Prebiotics on Liver Enzymes of Patients With Non-alcoholic Fatty Liver Disease: A Systematic Review and Network Meta-Analysis
Source: Front Nutr. 2022 May 20;9:880014. doi: 10.3389/fnut.2022.880014 (PMC9167056; doi:10.3389/fnut.2022.880014)
Supplement: Supplementary file 1 [file Data_Sheet_1.pdf]

Supplementary Material

Effects of Synbiotics, Probiotics, and Prebiotics on Liver Enzymes of Patients with Non-Alcoholic Fatty Liver Disease: A Systematic Review and Network Meta-analysis

TABLE OF CONTENTS

Appendix 1: Search strategies **7**
Table S1 Search algorithms 7

Appendix 2: General characteristics of interventions **13**
Table S2 General characteristics of interventions 13

Appendix 3: Description of interventions of each included study **14**
Table S3 Description of interventions 14

Appendix 4: Baseline characteristics of included studies **19**
Table S4 Baseline characteristics of patients with non-alcoholic fatty liver disease 19

Appendix 5: Networks of all option comparisons for secondary outcomes **24**
Figure S1 Networks of all option comparisons for reduction in body mass index 24
Figure S2 Networks of all option comparisons for reduction in waist circumference 24
Figure S3 Networks of all option comparisons for reduction in total cholesterol 25
Figure S4 Networks of all option comparisons for reduction in triglycerides 25
Figure S5 Networks of all option comparisons for reduction in low-density lipoproteins 26
Figure S6 Networks of all option comparisons for increasing in high-density lipoproteins. 26
Figure S7 Networks of all option comparisons for reduction in fasting blood sugar 27
Figure S8 Networks of all option comparisons for reduction in HOMA-IR 27

Appendix 6: Risk-of-bias assessments **28**
Figure S9 Risk-of-bias summary graph 28
Figure S10 Risk-of-bias summary: judgments about each bias item for each study 29

Appendix 7: Data extracted for primary outcomes of network meta-analysis **30**
Table S5 AST and ALT changes in patients with non-alcoholic fatty liver disease 30

Appendix 8: Data extracted for secondary outcomes of network meta-analysis **32**
Table S6 Body mass index and waist circumference changes in patients with non-alcoholic fatty liver disease 32
Table S7 Lipid profile changes in patients with non-alcoholic fatty liver disease 34
Table S8 FBS and HOMA-IR changes in patients with non-alcoholic fatty liver disease 36

Appendix 9: Result of network meta-analysis **38**
Table S9 Network-estimated, weighted mean difference of options for reduction in AST (IU/L) 38
Table S10 Network-estimated, weighted mean difference of options for reduction in ALT (IU/L) 38
Table S11 Network-estimated, weighted mean difference of options for reduction in BMI (kg/m^2) 38
Table S12 Network-estimated, weighted mean difference of options for reduction in WC (cm) 38
Table S13 Network-estimated, weighted mean difference of options for reduction in TC (mg/dL) 38
Table S14 Network-estimated, weighted mean difference of options for reduction in TG (mg/dL) 39
Table S15 Network-estimated, weighted mean difference of options for reduction in LDL (mg/dL) 39
Table S16 Network-estimated, weighted mean difference of options for increasing HDL (mg/dL) 39
Table S17 Network-estimated, weighted mean difference of options for reduction in FBS (mg/dL) 39
Table S18 Network-estimated, weighted mean difference of options for reduction in HOMA-IR 39

Appendix 10: Surface under the cumulative ranking curves **40**
Figure S11 SUCRA curves for reduction in aspartate transaminase 40
Figure S12 SUCRA curves for reduction in alanine transaminase 40
Figure S13 SUCRA curves for reduction in body mass index 41
Figure S14 SUCRA curves for reduction in waist circumference 41
Figure S15 SUCRA curves for reduction in total cholesterol 42
Figure S16 SUCRA curves for reduction in triglycerides 42

Figure S17 SUCRA curves for reduction in low-density lipoproteins....43
Figure S18 SUCRA curves for increasing high-density lipoproteins....43
Figure S19 SUCRA curves for reduction in fasting blood sugar....44
Figure S20 SUCRA curves for reduction in HOMA-IR....44

Appendix 11: Assessment of inconsistency....45
Table S19 Assessment of global inconsistency in network meta-analysis....45

Appendix 12: Comparison-adjusted funnel plots....46
Figure S21 Comparison-adjusted funnel plot for the network of reduction in AST in all comparisons....46
Figure S22 Comparison-adjusted funnel plot for the network of reduction in ALT in all comparisons....46
Figure S23 Comparison-adjusted funnel plot for the network of reduction in BMI in all comparisons....47
Figure S24 Comparison-adjusted funnel plot for the network of reduction in WC in all comparisons....47
Figure S25 Comparison-adjusted funnel plot for the network of reduction in TC in all comparisons....48
Figure S26 Comparison-adjusted funnel plot for the network of reduction in TG in all comparisons....48
Figure S27 Comparison-adjusted funnel plot for the network of reduction in LDL in all comparisons....49
Figure S28 Comparison-adjusted funnel plot for the network of increasing HDL in all comparisons....49
Figure S29 Comparison-adjusted funnel plot for the network of reduction in FBS in all comparisons....50
Figure S30 Comparison-adjusted funnel plot for the network of reduction in HOMA-IR in all comparisons.50

Appendix 13: Sensitivity analyses of AST outcomes....51
Figure S31 Sensitivity analysis of the reduction in AST among biopsy-proven NASH patients. (a) network graph and (b) SUCRA curves....51
Table S20 Network-estimated, weighted mean difference of options for reduction in AST among biopsy-proven NASH patients. 51
Figure S32 Sensitivity analysis of the reduction in AST duration of treatment not less than 12 weeks among NAFLD patients. (a) network graph and (b) SUCRA curves....52
Table S21 Network-estimated, weighted mean difference of options for reduction in AST duration of treatment not less than 12 weeks among NAFLD patients....52
Figure S33 Sensitivity analysis of the reduction in AST duration of treatment less than 12 weeks among NAFLD patients. (a) network graph and (b) SUCRA curves....53
Table S22 Network-estimated, weighted mean difference of options for reduction in AST duration of treatment less than 12 weeks among NAFLD patients....53
Figure S34 Sensitivity analysis of the reduction in AST duration of treatment not less than 12 weeks among NASH patients. (a) network graph and (b) SUCRA curves....54
Table S23 Network-estimated, weighted mean difference of options for reduction in AST duration of treatment not less than 12 weeks among NASH patients. 54
Figure S35 Sensitivity analysis of the reduction in AST duration of treatment less than 12 weeks among NASH patients. (a) network graph and (b) SUCRA curves....55
Table S24 Network-estimated, weighted mean difference of options for reduction in AST duration of treatment less than 12 weeks among NASH patients....55

Appendix 14: Sensitivity analyses of ALT outcomes....56
Figure S36 Sensitivity analysis of the reduction in ALT among biopsy-proven NASH patients. (a) network graph and (b) SUCRA curves....56
Table S25 Network-estimated, weighted mean difference of options for reduction in ALT among biopsy-proven NASH patients. 56
Figure S37 Sensitivity analysis of the reduction in ALT duration of treatment not less than 12 weeks among NAFLD patients. (a) network graph and (b) SUCRA curves....57
Table S26 Network-estimated, weighted mean difference of options for reduction in ALT duration of treatment not less than 12 weeks among NAFLD patients....57
Figure S38 Sensitivity analysis of the reduction in ALT duration of treatment less than 12 weeks among NAFLD patients. (a) network graph and (b) SUCRA curves....58
Table S27 Network-estimated, weighted mean difference of options for reduction in ALT duration of treatment less than 12 weeks among NAFLD patients....58

Figure S39 Sensitivity analysis of the reduction in ALT duration of treatment not less than 12 weeks among NASH patients. (a) network graph and (b) SUCRA curves 59
Table S28 Network-estimated, weighted mean difference of options for reduction in ALT duration of treatment not less than 12 weeks among NASH patients. 59
Figure S40 Sensitivity analysis of the reduction in ALT duration of treatment less than 12 weeks among NASH patients. (a) network graph and (b) SUCRA curves 60
Table S29 Network-estimated, weighted mean difference of options for reduction in ALT duration of treatment less than 12 weeks among NASH patients. 60
Appendix 15: Sensitivity analyses of BMI outcomes 61
Figure S41 Sensitivity analysis of the reduction in BMI among biopsy-proven NASH patients. (a) network graph and (b) SUCRA curves 61
Table S30 Network-estimated, weighted mean difference of options for reduction in BMI among biopsy-proven NASH patients. 61
Figure S42 Sensitivity analysis of the reduction in BMI duration of treatment not less than 12 weeks among NAFLD patients. (a) network graph and (b) SUCRA curves 62
Table S31 Network-estimated, weighted mean difference of options for reduction in BMI duration of treatment not less than 12 weeks among NAFLD patients. 62
Figure S43 Sensitivity analysis of the reduction in BMI duration of treatment less than 12 weeks among NAFLD patients. (a) network graph and (b) SUCRA curves 63
Table S32 Network-estimated, weighted mean difference of options for reduction in BMU duration of treatment less than 12 weeks among NAFLD patients. 63
Figure S44 Sensitivity analysis of the reduction in BMI duration of treatment not less than 12 weeks among NASH patients. (a) network graph and (b) SUCRA curves 64
Table S33 Network-estimated, weighted mean difference of options for reduction in BMI duration of treatment not less than 12 weeks among NASH patients. 64
Appendix 16: Sensitivity analyses of WC outcomes 65
Figure S45 Sensitivity analysis of the reduction in WC among biopsy-proven NASH patients. (a) network graph and (b) SUCRA curves 65
Table S34 Network-estimated, weighted mean difference of options for reduction in WC among biopsy-proven NASH patients. 65
Figure S46 Sensitivity analysis of the reduction in WC duration of treatment not less than 12 weeks among NAFLD patients. (a) network graph and (b) SUCRA curves 66
Table S35 Network-estimated, weighted mean difference of options for reduction in WC duration of treatment not less than 12 weeks among NAFLD patients. 66
Figure S47 Sensitivity analysis of the reduction in WC duration of treatment less than 12 weeks among NAFLD patients. (a) network graph and (b) SUCRA curves 67
Table S36 Network-estimated, weighted mean difference of options for reduction in WC duration of treatment less than 12 weeks among NAFLD patients. 67
Figure S48 Sensitivity analysis of the reduction in WC duration of treatment not less than 12 weeks among NASH patients. (a) network graph and (b) SUCRA curves 68
Table S37 Network-estimated, weighted mean difference of options for reduction in WC duration of treatment not less than 12 weeks among NASH patients. 68
Appendix 17: Sensitivity analyses of lipid profile outcomes 69
Figure S49 Sensitivity analysis of the reduction in TC among biopsy-proven NASH patients. (a) network graph and (b) SUCRA curves 69
Table S38 Network-estimated, weighted mean difference of options for reduction in TC among biopsy-proven NASH patients. 69
Figure S50 Sensitivity analysis of the reduction in TC duration of treatment not less than 12 weeks among NAFLD patients. (a) network graph and (b) SUCRA curves 70
Table S39 Network-estimated, weighted mean difference of options for reduction in TC duration of treatment not less than 12 weeks among NAFLD patients. 70

Figure S51 Sensitivity analysis of the reduction in TC duration of treatment less than 12 weeks among NAFLD patients. (a) network graph and (b) SUCRA curves 71
Table S40 Network-estimated, weighted mean difference of options for reduction in TC duration of treatment less than 12 weeks among NAFLD patients. 71
Figure S52 Sensitivity analysis of the reduction in TC duration of treatment not less than 12 weeks among NASH patients. (a) network graph and (b) SUCRA curves 72
Table S41 Network-estimated, weighted mean difference of options for reduction in TC duration of treatment not less than 12 weeks among NASH patients. 72
Figure S53 Sensitivity analysis of the reduction in TG among biopsy-proven NASH patients. (a) network graph and (b) SUCRA curves 73
Table S42 Network-estimated, weighted mean difference of options for reduction in TG among biopsy-proven NASH patients. 73
Figure S54 Sensitivity analysis of the reduction in TG duration of treatment not less than 12 weeks among NAFLD patients. (a) network graph and (b) SUCRA curves 74
Table S43 Network-estimated, weighted mean difference of options for reduction in TG duration of treatment not less than 12 weeks among NAFLD patients. 74
Figure S55 Sensitivity analysis of the reduction in TG duration of treatment less than 12 weeks among NAFLD patients. (a) network graph and (b) SUCRA curves 75
Table S44 Network-estimated, weighted mean difference of options for reduction in TG duration of treatment less than 12 weeks among NAFLD patients. 75
Figure S56 Sensitivity analysis of the reduction in TG duration of treatment not less than 12 weeks among NASH patients. (a) network graph and (b) SUCRA curves 76
Table S45 Network-estimated, weighted mean difference of options for reduction in TG duration of treatment not less than 12 weeks among NASH patients. 76
Figure S57 Sensitivity analysis of the reduction in LDL among biopsy-proven NASH patients. (a) network graph and (b) SUCRA curves 77
Table S46 Network-estimated, weighted mean difference of options for reduction in LDL among biopsy-proven NASH patients. 77
Figure S58 Sensitivity analysis of the reduction in LDL duration of treatment not less than 12 weeks among NAFLD patients. (a) network graph and (b) SUCRA curves 78
Table S47 Network-estimated, weighted mean difference of options for reduction in LDL duration of treatment not less than 12 weeks among NAFLD patients 78
Figure S59 Sensitivity analysis of the reduction in LDL duration of treatment less than 12 weeks among NAFLD patients. (a) network graph and (b) SUCRA curves 79
Table S48 Network-estimated, weighted mean difference of options for reduction in LDL duration of treatment less than 12 weeks among NAFLD patients 79
Figure S60 Sensitivity analysis of the reduction in LDL duration of treatment not less than 12 weeks among NASH patients. (a) network graph and (b) SUCRA curves 80
Table S49 Network-estimated, weighted mean difference of options for reduction in LDL duration of treatment not less than 12 weeks among NASH patients. 80
Figure S61 Sensitivity analysis of the increasing in HDL among biopsy-proven NASH patients. (a) network graph and (b) SUCRA curves 81
Table S50 Network-estimated, weighted mean difference of options for increasing in HDL among biopsy-proven NASH patients. 81
Figure S62 Sensitivity analysis of the increasing in HDL duration of treatment not less than 12 weeks among NAFLD patients. (a) network graph and (b) SUCRA curves 82
Table S51 Network-estimated, weighted mean difference of options for increasing in HDL duration of treatment not less than 12 weeks among NAFLD patients 82
Figure S63 Sensitivity analysis of the increasing in HDL duration of treatment less than 12 weeks among NAFLD patients. (a) network graph and (b) SUCRA curves 83
Table S52 Network-estimated, weighted mean difference of options for increasing in HDL duration of treatment less than 12 weeks among NAFLD patients 83

Figure S64 Sensitivity analysis of the increasing in HDL duration of treatment not less than 12 weeks among NASH patients. (a) network graph and (b) SUCRA curves 84
Table S53 Network-estimated, weighted mean difference of options for increasing in HDL duration of treatment not less than 12 weeks among NASH patients 84
Appendix 18: Sensitivity analyses of FBS outcome 85
Figure S65 Sensitivity analysis of the reduction in FBS among biopsy-proven NASH patients. (a) network graph and (b) SUCRA curves 85
Table S54 Network-estimated, weighted mean difference of options for reduction in FBS among biopsy-proven NASH patients. 85
Figure S66 Sensitivity analysis of the reduction in FBS duration of treatment not less than 12 weeks among NAFLD patients. (a) network graph and (b) SUCRA curves 86
Table S55 Network-estimated, weighted mean difference of options for reduction in FBS duration of treatment not less than 12 weeks among NAFLD patients 86
Figure S67 Sensitivity analysis of the reduction in FBS duration of treatment less than 12 weeks among NAFLD patients. (a) network graph and (b) SUCRA curves 87
Table S56 Network-estimated, weighted mean difference of options for reduction in FBS duration of treatment less than 12 weeks among NAFLD patients 87
Figure S68 Sensitivity analysis of the reduction in FBS duration of treatment not less than 12 weeks among NASH patients. (a) network graph and (b) SUCRA curves 88
Table S57 Network-estimated, weighted mean difference of options for reduction in FBS duration of treatment not less than 12 weeks among NASH patients 88
Appendix 19: Sensitivity analyses of HOMA-IR outcomes 89
Figure S69 Sensitivity analysis of the reduction in HOMA-IR among biopsy-proven NASH patients. (a) network graph and (b) SUCRA curves 89
Table S58 Network-estimated, weighted mean difference of options for reduction in HOMA-IR among biopsy-proven NASH patients 89
Figure S70 Sensitivity analysis of the reduction in HOMA-IR duration of treatment not less than 12 weeks among NAFLD patients. (a) network graph and (b) SUCRA curves 90
Table S59 Network-estimated, weighted mean difference of options for reduction in HOMA-IR duration of treatment not less than 12 weeks among NAFLD patients 90
Figure S71 Sensitivity analysis of the reduction in HOMA-IR duration of treatment less than 12 weeks among NAFLD patients. (a) network graph and (b) SUCRA curves 91
Table S60 Network-estimated, weighted mean difference of options for reduction in HOMA-IR duration of treatment less than 12 weeks among NAFLD patients 91
Figure S72 Sensitivity analysis of the reduction in HOMA-IR duration of treatment not less than 12 weeks among NASH patients. (a) network graph and (b) SUCRA curves 92
Table S61 Network-estimated, weighted mean difference of options for reduction in HOMA-IR duration of treatment not less than 12 weeks among NASH patients. 92

Appendix 1: Search strategies

Table S1 Search algorithms

Database	Search algorithm		Item found
Pubmed	#1	synbiotics	2006
	#2	probiotics	36068
	#3	Lactobacillus*	455324
	#4	Bifidobacterium*	11474
	#5	"Enterococcus faecium"	7172
	#6	"Streptococcus thermophiles"	32
	#7	"Bacillus clausii"	150
	#8	"Saccharomyces cerevisiae"	135887
	#9	“Saccharomyces boulardii”	798
	#10	“Escherichia coli Nissle 1917”	243
	#11	prebiotics	11686
	#12	FOS	34164
	#13	Fructooligosaccharide*	2026
	#14	Fructo-oligosaccharide*	795
	#15	GOS	4829
	#16	Galactooligosaccharide*	1211
	#17	Galacto-oligosaccharide*	710
	#18	XOS	439
	#19	Xylooligosaccharide*	1176
	#20	Xylo-oligosaccharide*	407
	#21	TOS	4536
	#22	Transgalactooligosaccharide*	33
	#23	Trans-galactooligosaccharide*	14
	#24	Inulin	12037
	#25	Lactitol	332
	#26	Lactulose	4251
	#27	Lactosucrose	73
	#28	Soy oligosaccharide*	172
	#29	NAFLD	28193
	#30	NASH	21517
	#31	Fatty liver*	91695
	#32	Non alcoholic fatty liver disease	24306
	#33	Non-alcoholic fatty liver disease	24306
	#34	Nonalcoholic fatty liver disease	30008
	#35	Nonalcoholic fatty liver*	28553
	#36	Nonalcoholic steatohepati*	12617
	#37	#2 OR #3 OR #4 OR #5 OR #6 OR #7 OR #8 OR #9 OR #10	212888
	#38	#11 OR #12 OR #13 OR #14 OR #15 OR #16 OR #17 OR #18 OR #19 OR #20 OR #21 OR #22 OR #23 OR #24 OR #25 OR #26 OR #27 OR #28	70866
	#39	#37 AND #38	7174
	#40	#1 OR #39	7549
	#41	#29 OR #30 OR #31 OR #32 OR #33 OR #34 OR #35 OR #36	106373

Database		Search algorithm	Item found
	#42	#40 AND #41	247
	#43	#37 AND #41	1363
	#44	#38 AND #41	723
	#45	#42 OR #43 OR #44	**1851**
Web of science	#1	synbiotics	1012
	#2	probiotics	20980
	#3	Lactobacillus*	45704
	#4	Bifidobacterium*	10065
	#5	"Enterococcus faecium"	7074
	#6	"Streptococcus thermophiles"	28
	#7	"Bacillus clausii"	164
	#8	"Saccharomyces cerevisiae"	154122
	#9	"Saccharomyces boulardii"	1430
	#10	"Escherichia coli Nissle 1917"	230
	#11	prebiotics	8551
	#12	FOS	41104
	#13	Fructooligosaccharide*	2162
	#14	Fructo-oligosaccharide*	1478
	#15	GOS	4244
	#16	Galactooligosaccharide*	837
	#17	Galacto-oligosaccharide*	1069
	#18	XOS	429
	#19	Xylooligosaccharide*	1071
	#20	Xylo-oligosaccharide*	714
	#21	TOS	3514
	#22	Transgalactooligosaccharide*	20
	#23	Trans-galactooligosaccharide*	17
	#24	Inulin	9367
	#25	Lactitol	456
	#26	Lactulose	4046
	#27	Lactosucrose	95
	#28	Soy oligosaccharide*	277
	#29	NAFLD	12007
	#30	NASH	29926
	#31	Fatty liver*	68564
	#32	Non alcoholic fatty liver disease	9793
	#33	Non-alcoholic fatty liver disease	9654
	#34	Nonalcoholic fatty liver disease	13373
	#35	Nonalcoholic fatty liver*	14054
	#36	Nonalcoholic steatohepati*	11947
	#37	#10 OR #9 OR #8 OR #7 OR #6 OR #5 OR #4 OR #3 OR #2	216781
	#38	#28 OR #27 OR #26 OR #25 OR #24 OR #23 OR #22 OR #21 OR #20 OR #19 OR #18 OR #17 OR #16 OR #15 OR #14 OR #13 OR #12 OR #11	71702
	#39	#38 AND #37	4246
	#40	#39 OR #1	4637
	#41	#36 OR #35 OR #34 OR #33 OR #32 OR #31 OR #30 OR #29	97922

Database		Search algorithm	Item found
	#42	#41 AND #40	92
	#43	#41 AND #37	859
	#44	#38 AND #41	347
	#45	#42 OR #43 OR #44	**1153**
EMBASE	#1	'synbiotics':ti, ab, kw	868
	#2	'probiotics':ti, ab, kw	17, 624
	#3	'Lactobacillus*':ti, ab, kw	31, 164
	#4	'Bifidobacterium*':ti, ab, kw	8, 372
	#5	'Enterococcus faecium':ti, ab, kw	5, 893
	#6	'Streptococcus thermophiles':ti, ab, kw	27
	#7	'Bacillus clausii' :ti, ab, kw	140
	#8	'Saccharomyces cerevisiae':ti, ab, kw	70, 981
	#9	'Saccharomyces boulardii':ti, ab, kw	855
	#10	Escherichia coli Nissle 1917':ti, ab, kw	230
	#11	'prebiotics':ti, ab, kw	5, 753
	#12	'FOS':ti, ab, kw	32, 474
	#13	'Fructooligosaccharide*':ti, ab, kw	1, 189
	#14	'Fructo-oligosaccharide*':ti, ab, kw	79
	#15	'GOS':ti, ab, kw	4, 719
	#16	'Galactooligosaccharide*':ti, ab, kw	585
	#17	'Galacto-oligosaccharide*':ti, ab, kw	682
	#18	'XOS':ti, ab, kw	255
	#19	'Xylooligosaccharide*':ti, ab, kw	659
	#20	'Xylo-oligosaccharide*':ti, ab, kw	324
	#21	'TOS':ti, ab, kw	3, 268
	#22	'Transgalactooligosaccharide*':ti, ab, kw	13
	#23	'Trans-galactooligosaccharide*':ti, ab, kw	14
	#24	'Inulin':ti, ab, kw	9, 324
	#25	'Lactitol':ti, ab, kw	377
	#26	'Lactulose':ti, ab, kw	5, 192
	#27	'Lactosucrose':ti, ab, kw	71
	#28	'Soy oligosaccharide*':ti, ab, kw	12
	#29	'NAFLD':ti, ab, kw	20, 334
	#30	'NASH':ti, ab, kw	13, 786
	#31	'Fatty liver*':ti, ab, kw	37, 760
	#32	'Non alcoholic fatty liver disease':ti, ab, kw	12, 940
	#33	'Non-alcoholic fatty liver disease':ti, ab, kw	12, 789
	#34	'Nonalcoholic fatty liver disease':ti, ab, kw	11, 890
	#35	'Nonalcoholic fatty liver*':ti, ab, kw	12, 391
	#36	'Nonalcoholic steatohepati*':ti, ab, kw	6, 883
	#37	#2 OR #3 OR #4 OR #5 OR #6 OR #7 OR #8 OR #9 OR #10	120, 090
	#38	#11 OR #12 OR #13 OR #14 OR #15 OR #16 OR #17 OR #18 OR #19 OR #20 OR #21 OR #22 OR #23 OR #24 OR #25 OR #26 OR #27 OR #28	60, 856
	#39	#37 AND #38	2, 833
	#40	#1 OR #39	3, 179
	#41	#29 OR #30 OR #31 OR #32 OR #33 OR #34 OR #35 OR #36	47, 513

Database	Search algorithm		Item found
	#42	#40 AND #41	50
	#43	#37 AND #41	283
	#44	#38 AND #41	186
	#45	#42 OR #43 OR #44	**437**
CINAHL Plus	#1	synbiotics	266
	#2	probiotics	6, 898
	#3	Lactobacillus*	2, 886
	#4	Bifidobacterium*	1, 216
	#5	"Enterococcus faecium"	574
	#6	"Streptococcus thermophiles"	6
	#7	"Bacillus clausii"	5
	#8	"Saccharomyces cerevisiae"	293
	#9	"Saccharomyces boulardii"	96
	#10	"Escherichia coli Nissle 1917"	14
	#11	prebiotics	1, 692
	#12	FOS	1, 000
	#13	Fructooligosaccharide*	112
	#14	Fructo-oligosaccharide*	112
	#15	GOS	490
	#16	Galactooligosaccharide*	52
	#17	Galacto-oligosaccharide*	77
	#18	XOS	9
	#19	Xylooligosaccharide*	16
	#20	Xylo-oligosaccharide*	5
	#21	TOS	382
	#22	Transgalactooligosaccharide*	1
	#23	Trans-galactooligosaccharide*	2
	#24	Inulin	443
	#25	Lactitol	18
	#26	Lactulose	430
	#27	Lactosucrose	2
	#28	Soy oligosaccharide*	22
	#29	NAFLD	1, 847
	#30	NASH	876
	#31	Fatty liver*	7, 559
	#32	Non alcoholic fatty liver disease	1, 072
	#33	Non-alcoholic fatty liver disease	1062
	#34	Nonalcoholic fatty liver disease	2, 639
	#35	Nonalcoholic fatty liver*	2, 780
	#36	Nonalcoholic steatohepati*	873
	#37	#2 OR #3 OR #4 OR #5 OR #6 OR #7 OR #8 OR #9 OR #10	9, 298
	#38	#11 OR #12 OR #13 OR #14 OR #15 OR #16 OR #17 OR #18 OR #19 OR #20 OR #21 OR #22 OR #23 OR #24 OR #25 OR #26 OR #27 OR #28	4, 290
	#39	#37 AND #38	1, 175
	#40	#1 OR #39	1, 270
	#41	#29 OR #30 OR #31 OR #32 OR #33 OR #34 OR #35 OR #36	7, 961

Database	Search algorithm		Item found
	#42	#40 AND #41	27
	#43	#37 AND #41	128
	#44	#38 AND #41	63
	#45	#42 OR #43 OR #44	**167**
Cochrane Library	#1	synbiotics	356
	#2	probiotics	3509
	#3	Lactobacillus*	3283
	#4	Bifidobacterium*	1636
	#5	"Enterococcus faecium"	127
	#6	"Streptococcus thermophiles"	13
	#7	"Bacillus clausii"	21
	#8	"Saccharomyces cerevisiae"	160
	#9	"Saccharomyces boulardii"	211
	#10	"Escherichia coli Nissle 1917"	32
	#11	prebiotics	795
	#12	FOS	375
	#13	Fructooligosaccharide*	347
	#14	Fructo-oligosaccharide*	347
	#15	GOS	574
	#16	Galactooligosaccharide*	240
	#17	Galacto-oligosaccharide*	240
	#18	XOS	16
	#19	Xylooligosaccharide*	22
	#20	Xylo-oligosaccharide*	22
	#21	TOS	469
	#22	Transgalactooligosaccharide*	6
	#23	Trans-galactooligosaccharide*	6
	#24	Inulin	777
	#25	Lactitol	128
	#26	Lactulose	1223
	#27	Lactosucrose	2
	#28	Soy oligosaccharide*	22
	#29	NAFLD	1011
	#30	NASH	1282
	#31	Fatty liver*	3279
	#32	Non alcoholic fatty liver disease	1126
	#33	Non-alcoholic fatty liver disease	1093
	#34	Nonalcoholic fatty liver disease	1486
	#35	Nonalcoholic fatty liver*	1650
	#36	Nonalcoholic steatohepati*	675
	#37	#10 OR #9 OR #8 OR #7 OR #6 OR #5 OR #4 OR #3 OR #2	5565
	#38	#28 OR #27 OR #26 OR #25 OR #24 OR #23 OR #22 OR #21 OR #20 OR #19 OR #18 OR #17 OR #16 OR #15 OR #14 OR #13 OR #12 OR #11	3957
	#39	#38 AND #37	747
	#40	#39 OR #1	893
	#41	#36 OR #35 OR #34 OR #33 OR #32 OR #31 OR #30 OR #29	2472

Database	Search algorithm		Item found
	#42	#41 AND #40	27
	#43	#41 AND #37	62
	#44	#38 AND #41	37
	#45	#42 OR #43 OR #44	**256**

Appendix 2: General characteristics of interventions

Table S2 General characteristics of interventions

Intervention	General characteristics
Prebiotics	Fructooligosaccharide, Inulin, Oligofructose
Probiotics	*Lactobacillus* spp., *Bifidobacterium* spp., *Streptococcus thermophiles*, *Pediococcuspentosaceus*
Synbiotics	The composition of prebiotics and probiotics

Appendix 3: Description of interventions of each included study

Table S3 Description of interventions

ID	Author, year	Population	Intervention	Detail of intervention	N	Treatment duration
1	Aller R, 2011	Adults, NAFLD	Probiotics	500 million of *Lactobacillus bulgaricus* and *Streptococcus thermophilus*<br>1 tablet/day	14	12 weeks
			Placebo	Indistinguishable placebo	14	
2	Vajro P, 2011	Children, NAFLD	Probiotics	Oral *Lactobacillus rhamnosus* Strain GG<br>12 billion CFU/day	10	8 weeks
			Placebo	Indistinguishable placebo	10	
3	Malaguarnera M, 2012	Adults, NASH	Synbiotics	*Bifidobacterium longum* and FOS 2.5 g<br>Vitamin B1 1.4 mg, Vitamin B2 1.6 mg, Vitamin B6 2.0 mg, and Vitamin B12 1.0 mg<br>1 tablet/day	34	24 weeks
			Placebo	Vitamin B1 1.4 mg, Vitamin B2 1.6 mg, Vitamin B6 2.0 mg, and Vitamin B12 1.0 mg	32	
4	Wong VW, 2013	Adults, NASH	Probiotics	*Lactobacillus plantarum, Lactobacillus delbrueckii* ssp. *bulgaricus, Lactobacillus acidophilus, Lactobacillus rhamnosus,* and *Bifidobacterium bifidum*<br>10 g/day	10	24 weeks
			Placebo	Indistinguishable placebo	10	
5	Alisi A, 2014	Children, NAFLD	Probiotics	Eight probiotics strains *(Streptococcus thermophilus, Bifidobacteria [B. breve, B. infantis,* and *B. longum], Lactobacillus acidophilus [L. plantarum, L. paracasei,* and *L. delbrueckii subsp. bulgaricus)*<br>1 sachet/day or 2 sachet/day for >10 years old children	22	16 weeks
			Placebo	Indistinguishable placebo	22	
6	Eslamparast T, 2014	Adults, NAFLD	Synbiotics	Synbiotics capsule (Protexin) contained 200 million of 7 strains of bacteria *[Lactobacillus casei, Lactobacillus rhamnosus, Streptococcus thermophilus, Bifidobacterium breve, Lactobacillus acidophilus, Bifidobacterium longum,* and *Lactobacillus bulgaricus]*<br>+ Prebiotics [fructooligosaccharide] + Probiotics cultures [magnesium stearate (source: mineral and vegetable) and a vegetable capsule (hydroxypropyl methyl cellulose)].	26	28 weeks
			Placebo	Indistinguishable placebo	26	

ID	Author, year	Population	Intervention	Detail of intervention	N	Treatment duration
7	Miccheli A, 2015	Children, NAFLD	Probiotics	VSL#3 1 sacchet/day or 2 sacchet/day for >10 years old children	15	16 weeks
			Placebo	Indistinguishable placebo	16	
8	Spideh A, 2015	Adults, NAFLD	Probiotics	*Lactobacillus casei, Lactobacillus acidophilus, Lactobacillus rhamnosus, Lactobacillus bulgaricus, Bifidobacterium breve, Bifidobacterium longum,* and *Streptococcus thermophilus*<br>1 capsule/day	21	8 weeks
			Placebo	Indistinguishable placebo	21	
9	Akbarzadeh Z, 2016	Adults, NAFLD	Prebiotics	10 g psyllium<br>1 capsule/day	38	10 weeks
			Placebo	10 g crashed wheat	37	
10	Asgharian A, 2016	Adults, NAFLD	Synbiotics	500 mg capsule containing 7 species of bacteria *(Lactobacillus casei, Lactobacillus acidopholus, Lactobacillus rhamnosus, Lactobacillus bulgaricus, Bifidobacteriumbreve, Bifidobacteriumlongum,* and *Streptococcus thermophiles),* and Prebiotics (fructooligosaccharides)<br>1 capsule/day	38	8 weeks
			Placebo	Placebo capsule containing 120 mg starch, similar in shape and appearance	36	
11	Ekhlasi G, 2016	Adults, NAFLD	Synbiotic	Each symbiotic capsule contained *Lactobacillus casei, Lactobacillus rhamnosus, Streptococcus thermophilus, Bifidobacterium breve, Lactobacillus acidophilus, Bifidobacterium longum, Lactobacillus bulgaricus*, and prebiotic (fructooligosaccharide). The concentration of each probiotic strain was 2×10^8 CFU/g per capsule. Synbiotic supplements were administered as 2 capsules per day orally after the main meal.	15	8 weeks
			Placebo	Two identical-appearing placebo capsules (corn starch, Zahravi Pharmaceutical Co.,) were taken daily by participants assigned to placebo group.	15	
12	Ferolla SM, 2016	Adults, NASH	Synbiotics	5 g of the synbiotics which consisted of 4 g of dietary fiber (partially hydrolyzed guar gum and inulin) and $1\text{x}10^8$ CFU of *L. reuteri*<br>1 capsule twice daily	27	12 weeks
			Placebo	Indistinguishable placebo	23	
13	Asgharian A, 2017	Adults, NAFLD	Synbiotics	500 mg capsule containing 7 species of probiotics bacteria *(Lactobacillus casei, Lactobacillus acidopholus, Lactobacillus rhamnosus, Lactobacillus bulgaricus, Bifidobacteriumbreve, Bifidobacteriumlongum, Streptococcus thermophiles)* and fructooligosaccharides<br>1 capsule/day	38	8 weeks

ID	Author, year	Population	Intervention	Detail of intervention	N	Treatment duration
			Placebo	A placebo capsule containing 120 mg starch similar in shape and appearance	36	
14	Behrouz V, 2017	Adults, NAFLD	Probiotics	Probiotics capsule contained 5 billion of 5 bacterial strains *(Lactobacillus casei, Lactobacillus rhamnosus, Lactobacillus acidophilus, Bifidobacterium longum, and Bifidobacterium breve)* plus 8 gr prebiotics placebo (maltodextrin)<br>1 probiotic capsule +1 prebiotics placebo twice daily	30	12 weeks
			Prebiotics	Oligofructose plus 8 gr probiotics placebo (maltodextrin)<br>1 prebiotic capsule + 1 probiotics placebo twice daily	29	
			Placebo	2 capsule of placebo (maltodextrin) twice daily	30	
15	Famouri F, 2017	Children, NAFLD	Probiotics	*Lactobacillus acidophilus* $3x10^9$ CFU, *Bifidobacterium lactis* $6x10^9$ CFU, *Bifidobacterium bifidum* $2x10^9$ CFU and *Lactobacillus rhamnosus* $2x10^9$ CFU<br>1 capsule/day	32	12 weeks
			Placebo	Indistinguishable placebo	32	
16	Javadi L, 2017 (1)	Adults, NAFLD	Synbiotics	*Bifidobacterium longum* and *Lactobacillus acidophilus* $2x10^7$ capsule 250 mg/day plus inulin 10 g/day twice daily	17	12 weeks
			Probiotics	*Bifidobacterium longum* and *Lactobacillus acidophilus* $2x10^7$ capsule 250 mg/day plus maltodextrin powder 5 g twice daily	20	
			Prebiotics	Inulin powder 10 g/day plus fat and lactose free milk capsule twice daily	19	
			Placebo	Maltodextrin powder plus fat and lactose free milk capsule twice daily	19	
17	Javadi L, 2017 (2)	Adults, NAFLD	Synbiotics	*Bifidobacterium longum* and *Lactobacillus acidophilus* $2x10^7$ capsule 250 mg/day plus inulin 10 g/day twice daily	17	12 weeks
			Probiotics	*Bifidobacterium longum* and *Lactobacillus acidophilus* $2x10^7$ capsule 250 mg/day plus maltodextrin powder 5 g twice daily	20	
			Prebiotics	Inulin powder 10 g/day plus fat and lactose free milk capsule twice daily	19	
			Placebo	Maltodextrin powder plus fat and lactose free milk capsule twice daily	19	
18	Manzhalii E, 2017	Adults, NASH	Synbiotics	Lactiale® *containing Lactobacillus casei, L. rhamnosus, L. bulgaris, Bifidobacterium longum, and Streptococcus thermophilus* (108 bacteria/capsule in total) and fructooligosaccharides<br>1 capsule/day	38	12 weeks
			Placebo	Indistinguishable placebo	37	

ID	Author, year	Population	Intervention	Detail of intervention	N	Treatment duration
19	Mofidi F, 2017	Adults, NAFLD	Synbiotics	Synbiotics capsule contained 200 million bacteria of seven strains (*Lactobacillus casei, Lactobacillus rhamnosus, Streptococcus thermophilus, Bifidobacterium breve, Lactobacillus acidophilus, Bifidobacterium longum,*and *Lactobacillus bulgaricus*) and prebiotics (125 mg fructooligosaccharide) and probiotics cultures (magnesium stearate (source: mineral and vegetable) and a vegetable capsule (hydroxypropylmethyl cellulose))	21	28 weeks
			Placebo	Maltodexterin	21	
20	Monem SMA, 2017	Adults, NASH	Probiotics	Acidophilus capsule; *Lactobacillus acidophilus* 2 billion viable organism<br>1 tablet 3 times/day 30 minutes before meal	15	4 weeks
			Placebo	Mixture of rice flour, gelatin, and magnesium stearate in tablet	15	
21	Bakhshimoghadd am F, 2018	Adults, NAFLD	Synbiotics	The synbiotic yogurt contained 10^8 CFUs *Streptococcus thermophilus* and *Lactobacillus delbrueckii*subsp. *Bulgaricus, Bifidobacterium animalis* subsp. lactis (BB-12)/mL as a probiotic and 1.5 g inulin as a prebiotic.	34	24 weeks
			Placebo	The yogurts were specially prepared for this study by the West AzarbaijanPegah Dairy Company (Urmia, Iran).	34	
22	Ahn SB, 2019	Adults, NAFLD	Probiotic	A probiotic mixture of *L. acidophilus* CBT LA1, *L. rhamnosus* CBT LR5 isolated from Korean human feces, *L. paracasei* CBT LPC5 isolated from Korean fermented food (jeotgal), *P. pentosaceus* CBT SL4 isolated from a Korean fermented vegetable product (kimchi), *B. lactis* CBT BL3, and *B. breve* CBT BR3 isolated from Korean infant feces was used. The probiotic mixture contained 109 CFU/1.4 g.	30	12 weeks
			Placebo	The placebo was composed of dextran, maltodextrin, lemon flavor, and Mg stearate.	35	
23	Duseja A, 2019	Adults, NAFLD	Probiotic	Two capsules t.i.d. (Each capsule containing 112.5 billion live, lyophilised, lactic acid bacteria and *bifidobacteria*, namely *Lactobacillus paracasei* DSM 24733, *Lactobacillus plantarum* DSM 24730, *Lactobacillus acidophilus* DSM 24735, and *Lactobacillus delbrueckii* subsp. *bulgaricus* DSM 24734, *Bifidobacterium longum* DSM 24736, *Bifidobacterium infantis* DSM 24737, *Bifidobacterium breve* DSM 24732, and *Streptococcus thermophilus* DSM 24731.	17	48 weeks
			Placebo	Two placebo capsules t.i.d. (capsules containing microcrystalline cellulose).	13	
24	Abhari K, 2020	Adults, NAFLD	Synbiotic	One synbiotic capsulecontaining 109 spore of *B. coagulans* (GBI-30) plus 0.4 g inulin perday.	22	12 weeks
			Placebo	Maltodextrin	24	

ID	Author, year	Population	Intervention	Detail of intervention	N	Treatment duration
25	Behrouz V, 2020	Adults, NAFLD	Probiotic	Each probiotic capsule contained 5 billion of five bacterial strains (*Lactobacillus casei, Lactobacillus rhamnosus, Lactobacillus acidophilus, Bifidobacterium longum,* and *Bifidobacterium breve*).	30	12 weeks
			Prebiotic	Oligofructose	29	
			Placebo	Maltodextrin	30	
26	Chong PL, 2021	Adults, NAFLD	Probiotic	VSL#3® (Danisco Inc.) contained 8 different strains of live freeze-dried lactic acid bacteria (*Streptococcus thermophilus, Bifidobacterium breve, Bifidobacterium longum, Bifidobacterium infantis, Lactobacillus acidophilus, Lactobacillus plantarum, Lactobacillus paracasei, and Lactobacillus bulgaricus*).	19	10 weeks
			Placebo	Placebo	16	

FOS, fructooligosaccharide; g, gram; mg, milligram; N, number of patients; NAFL, non-alcoholic fatty liver; NASH, non-alcoholic steatohepatitis

Appendix 4: Baseline characteristics of included studies

Table S4 Baseline characteristics of patients with non-alcoholic fatty liver disease

ID	Author, year	Population	Intervention	Age (years)	AST (IU/L)	ALT (IU/L)	BMI (kg/m^2)	WC (cm)	TC (mg/dL)	TG (mg/dL)	LDL (mg/dL)	HDL (mg/dL)	FBS (mg/dL)	HOMA-IR
				Mean (SD)	Mean (SD)	Mean (SD)	Mean (SD)	Mean (SD)	Mean (SD)	Mean (SD)	Mean (SD)	Mean (SD)	Mean (SD)	Mean (SD)
1	Aller R, 2011	Adults, NAFLD	Probiotics	49.4 (10.9)	41.3 (15.5)	67.7 (25.1)	30.2 (4.5)	-	194.8 (49.1)	171.1 (95.4)	110.3 (39.9)	43.0 (11.9)	116.0 (25.4)	4.5 (2.6)
			Placebo	44.3 (15.1)	31.7 (13.1)	60.7 (32.1)	29.5 (5.5)	-	192.7 (38.6)	134.8 (51.8)	125.6 (32.3)	40.1 (6.7)	110.0 (28.5)	4.2 (3.2)
2	Vajro P, 2011	Children, NAFLD	Probiotics	10.7 (2.1)	41.6 (16.5)	70.3 (34.8)	-	-	157.0 (29.9)	96.9 (9.7)	-	-	-	-
			Placebo			63.6 (18.5)	-	-			-	-	-	-
3	Malaguarnera M, 2012	Adults, NASH	Synbiotics	46.9 (5.4)	109.0 (23.2)	101.0 (24.7)	27.3 (1.36)	93.2 (5.2)	210.4 (30.9)	212.6 (71.7)	151.2 (29.8)	40.6 (29.8)	107.2 (13.3)	3.7 (0.5)
			Placebo	46.7 (5.7)	107.1 (21.4)	96.1 (24.2)	27.2 (1.32)	93.4 (5.1)	207.3 (30.6)	209.0 (77.1)	147.3 (30.2)	41.8 (30.2)	107.9 (12.1)	3.7 (0.5)
4	Wong VW, 2013	Adults, NASH	Probiotics	42.0 (9.0)	50.0 (25.0)	96.0 (75.0)	30.2 (5.0)	101.0 (10.0)	185.6 (30.9)	209.7 (129.5)	100.5 (23.2)	46.4 (7.7)	109.9 (25.2)	-
			Placebo	55.0 (9.0)	38.0 (15.0)	72.0 (30.0)	28.7 (5.7)	97.0 (14.0)	193.4 (38.7)	169.3 (99.0)	108.3 (34.8)	50.3 (15.5)	157.3 (166.7)	-
5	Alisi A, 2014	Children, NAFLD	Probiotics	10.4 (2.4)	59.2 (15.1)	34.0 (4.7)	27.1 (0.1)	-	156.0 (30.1)	99.0 (18.8)	85.2 (23.8)	45.4 (11.9)	84.7 (9.5)	4.3 (1.4)
			Placebo	11.0 (1.6)	63.4 (16.6)	42.0 (4.7)	25.6 (0.1)	-	156.7 (34.9)	98.0 (14.1)	94.5 (24.6)	48.0 (9.5)	83.9 (8.7)	4.7 (1.9)
6	Eslamparast T, 2014	Adults, NAFLD	Synbiotics	46.4 (8.8)	66.4 (2.6)	69.3 (2.3)	32.1 (2.4)	102.4 (6.8)	-	-	-	-	99.6 (24.2)	2.8 (1.0)
			Placebo	45.7 (9.5)	68.3 (9.4)	71.5 (9.1)	31.3 (2.3)	102.8 (6.2)	-	-	-	-	98.9 (21.4)	2.7 (1.2)
7	Miccheli A, 2015	Children, NAFLD	Probiotics	10.4 (2.5)	62.6 (12.9)	24.9 (9.0)	26.6 (2.9)	-	165.4 (36.2)	86.0 (42.1)	87.7 (31.5)	45.5 (13.1)	82.6 (7.5)	3.6 (1.8)

ID	Author, year	Population	Intervention	Age (years)	AST (IU/L)	ALT (IU/L)	BMI (kg/m^2)	WC (cm)	TC (mg/dL)	TG (mg/dL)	LDL (mg/dL)	HDL (mg/dL)	FBS (mg/dL)	HOMA-IR
				Mean (SD)	Mean (SD)	Mean (SD)	Mean (SD)	Mean (SD)	Mean (SD)	Mean (SD)	Mean (SD)	Mean (SD)	Mean (SD)	Mean (SD)
			Placebo	11.0 (1.6)	64 .0 (16.3)	34.3 (14.6)	25.4 (4.3)	-	165.6 (40.6)	87.0 (40.6)	100.0 (31.7)	47.7 (8.1)	83.7 (9.8)	4.2 (3.0)
8	Sepideh A, 2015	Adults, NAFLD	Probiotics	42.1 (2.0)	-	-	30.3 (1.2)	98.1 (2.4)	-	-	-	-	98.0 (3.7)	2.7 (0.4)
			Placebo	47.3 (2.5)	-	-	29.5 (0.8)	99.6 (2.4)	-	-	-	-	97.8 (3.9)	3.0 (0.3)
9	Akbarzadeh Z, 2016	Adults, NAFLD	Prebiotics	46.1 (17.1)	35.7 (19.9)	46.7 (29.1)	29.4 (3.4)	-	-	-	-	-	-	-
			Placebo	43.8 (11.7)	46.3 (51.2)	68.1 (76.8)	31.1 (3.6)	-	-	-	-	-	-	-
10	Asgharian A, 2016	Adults, NAFLD	Synbiotics	46.6 (10.5)	25.3 (22.6)	24.6 (13.3)	29.6 (4.7)	90.8 (10.7)	-	-	-	-	-	-
			Placebo	47.8 (10.2)	24.1 (11.2)	26.3 (16.9)	28.2 (4.1)	88.9 (9.4)	-	-	-	-	-	-
11	Ekhlasi G, 2016	Adults, NAFLD	Synbiotics	44.5 (6.7)	38.0 (15.3)	38.1 (8.7)	27.3 (2.2)	93.6 (6.5)	194.3 (24.2)	187.5 (20.6)	140.0 (40.5)	46.1 (30.3)	115.7 (9.9)	0.6 (0.2)
			Placebo		32.0 (7.1)	33.88 (4.5)	27.8 (2.0)	96.5 (7.3)	194.4 (15.9)	182.8 (30.2)	138.3 (15.1)	38.2 (8.3)	108.0 (14.5)	0.6 (0.2)
12	Ferolla SM, 2016	Adults, NASH	Synbiotics	51.8 (37.4)	81.5 (142.5)	61.5 (93.1)	32.5 (4.0)	107.8 (10.8)	205.4 (34.6)	201.3 (178.5)	125.2 (28.0)	44.9 (10.3)	158.0 (154.1)	-
			Placebo		52.8 (117.9)	61.6 (104.1)	32.5 (4.0)	104.0 (13.6)	190.9 (42.5)	275.3 (427.5)	109.3 (32.7)	46.7 (11.7)	161.7 (154.08)	-
13	Asgharian A, 2017	Adults, NAFLD	Synbiotics	46.6 (10.5)	-	-	29.6 (4.7)	90.8 (10.7)	203.1 (8.0)	162.6 (11.9)	121.6 (6.8)	45.4 (1.9)	97.5 (1.5)	-
			Placebo	47.8 (10.2)	-	-	28.2 (4.1)	88.9 (9.4)	187.4 (6.6)	174.7 (20.5)	104.7 (6.1)	45.3 (1.8)	101.5 (2.4)	-
14	Behrouz V, 2017	Adults, NAFLD	Probiotics	38.5 (7.1)	-	-	29.6 (2.5)	-	-	-	-	-	91.5 (9.9)	3.8 (2.1)
			Prebiotics	38.4 (9.2)	-	-	30.8 (4.7)	-	-	-	-	-	95.3 (17.7)	3.6 (1.9)

ID	Author, year	Population	Intervention	Age (years)	AST (IU/L)	ALT (IU/L)	BMI (kg/m^2)	WC (cm)	TC (mg/dL)	TG (mg/dL)	LDL (mg/dL)	HDL (mg/dL)	FBS (mg/dL)	HOMA-IR
				Mean (SD)	Mean (SD)	Mean (SD)	Mean (SD)	Mean (SD)	Mean (SD)	Mean (SD)	Mean (SD)	Mean (SD)	Mean (SD)	Mean (SD)
			Placebo	38.4 (10.1)	-	-	31.9 (5.0)	-	-	-	-	-	98.3 (24.2)	4.7 (5.9)
15	Famouri F, 2017	Adults, NAFLD	Probiotics	12.7 (2.2)	32.2 (15.7)	32.8 (19.6)	26.4 (4.3)	82.2 (14.7)	157.3 (57.1)	112.5 (50.5)	87.9 (28.7)	46.3 (12.1)	-	-
			Placebo	12.6 (1.7)	30.2 (12.9)	28.7 (13.7)	26.6 (2.3)	81.4 (6.8)	108.0 (24.3)	96.0 (20.7)	79.3 (14.2)	34.5 (4.6)	-	-
16	Javadi L, 2017 (1)	Adults, NAFLD	Synbiotics	43.2 (7.0)	51.6 (10.9)	58.1 (13.8)	32.3 (4.8)	-	-	-	-	-	-	-
			Probiotics	43.9 (9.0)	45.9 (14.5)	51.2 (13.6)	29.9 (3.9)	-	-	-	-	-	-	-
			Prebiotics	38.7 (10.0)	42.7 (10.0)	49.1 (11.8)	31.0 (4.4)	-	-	-	-	-	-	-
			Placebo	42.2 (9.1)	43.6 (13.2)	50.2 (11.0)	30.4 (2.9)	-	-	-	-	-	-	-
17	Javadi L, 2017 (2)	Adults, NAFLD	Synbiotics	43.2 (7.0)	-	-	32.3 (4.8)	-	204.4 (32.8)	190.6 (66.4)	121.0 (27.9)	42.3 (12.0)	101.8 (5.4)	1.5 (0.7)
			Probiotics	43.9 (9.0)	-	-	29.9 (3.9)	-	194.2 (32.5)	165.9 (54.2)	118.5 (37.5)	42.0 (8.3)	102.6 (7.8)	1.2 (0.4)
			Prebiotics	38.7 (10.0)	-	-	31.0 (4.4)	-	189.5 (29.1)	172.1 (73.1)	115.0 (24.9)	40.3 (1.3)	100.2 (9.6)	1.1 (0.5)
			Placebo	42.2 (9.1)	-	-	30.4 (2.9)	-	188.9 (23.8)	150.1 (45.7)	114.6 (22.6)	38.3 (8.0)	101.1 (6.4)	1.1 (0.4)
18	Manzhalii E, 2017	Adults, NASH	Synbiotics	44.3 (9.3)	43.1 (12.9)	56.8 (17.3)	26.4 (4.9)	-	282.3 (47.7)	129.3 (114.7)	-	51.8 (95.4)	88.7 (65.0)	-
			Placebo	43.5 (7.9)	41.9 (11.6)	52.4 (17.6)	26.6 (4.3)	-	274.6 (47.7)	122.3 (97.0)	-	53.0 (89.4)	90.4 (60.2)	-
19	Mofidi, 2017	Adults, NAFLD	Synbiotics	40.1 (11.4)	63.6 (2.8)	72.3 (5.1)	23.2 (1.0)	92.0 (4.2)	-	-	-	-	-	-
			Placebo	44.6 (10.1)	72.7 (2.6)	81.3 (3.7)	23.2 (1.1)	89.4 (6.1)	-	-	-	-	-	-

ID	Author, year	Population	Intervention	Age (years)	AST (IU/L)	ALT (IU/L)	BMI (kg/m^2)	WC (cm)	TC (mg/dL)	TG (mg/dL)	LDL (mg/dL)	HDL (mg/dL)	FBS (mg/dL)	HOMA-IR
				Mean (SD)	Mean (SD)	Mean (SD)	Mean (SD)	Mean (SD)	Mean (SD)	Mean (SD)	Mean (SD)	Mean (SD)	Mean (SD)	Mean (SD)
20	Monem SMA, 2017	Adults, NASH	Probiotics	44.2 (5.5)	44.05 (14.65)	81.45 (23.32)	32.6 (1.2)	-	258.6 (31.7)	257.8 (55.02)	158.5 (23.7)	48.7 (4.8)	104.9 (21.3)	-
			Placebo	44.3 (5.6)	42.73 (9.95)	83.53 (12.01)	33.1 (1.3)	-	250.5 (45.1)	245.3 (70.8)	154.7 (33.2)	48.8 (5.0)	107.1 (27.1)	-
21	Bakhshimogadam F, 2018	Adults, NAFLD	Synbiotics	38.8 (9.0)	24.6 (6.6)	34.8 (17.2)	-	-	195.3 (34.7)	165.7 (60.9)	113.8 (25.5)	49.2 (11.0)	89.8 (7.9)	3.7 (1.1)
			Placebo	41.1 (8.5)	22.9 (8.1)	32.4 (17.1)	-	-	195.0 (40.5)	155.7 (53.8)	113.9 (27.9)	47.5 (10.4)	89.8 (8.5)	4.7 (1.6)
22	Ahn SB, 2019	Adults, NAFLD	Probiotic	41.7 (12.5)	33.6 (21.0)	41.10 (24.52)	30.1 (22.4)	-	201.3 (33.8)	187.4 (118.3)	-	46.6 (33.5)	112.7 (72.0)	93.2 (36.7)
			Placebo	44.7 (13.3)	32.0 (17.8)	41.70 (21.64)	30.1 (22.5)	-	196.7 (40.8)	172.8 (108.2)	-	47.4 (37.1)	100.0 (71.9)	56.2 (35.9)
23	Duseja A, 2019	Adults, NAFLD	Probiotic	38.0 (10.0)	68.0 (32.3)	101.1 (48.0)	-	-	180.0 (49.0)	205.0 (137.0)	102.0 (38.0)	40.0 (9.0)	98.0 (15.0)	-
			Placebo	33.0 (6.0)	74.2 (30.1)	105.5 (51.0)	-	-	172.0 (43.0)	146.0 (58.0)	107.0 (37.0)	40.0 (7.0)	96.0 (16.0)	-
24	Abhari K, 2020	Adults, NAFLD	Synbiotics	47.7 (11.4)	40.3 (20.5)	41.9 (18.1)	32.3 (6.7)	103.8 (10.9)	224.9 (49.6)	161.7 (54.6)	149.3 (47.9)	39.5 (7.4)	105.5 (17.7)	3.5 (1.3)
			Placebo	46.7 (12.4)	33.3 (12.4)	36.7 (18.8)	35.5 (5.3)	106.9 (8.8)	211.0 (31.8)	168.9 (57.9)	137.0 (33.3)	40.0 (3.3)	104.4 (23.4)	3.8 (1.5)
25	Behrouz V, 2020	Adults, NAFLD	Probiotic	38.5 (7.1)	74.4 (15.8)	72.5 (11.7)	29.3 (3.2)	102.5 (9.3)	161.1 (30.7)	213.2 (54.4)	91.2 (22.7)	40.3 (10.1)	89.8 (10.1)	-
			Prebiotic	38.4 (9.2)	74.1 (16.7)	76.5 (22.2)	30.0 (4.6)	108.3 (9.7)	165.9 (36.6)	221.5 (78.2)	94.4 (32.1)	40.5 (7.4)	93.0 (14.8)	-
			Placebo	38.4 (10.1)	66.3 (13.0)	63.2 (14.4)	31.6 (7.3)	108.6 (10.7)	156.3 (27.5)	201.1 (66.1)	88.3 (24.8)	39.3 (8.6)	94.5 (15.2)	-
26	Chong PL, 2021	Adults, NAFLD	Probiotic	57.0 (8.0)	40.0 (16.0)	43.0 (44.9)	31.2 (7.3)	112.2 (14.3)	174.4 (53.4)	167.4 (50.5)	99.8 (45.6)	41.4 (10.1)	-	2.2 (1.9)
			Placebo	58.0 (7.0)	40.0 (15.0)	51.0 (19.0)	31.9 (3.3)	111.2 (10.7)	166.7 (32.9)	186.9 (101.8)	93.6 (27.1)	40.6 (13.2)	-	3.1 (1.8)

ALT, alanine aminotransferase; AST, aspartate aminotransferase; BMI, body mass index; FBS, fasting blood sugar; HDL, high- density lipoproteins; HOMA-IR, homeostatic model assessment-insulin resistance; LDL, low- density lipoproteins; NAFL, non-alcoholic fatty liver; NASH, non-alcoholic steatohepatitis; RCT, randomized controlled trial; TC, total cholesterol; TG, triglycerides; WC, waist circumference

Appendix 5: Networks of all option comparisons for secondary outcomes

The size of the nodes is proportional to the number of participants randomized for each option. The directly compared options are linked with the line, the thickness of which corresponds to the number of trials comparing each option.

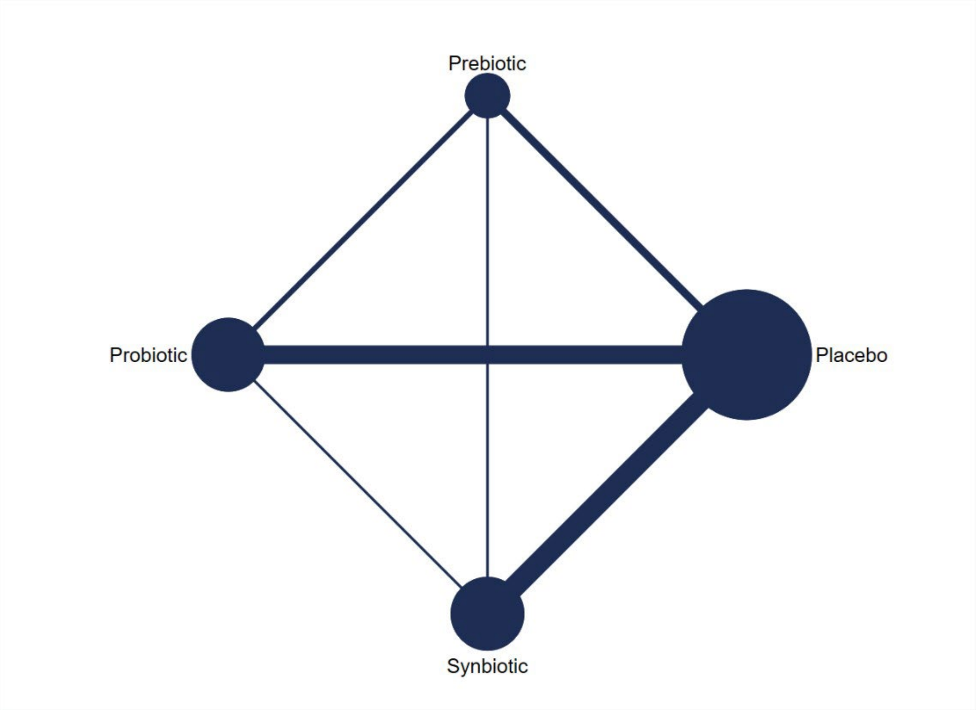

Figure S1 Networks of all option comparisons for reduction in body mass index

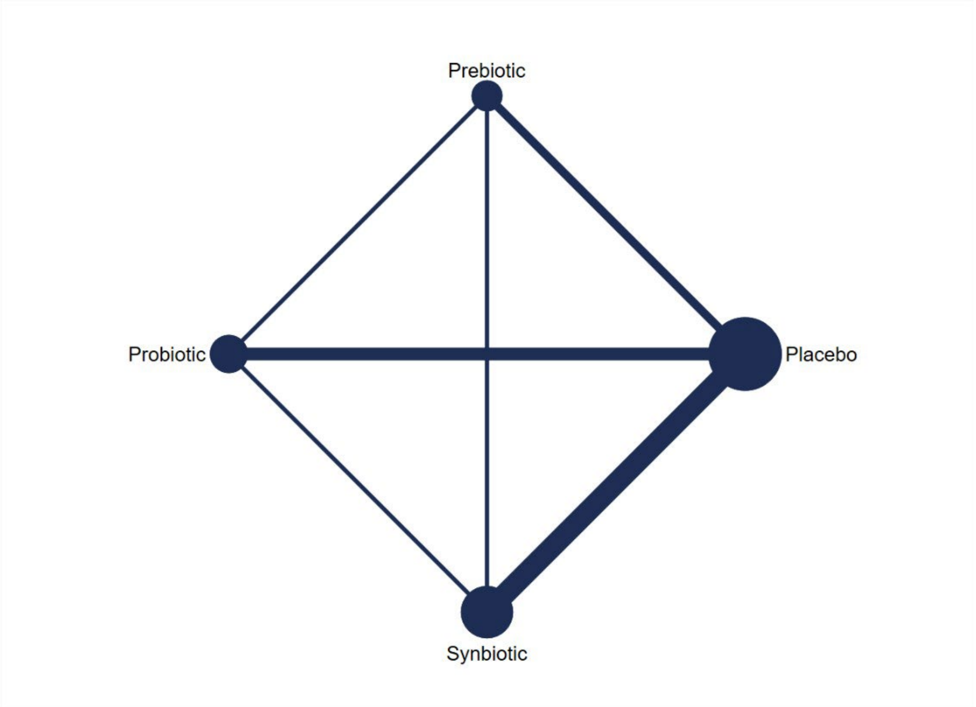

Figure S2 Networks of all option comparisons for reduction in waist circumference

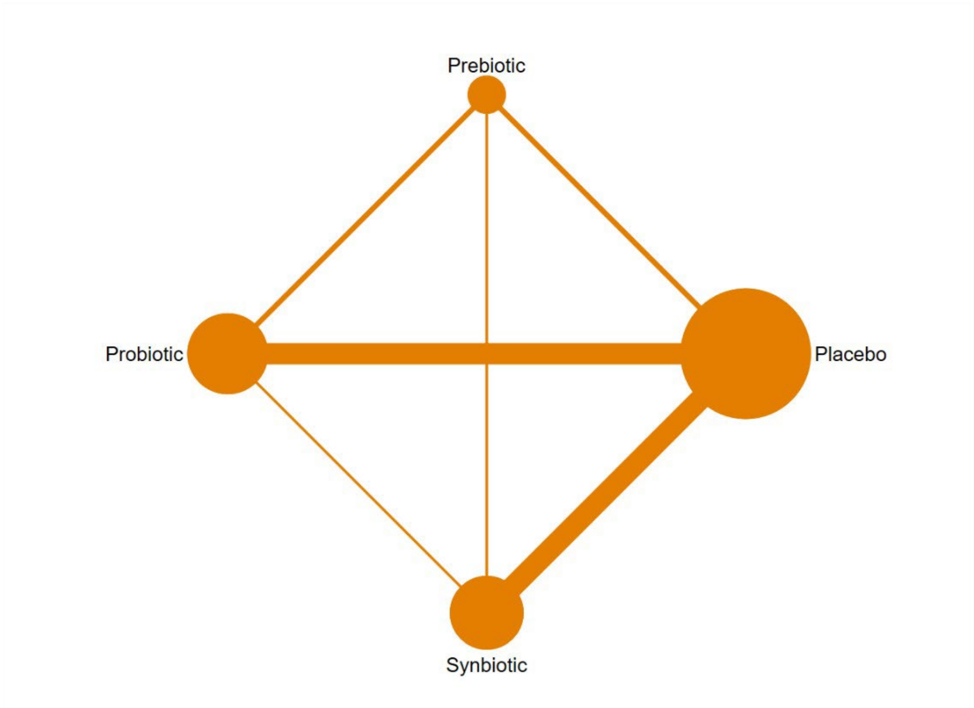

Figure S3 Networks of all option comparisons for reduction in total cholesterol

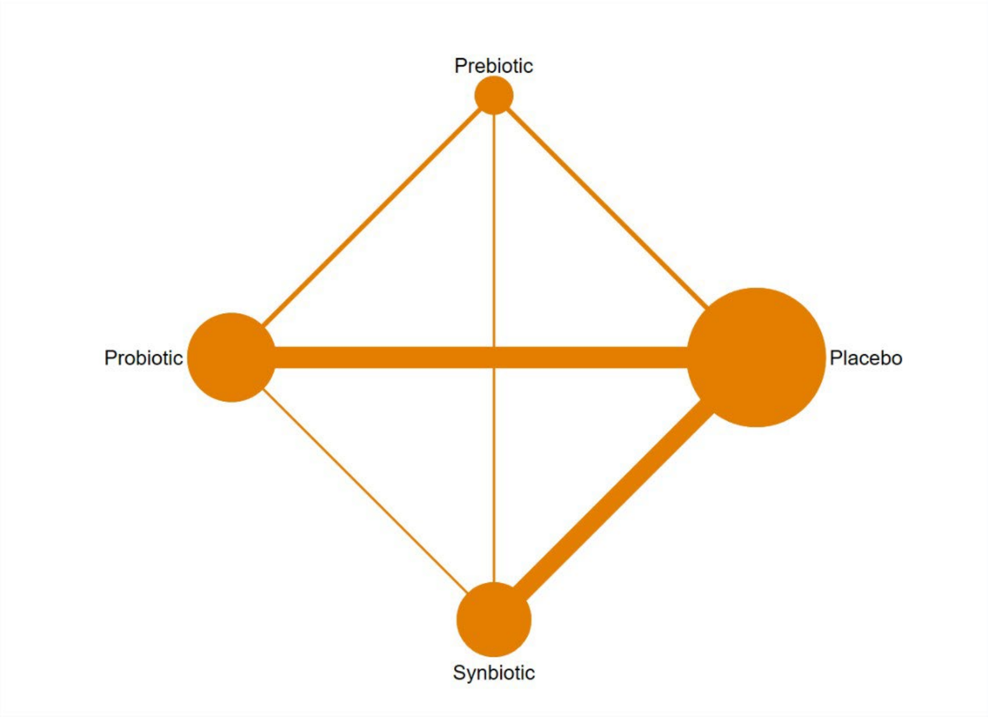

Figure S4 Networks of all option comparisons for reduction in triglycerides

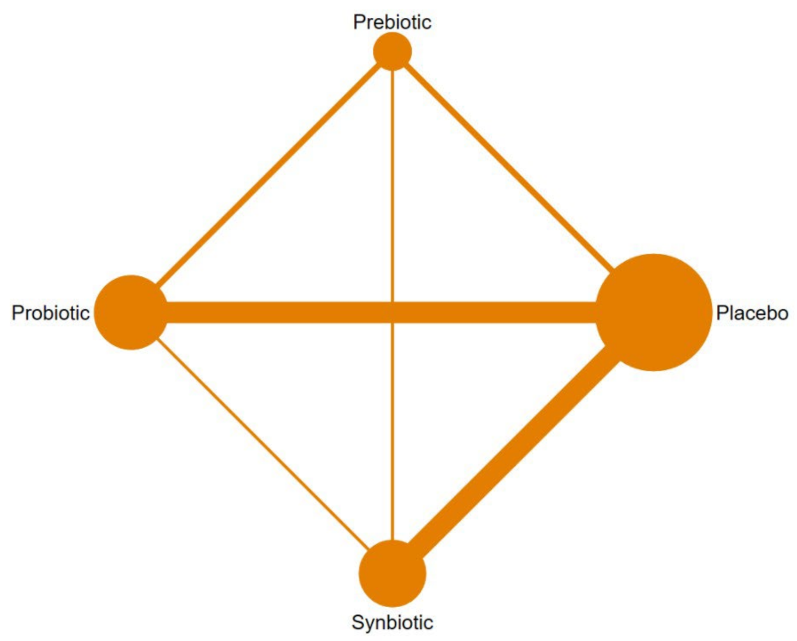

Figure S5 Networks of all option comparisons for reduction in low-density lipoproteins

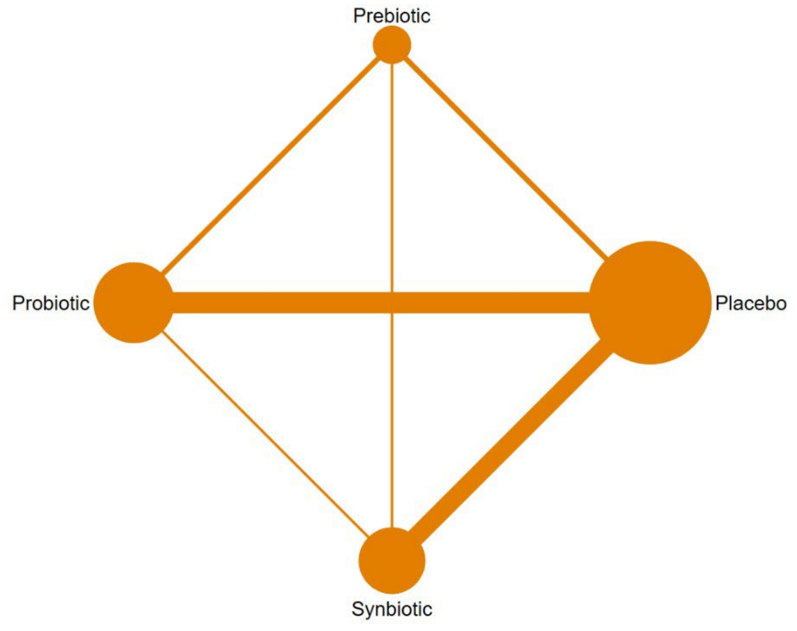

Figure S6 Networks of all option comparisons for increasing in high-density lipoproteins.

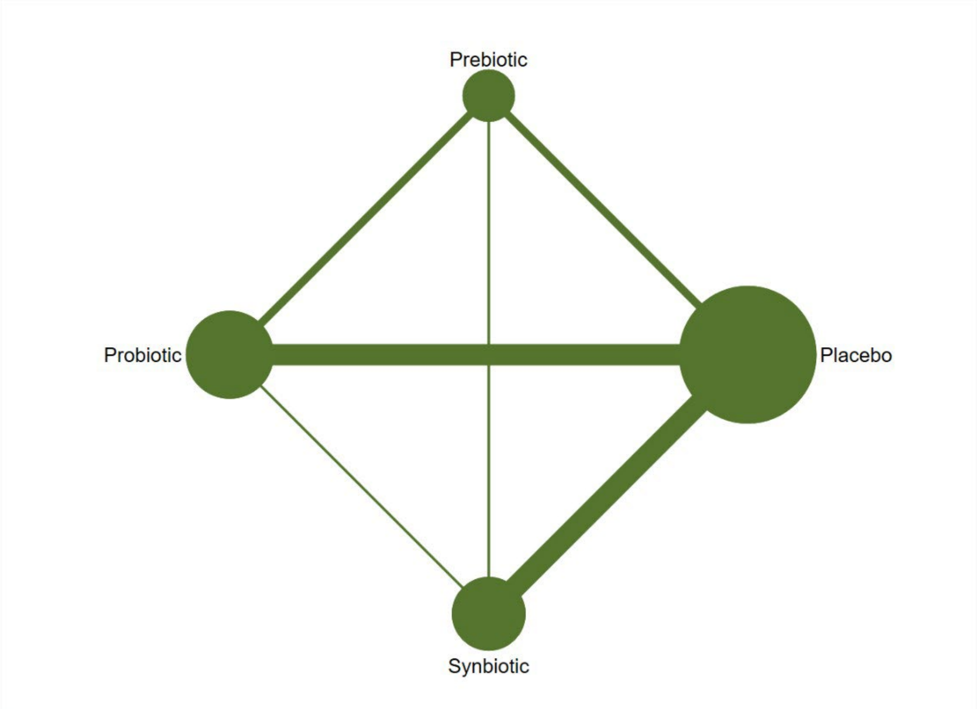

Figure S7 Networks of all option comparisons for reduction in fasting blood sugar

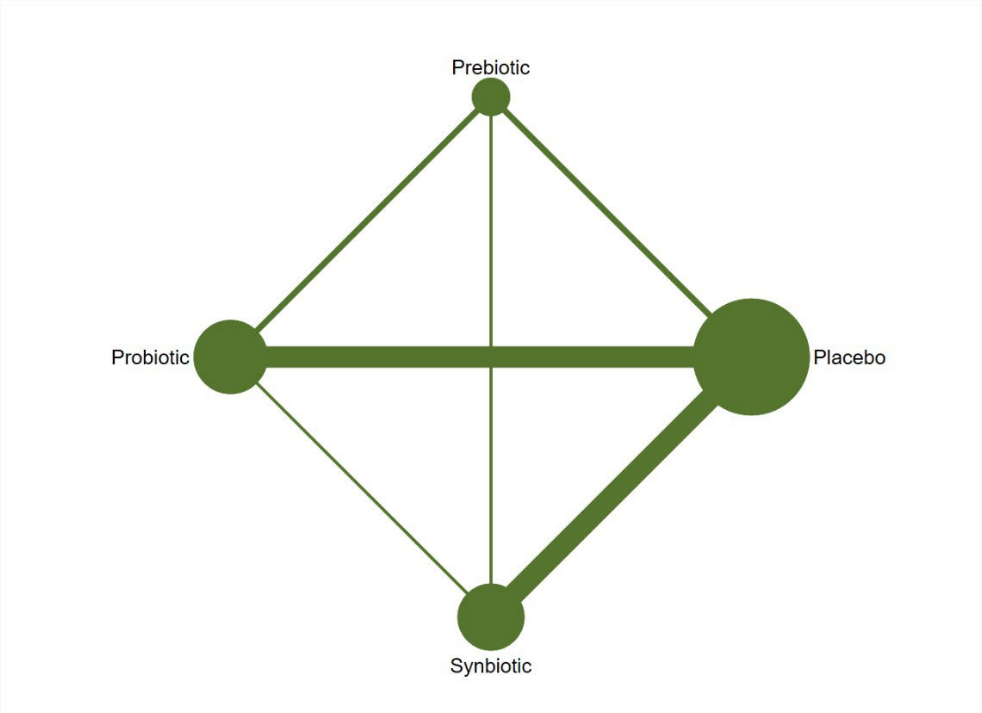

Figure S8 Networks of all option comparisons for reduction in HOMA-IR

Appendix 6: Risk-of-bias assessments

We assess the risk of bias by following the instruction in the Cochrane reviews. This tool addresses specific bias domains, including methods for generating the random sequence; allocation concealment; blinding of participants and investigators; blinding of the outcome assessment; incompleteness of the outcome data; and selective outcome reporting. Each item is adjudicated within each study and the results are represented in a risk-of-bias table. The adjudication of the risk of bias is achieved by answering pre-specified questions about the methods reported by each study in relation to the risk domain, such that the conclusion is either a low risk of bias, an unclear risk of bias, or a high risk of bias.

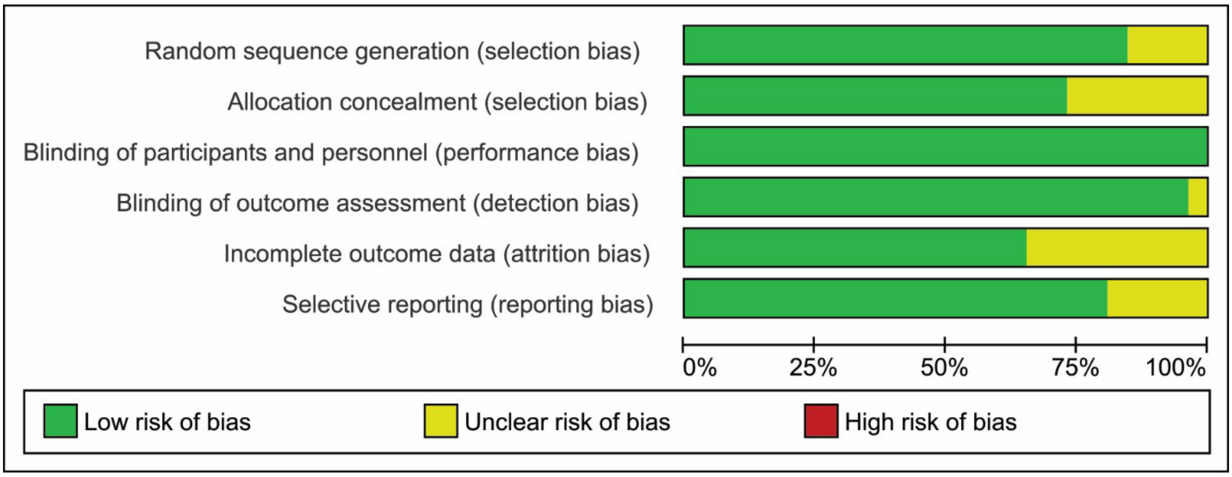

Figure S9 Risk-of-bias summary graph

This reviews the authors' judgments (low, unclear, and high) for each risk-of-bias item shown as percentages across all included studies.

Study	Random sequence generation (selection bias)	Allocation concealment (selection bias)	Blinding of participants and personnel (performance bias)	Blinding of outcome assessment (detection bias)	Incomplete outcome data (attrition bias)	Selective reporting (reporting bias)
Abhari K, 2020	+	+	+	+	?	+
Ahn SB, 2019	+	+	+	+	?	+
Akbarzadeh Z, 2016	?	?	+	+	?	+
Alisi A, 2014	+	+	+	+	+	?
Aller R, 2011	+	+	+	+	+	+
Asgharian A, 2016	+	+	+	+	?	+
Asgharian A, 2017	+	+	+	+	?	+
Bakhshimoghaddam F, 2018	+	?	+	+	?	?
Behrouz V, 2017	+	?	+	+	+	+
Behrouz V, 2020	+	+	+	+	+	?
Chong PL, 2021	+	+	+	?	+	+
Duseja A, 2019	+	+	+	+	+	?
Ekhlasi G, 2016	+	?	+	+	+	+
Eslamparast T, 2014	+	+	+	+	+	?
Famouri F, 2017	+	+	+	+	+	+
Ferolla SM, 2016	+	+	+	+	+	+
Javadi L (1), 2017	+	+	+	+	+	+
Javadi L (2), 2017	+	+	+	+	+	+
Malaguarnera M, 2012	?	+	+	+	+	+
Manzhalii E, 2017	?	?	+	+	?	+
Miccheli A, 2015	+	+	+	+	+	+
Mofidi F, 2017	+	+	+	+	?	+
Monem SMA, 2017	+	?	+	+	+	+
Spideh A, 2015	+	+	+	+	?	+
Vajro P, 2011	?	+	+	+	+	+
Wong VW, 2013	+	?	+	+	+	+

Figure S10 Risk-of-bias summary: judgments about each bias item for each study

Appendix 7: Data extracted for primary outcomes of network meta-analysis

Table S5 AST and ALT changes in patients with non-alcoholic fatty liver disease

ID	Author, year	Population	Intervention	Sample size	AST (IU/L)	ALT (IU/L)
					Mean difference (SD)	Mean difference (SD)
1	Aller R, 2011	Adults, NAFLD	Probiotics	14	-5.70 (13.68)	-7.30 (28.13)
			Placebo	14	4.70 (13.46)	4.10 (33.93)
2	Vajro P, 2011	Children, NAFLD	Probiotics	10	-	-30.20 (30.51)
			Placebo	10	-	-2.00 (27.66)
3	Malaguarnera M, 2012	Adults, NASH	Synbiotics	34	-69.60 (26.06)	-53.90 (22.65)
			Placebo	32	-45.90 (23.66)	-38.00 (25.83)
4	Wong VW, 2013	Adults, NASH	Probiotics	10	-13.00 (31.00)	-26.00 (91.00)
			Placebo	10	23.00 (31.00)	2.00 (41.00)
5	Alisi A, 2014	Children, NAFLD	Probiotics	22	-	-1.00 (4.69)
			Placebo	22	-	8.00 (21.49)
6	Eslamparast T, 2014	Adults, NAFLD	Synbiotics	26	-31.30 (1.25)	-25.10 (1.73)
			Placebo	26	-7.90 (4.94)	-9.50 (1.41)
7	Miccheli A, 2015	Children, NAFLD	Probiotics	15	-28.43 (12.09)	1.87 (10.72)
			Placebo	16	-12.00 (18.62)	7.67 (22.58)
9	Akbarzadeh Z, 2016	Adults, NAFLD	Prebiotics	38	-11.10 (17.29)	-18.60 (25.49)
			Placebo	37	-8.10 (53.82)	-22.80 (66.61)
10	Asghgarian A, 2016	Adults, NAFLD	Synbiotics	38	-1.02 (3.47)	3.13 (3.49)
			Placebo	36	1.78 (1.78)	7.17 (2.36)
11	Ekhlasi G, 2016	Adults, NAFLD	Synbiotics	15	-7.43 (8.58)	-6.54 (7.66)
			Placebo	15	2.50 (5.75)	4.16 (3.43)
12	Ferolla SM, 2016	Adults, NASH	Synbiotics	27	2.49 (90.91)	-14.75 (123.41)
			Placebo	23	5.02 (107.51)	17.30 (114.37)
15	Famouri F, 2017	Children, NAFLD	Probiotics	32	-7.90 (13.60)	-9.70 (16.98)
			Placebo	32	-3.60 (12.39)	-2.70 (13.32)
16	Javadi L, 2017 (1)	Adults, NAFLD	Synbiotics	17	-14.47 (12.50)	-12.29 (12.73)
			Probiotics	20	-14.70 (12.72)	-10.85 (13.04)

ID	Author, year	Population	Intervention	Sample size	AST (IU/L)	ALT (IU/L)
					Mean difference (SD)	Mean difference (SD)
			Prebiotics	19	-9.05 (9.49)	-8.05 (11.08)
			Placebo	19	-1.94 (12.85)	0.21 (12.86)
18	Manzhalii E, 2017	Adults, NASH	Synbiotics	38	-10.40 (2.26)	-17.80 (2.55)
			Placebo	37	5.70 (2.79)	-2.00 (3.00)
19	Mofidi F, 2017	Adults, NAFLD	Synbiotics	21	-10.80 (2.29)	-11.61 (2.89)
			Placebo	21	-1.98 (0.41)	-5.04 (0.60)
20	Monem SMA, 2017	Adults, NASH	Probiotics	15	-5.85 (13.38)	-35.35 (22.15)
			Placebo	15	13.13 (8.62)	-1.00 (11.30)
21	Bakhshimoghaddam F, 2018	Adults, NAFLD	Synbiotics	34	-7.50 (6.10)	-14.50 (15.60)
			Placebo	34	3.10 (5.70)	3.10 (14.40)
22	Ahn SB, 2019	Adults, NAFLD	Probiotics	30	-2.10 (21.62)	-2.30 (25.99)
			Placebo	35	0.50 (17.40)	2.80 (19.99)
23	Duseja A, 2019	Adults, NAFLD	Probiotics	17	-32.00 (27.97)	-56.00 (41.96)
			Placebo	13	-29.30 (26.29)	-37.50 (46.71)
24	Abhari K, 2020	Adults, NAFLD	Synbiotics	22	-9.79 (22.66)	-11.40 (31.55)
			Placebo	24	1.40 (26.24)	3.30 (23.83)
25	Behrouz V, 2020	Adults, NAFLD	Probiotics	30	-41.82 (13.69)	-43.00 (12.53)
			Prebiotics	29	-39.43 (14.49)	-49.33 (19.36)
			Placebo	30	-24.59 (14.20)	-18.59 (27.51)
26	Chong PL, 2021	Adults, NAFLD	Probiotics	19	-2.00 (18.33)	-5.00 (31.51)
			Placebo	16	1.00 (16.09)	-2.00 (23.30)

ALT, alanine aminotransferase; AST, aspartate aminotransferase; IU/L, international units per liter; NAFLD: non-alcoholic fatty liver disease; NASH, non-alcoholic steatohepatitis; SD, standard deviation

Appendix 8: Data extracted for secondary outcomes of network meta-analysis

Table S6 Body mass index and waist circumference changes in patients with non-alcoholic fatty liver disease

ID	Author	Population	Intervention	Sample size	BMI (kg/m^2)	WC (cm)
					Mean difference (SD)	Mean difference (SD)
1	Aller R, 2011	Adults, NAFLD	Probiotics	14	0.90 (4.66)	-
			Placebo	14	0.60 (5.82)	-
2	Vajro P, 2011	Children, NAFLD	Probiotics	10	-0.08 (0.31)	-
			Placebo	10	-0.12 (0.25)	-
3	Malaguarnera M, 2012	Adults, NASH	Synbiotics	34	-0.90 (1.63)	-
			Placebo	32	-1.30 (1.69)	-
4	Wong VW, 2013	Adults, NASH	Probiotics	10	-1.00 (2.30)	-2.00 (6.00)
			Placebo	10	-0.50 (1.10)	1.00 (4.00)
5	Alisi A, 2014	Children, NAFLD	Probiotics	22	-2.20 (0.92)	-
			Placebo	22	0.10 (1.10)	-
7	Miccheli A, 2015	Children, NAFLD	Probiotics	15	-2.70 (2.86)	-
			Placebo	16	1.73 (4.19)	-
9	Akbarzadeh Z, 2016	Adults, NAFLD	Prebiotics	38	-1.30 (3.50)	-2.90 (7.89)
			Placebo	37	-1.00 (3.70)	-0.70 (8.63)
11	Ekhlasi G, 2016	Adults, NAFLD	Synbiotics	15	-0.14 (2.24)	0.26 (6.59)
			Placebo	15	-0.04 (1.97)	0.07 (7.42)
12	Ferolla SM, 2016	Adults, NASH	Synbiotics	27	-0.40 (3.90)	-1.90 (11.01)
			Placebo	23	-0.20 (5.14)	0.90 (13.68)
13	Asgharian A, 2017	Adults, NAFLD	Synbiotics	38	-0.08 (0.62)	-0.82 (1.67)
			Placebo	36	0.51 (2.76)	1.48 (1.72)
14	Behrouz V, 2017	Adults, NAFLD	Probiotics	30	-1.58 (2.45)	-
			Prebiotics	29	-1.50 (4.70)	-
			Placebo	30	-1.24 (4.88)	-
15	Famouri F, 2017	Children, NAFLD	Probiotics	32	-	-1.90 (14.90)
			Placebo	32	-	-1.40 (7.01)
16	Javadi L,2017 (1)		Synbiotics	17	-	-1.00 (9.73)

ID	Author	Population	Intervention	Sample size	BMI (kg/m^2)	WC (cm)
					Mean difference (SD)	Mean difference (SD)
		Adults, NAFLD	Probiotics	20	-	-1.20 (8.63)
			Prebiotics	19	-	-2.00 (7.35)
			Placebo	19	-	1.00 (5.76)
17	Javadi L,2017 (2)	Adults, NAFLD	Synbiotics	17	-0.73 (4.68)	-
			Probiotics	20	0.16 (3.74)	-
			Prebiotics	19	-0.58 (4.51)	-
			Placebo	19	0.18 (2.88)	-
18	Manzhalii E, 2017	Adults, NASH	Synbiotics	38	-5.30 (0.72)	-
			Placebo	37	-2.70 (0.66)	-
22	Ahn SB, 2019	Adults, NAFLD	Probiotics	30	-0.20 (3.60)	-
			Placebo	35	0.30 (22.38)	-
24	Abhari K, 2020	Adults, NAFLD	Synbiotics	22	-0.96 (0.86)	-5.83 (3.78)
			Placebo	24	-0.74 (1.41)	-3.15 (4.59)
25	Behrouz V, 2020	Adults, NAFLD	Probiotics	30	-1.50 (7.78)	-3.10 (5.26)
			Prebiotics	29	-1.40 (3.17)	-3.20 (6.25)
			Placebo	30	-1.20 (7.45)	-3.90 (2.52)

BMI: body mass index; cm: centimeters; kg/m^2: kilogram per square meter; NAFLD: non-alcoholic fatty liver disease; NASH, non-alcoholic steatohepatitis; SD: standard deviation; WC: waist circumference

Table S7 Lipid profile changes in patients with non-alcoholic fatty liver disease

ID	Author	Population	Intervention	Sample size	TC (mg/dL)	TG (mg/dL)	LDL (mg/dL)	HDL (mg/dL)
					Mean difference (SD)	Mean difference (SD)	Mean difference (SD)	Mean difference (SD)
1	Aller R, 2011	Adults, NAFLD	Probiotics	14	6.10 (43.37)	-20.20 (83.70)	11.30 (48.09)	0.00 (11.75)
			Placebo	14	12.00 (48.25)	12.40 (50.28)	11.10 (36.06)	3.20 (8.03)
3	Malaguarnera M, 2012	Adults, NASH	Synbiotics	34	-23.20 (34.42)	-53.20 (69.60)	-32.50 (27.23)	5.03 (28.36)
			Placebo	32	-8.10 (32.20)	-24.20 (74.55)	-6.90 (27.38)	0.78 (28.74)
4	Wong VW, 2013	Adults, NASH	Probiotics	10	0.00 (15.47)	-17.72 (83.79)	3.85 (23.20)	0.00 (3.87)
			Placebo	10	7.73 (19.34)	-26.57 (30.47)	11.60 (19.34)	3.86 (3.87)
5	Alisi A, 2014	Children, NAFLD	Probiotics	22	-	11.00 (36.33)	-	-
			Placebo	22	-	4.00 (41.69)	-	-
7	Miccheli A, 2015	Children, NAFLD	Probiotics	15	-3.50 (31.87)	21.50 (51.10)	16.80 (27.44)	-3.10 (11.49)
			Placebo	16	-2.00 (35.28)	14.67 (47.92)	-3.33 (27.53)	-5.00 (9.58)
11	Ekhlasi G, 2016	Adults, NAFLD	Synbiotics	15	-17.53 (8.91)	-19.03 (20.40)	-15.88 (8.71)	2.20 (7.05)
			Placebo	15	2.83 (4.19)	4.01 (28.71)	1.43 (2.67)	0.30 (7.14)
12	Ferolla SM, 2016	Adults, NASH	Synbiotics	27	3.50 (36.65)	74.00 (333.10)	0.60 (32.41)	-0.60 (9.68)
			Placebo	23	4.60 (37.93)	28.80 (451.06)	-5.10 (29.55)	-0.50 (11.15)
13	Asgharian A, 2017	Adults, NAFLD	Synbiotics	38	-18.05 (8.04)	1.39 (11.86)	-11.55 (6.81)	-7.36 (1.94)
			Placebo	36	12.59 (6.64)	15.29 (20.49)	15.27 (6.12)	0.75 (1.80)
15	Famouri F, 2017	Children, NAFLD	Probiotics	32	-12.25 (53.10)	-11.97 (47.88)	-6.28 (26.51)	0.50 (11.72)
			Placebo	32	-2.57 (23.95)	-4.16 (19.94)	-0.94 (13.60)	2.00 (8.85)
17	Javadi L, 2017 (2)	Adults, NAFLD	Synbiotics	17	-20.94 (37.31)	-17.29 (68.75)	-19.23 (29.80)	6.14 (10.52)
			Probiotics	20	-10.75 (38.18)	-13.80 (57.54)	-19.78 (37.08)	5.78 (8.34)
			Prebiotics	19	-6.31 (33.37)	-8.84 (70.07)	-12.35 (26.49)	2.94 (10.24)
			Placebo	19	-3.47 (21.47)	-1.10 (47.91)	0.58 (21.27)	0.32 (8.27)
18	Manzhalii E, 2017	Adults, NASH	Synbiotics	38	-73.50 (7.73)	-12.40 (18.60)	-	-
			Placebo	37	-42.56 (7.73)	-8.86 (16.40)	-	-
19	Mofidi F, 2017	Adults, NAFLD	Synbiotics	21	-46.09 (55.17)	-32.33 (46.01)	-24.85 (53.66)	3.69 (7.52)
			Placebo	21	-16.00 (21.40)	-9.80 (10.77)	-13.42 (10.95)	0.95 (3.44)
22	Ahn SB, 2017	Adults, NAFLD	Probiotics	30	-21.50 (117.20)	-37.00 (104.10)	-	-3.70 (32.20)
			Placebo	35	-12.40 (38.95)	4.50 (107.83)	-	-2.67 (36.00)

ID	Author	Population	Intervention	Sample size	TC (mg/dL)	TG (mg/dL)	LDL (mg/dL)	HDL (mg/dL)
					Mean difference (SD)	Mean difference (SD)	Mean difference (SD)	Mean difference (SD)
24	Abhari K, 2020	Adults, NAFLD	Synbiotics	22	-10.55 (30.96)	-8.40 (56.83)	-11.45 (42.11)	1.31 (10.34)
			Placebo	24	-5.14 (34.36)	-10.86 (44.38)	-2.73 (33.72)	-0.12 (4.36)
25	Behrouz V, 2020	Adults, NAFLD	Probiotics	30	2.40 (28.65)	-67.90 (51.79)	2.40 (24.22)	-0.33 (8.90)
			Prebiotics	29	-11.2 (38.76)	-68.47 (70.73)	-8.80 (35.60)	-1.83 (7.22)
			Placebo	30	20.40 (29.88)	-17.86 (140.81)	14.64 (25.86)	0.17 (9.94)
26	Chong PL, 2021	Adults, NAFLD	Probiotics	19	-3.48 (51.37)	1.78 (76.95)	-0.77 (42.87)	0.77 (9.69)
			Placebo	16	7.33 (37.59)	24.80 (115.69)	3.09 (33.25)	0.39 (13.35)

HDL: high-density lipoproteins; LDL: low-density lipoproteins; mg/dL, milligram per deciliter; NAFLD: non-alcoholic fatty liver disease; NASH: non-alcoholic steatohepatitis; SD: standard deviation; TC: total cholesterol; TG: triglycerides

Table S8 FBS and HOMA-IR changes in patients with non-alcoholic fatty liver disease

ID	Author	Year	Intervention	Sample size	FBS (mg/dL)	HOMA-IR
					**Mean difference (SD)**	**Mean difference (SD)**
1	Aller R, 2011	Adults, NAFLD	Probiotics	14	-1.40 (26.85)	-0.30 (2.51)
			Placebo	14	-2.30 (29.07)	0.10 (3.30)
3	Malaguarnera M, 2012	Adults, NASH	Synbiotics	34	-11.71 (12.02)	-1.10 (0.52)
			Placebo	32	-12.20 (11.81)	0.60 (0.59)
4	Wong VW, 2013	Adults, NASH	Probiotics	10	14.40 (52.20)	-
			Placebo	10	3.60 (12.60)	-
5	Alisi A, 2014	Children, NAFLD	Probiotics	22	-	-1.00 (1.41)
			Placebo	22	-	-1.20 (2.48)
6	Eslamparast T, 2014	Adults, NAFLD	Synbiotics	26	-	-0.68 (0.24)
			Placebo	26	-	-0.39 (0.16)
7	Miccheli A, 2015	Children, NAFLD	Probiotics	15	3.73 (7.65)	-0.73 (1.59)
			Placebo	16	2.33 (9.05)	-0.83 (2.60)
8	Spideh A, 2015	Adults, NAFLD	Probiotics	21	-4.53 (3.23)	-0.51 (0.36)
			Placebo	21	2.62 (3.44)	0.47 (0.37)
11	Ekhlasi G, 2016	Adults, NAFLD	Synbiotics	15	-10.97 (6.54)	0.12 (0.37)
			Placebo	15	6.90 (7.92)	0.10 (0.21)
12	Ferolla SM, 2016	Adults, NASH	Synbiotics	27	12.01 (177.76)	-
			Placebo	23	21.22 (175.62)	-
13	Asgharian A, 2017	Adults, NAFLD	Synbiotics	38	-4.48 (1.49)	-
			Placebo	36	3.54 (2.40)	-
14	Behrouz V, 2017	Adults, NAFLD	Probiotics	30	-2.64 (10.00)	-0.85 (2.47)
			Prebiotics	29	-5.90 (17.23)	-1.04 (1.69)
			Placebo	20	-2.07 (22.60)	0.10 (5.67)
17	Javadi L, 2017 (2)	Adults, NAFLD	Synbiotics	17	-4.21 (5.54)	-0.50 (0.57)
			Probiotics	20	-2.78 (7.90)	-0.25 (0.47)
			Prebiotics	19	-1.64 (9.29)	-0.30 (0.43)
			Placebo	19	0.53 (8.19)	0.00 (0.35)
18	Manzhalii E, 2017		Synbiotics	38	-1.00 (10.80)	-

ID	Author	Year	Intervention	Sample size	FBS (mg/dL)	HOMA-IR
					Mean difference (SD)	Mean difference (SD)
		Adults, NASH	Placebo	37	1.30 (11.10)	-
19	Mofidi F, 2017	Adults, NAFLD	Synbiotics	21	-17.33 (22.82)	-0.10 (1.01)
			Placebo	21	-2.47 (2.38)	-1.32 (0.14)
21	Bakhshimoghaddam F, 2018	Adults, NAFLD	Synbiotics	34	-	-1.30 (1.69)
			Placebo	34	-	0.20 (1.58)
22	Ahn SB, 2019	Adults, NAFLD	Probiotics	30	-13.30 (72.59)	-24.6 (36.3)
			Placebo	35	0.10 (72.28)	3.2 (32.46)
24	Abhari K, 2020	Adults, NAFLD	Synbiotics	22	-4.42 (18.26)	-0.58 (1.68)
			Placebo	24	-3.37 (6.92)	-0.25 (0.38)
25	Behrouz V, 2020	Adults, NAFLD	Probiotics	30	-2.08 (10.02)	-
			Prebiotics	29	-7.83 (13.41)	-
			Placebo	30	-3.17 (13.39)	-
26	Chong PL, 2021	Adults, NAFLD	Probiotics	19	-	0.00 (1.73)
			Placebo	16	-	-0.10 (1.64)

FBS: fasting blood sugar; HOMA-IR: homeostatic model assessment-insulin resistance; mg/dL, milligram per deciliter; NAFLD: non-alcoholic fatty liver disease; NASH: non-alcoholic steatohepatitis; SD: standard deviation

Appendix 9: Result of network meta-analysis

Table S9 Network-estimated, weighted mean difference of options for reduction in AST (IU/L)

Synbiotics			
-1.09 (-7.80, 5.61)	Probiotics		
-4.29 (-12.83, 4.24)	-3.20 (-11.53, 5.13)	Prebiotics	
**-12.71 (-16.95, -8.47)**	**-11.62 (-17.15, -6.09)**	**-8.42 (-16.27, -0.56)**	Placebo

Table S10 Network-estimated, weighted mean difference of options for reduction in ALT (IU/L)

Probiotics			
-0.80 (-10.22, 8.61)	Prebiotics		
-0.80 (-10.22, 8.61)	-1.05 (-10.72, 8.62)	Synbiotics	
**-14.46 (-21.33, -7.59)**	**-13.65 (-22.69, -4.61)**	**-12.60 (-17.22, -7.98)**	Placebo

Table S11 Network-estimated, weighted mean difference of options for reduction in BMI (kg/m^2)

Synbiotics			
-0.35 (-1.86, 1.16)	Prebiotics		
-0.46 (-1.88, 0.96)	-0.11 (-1.58, 1.36)	Probiotics	
-0.71 (-1.49, 0.08)	-0.36 (-1.69, 0.97)	-0.25 (-1.47, 0.97)	Placebo

Table S12 Network-estimated, weighted mean difference of options for reduction in WC (cm)

Synbiotics			
-1.47 (-3.41, 0.47)	Prebiotics		
**-1.98 (-3.84, -0.11)**	-0.51 (-2.82, 1.80)	Probiotics	
**-2.26 (-2.98, -1.54)**	-0.79 (-2.60, 1.03)	-0.28 (-2.01, 1.45)	Placebo

Table S13 Network-estimated, weighted mean difference of options for reduction in TC (mg/dL)

Synbiotics			
-5.80 (-22.12, 10.51)	Prebiotics		
-12.03 (-24.68, 0.62)	-6.22 (-22.17, 9.72)	Probiotics	
**-22.23 (-29.55, -14.90)**	**-16.42 (-31.57, -1.27)**	-10.20 (-20.96, 0.56)	Placebo

Table S14 Network-estimated, weighted mean difference of options for reduction in TG (mg/dL)

Probiotics			
-2.61 (-27.88, 22.65)	Prebiotics		
-8.21 (-28.78, 12.37)	-5.59 (-33.44, 22.25)	Synbiotics	
**-20.97 (-40.42, -1.53)**	-18.36 (-45.50, 8.78)	**-12.77 (-20.88, -4.66)**	Placebo

Table S15 Network-estimated, weighted mean difference of options for reduction in LDL (mg/dL)

Synbiotics			
-1.85 (-16.45, 12.75)	Prebiotics		
-7.56 (-19.88, 4.75)	-5.72 (-20.09, 8.65)	Probiotics	
**-17.72 (-25.23, -10.22)**	**-15.88 (-29.34, -2.42)**	-10.16 (-20.62, 0.29)	Placebo

Table S16 Network-estimated, weighted mean difference of options for increasing HDL (mg/dL)

Placebo			
0.15 (-3.26, 3.56)	Synbiotics		
0.75 (-3.18, 4.68)	0.60 (-4.34, 5.54)	Probiotics	
1.75 (-3.91, 7.41)	1.60 (-4.68, 7.88)	1.00 (-4.77, 6.77)	Prebiotics

Table S17 Network-estimated, weighted mean difference of options for reduction in FBS (mg/dL)

Synbiotics			
-1.94 (-8.67, 4.79)	Prebiotics		
-3.80 (-9.61, 2.01)	-1.86 (-7.93, 4.21)	Probiotics	
**-6.75 (-10.67, -2.84)**	-4.81 (-10.90, 1.27)	-2.96 (-7.84, 1.92)	Placebo

Table S18 Network-estimated, weighted mean difference of options for reduction in HOMA-IR

Prebiotics			
-0.03 (-1.14, 1.09)	Probiotics		
-0.06 (-1.24, 1.11)	-0.03 (-0.97, 0.91)	Synbiotics	
-0.52 (-1.64, 0.59)	-0.50 (-1.31, 0.32)	-0.46 (-1.06, 0.14)	Placebo

Appendix 10: Surface under the cumulative ranking curves

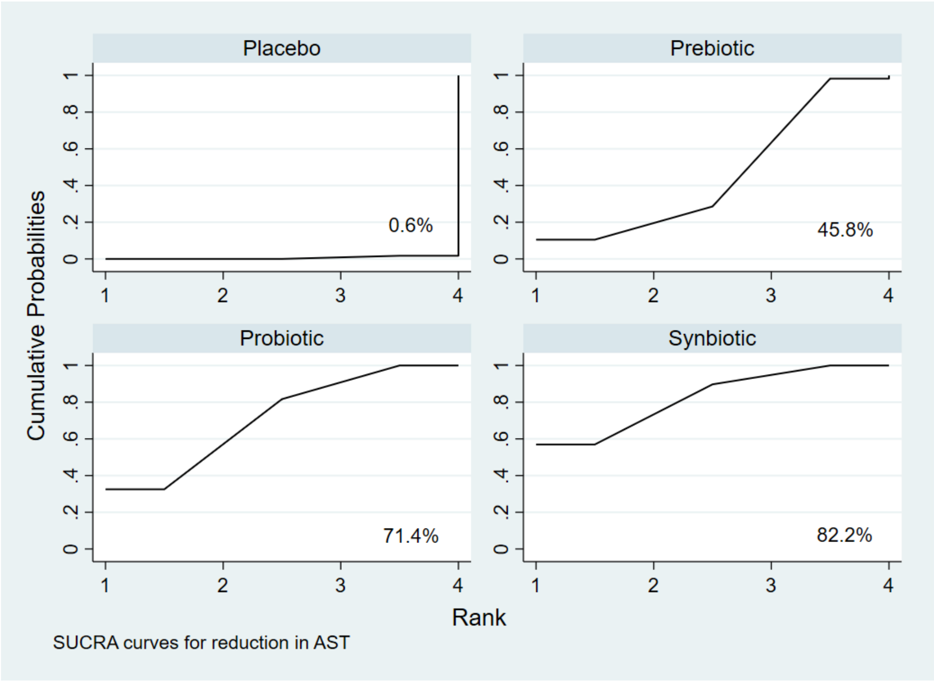

Figure S11 SUCRA curves for reduction in aspartate transaminase

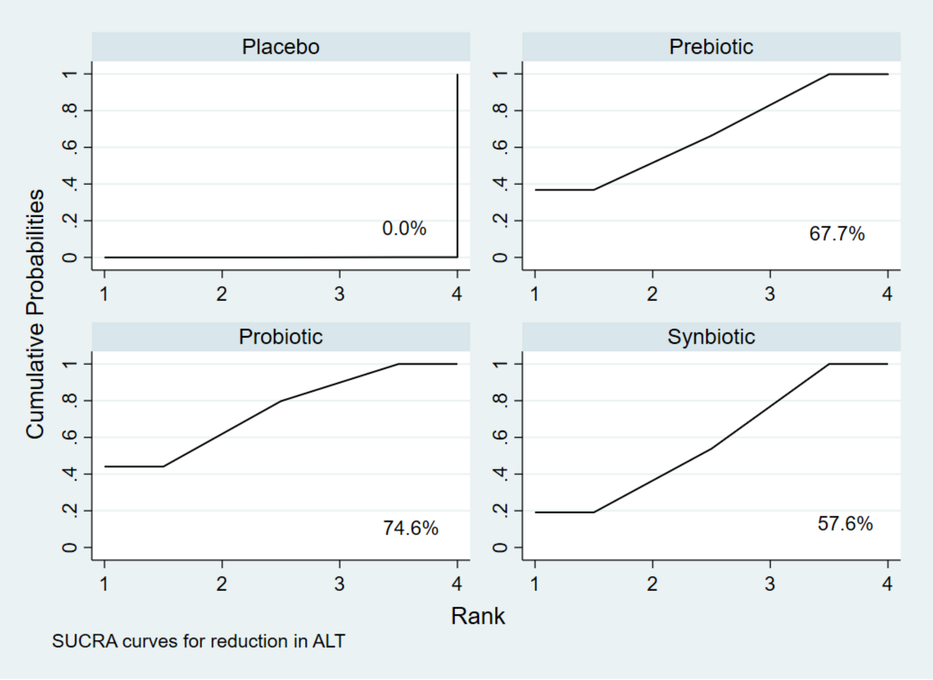

Figure S12 SUCRA curves for reduction in alanine transaminase

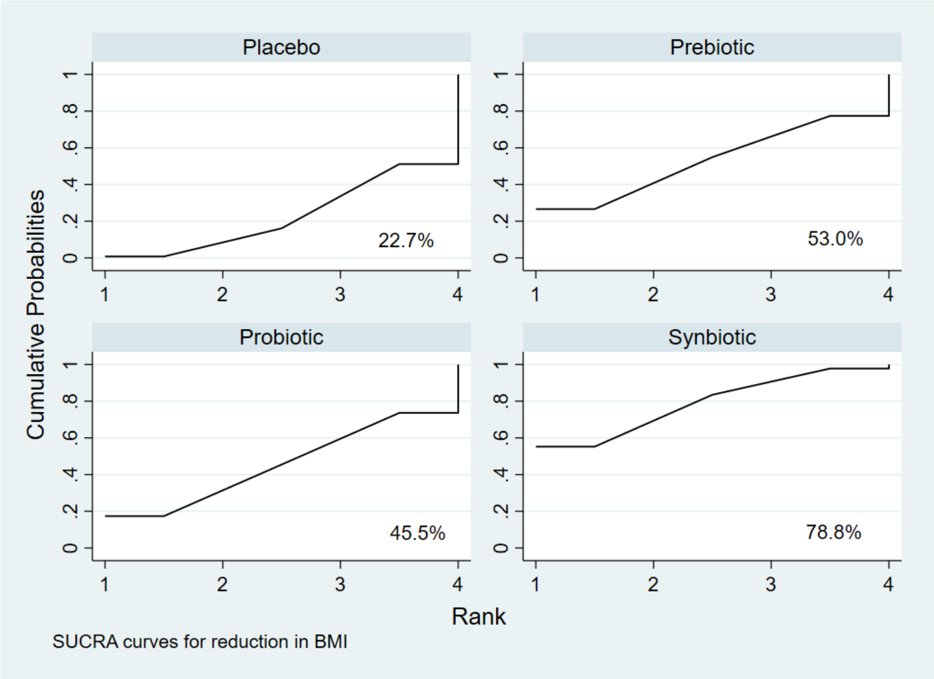

Figure S13 SUCRA curves for reduction in body mass index

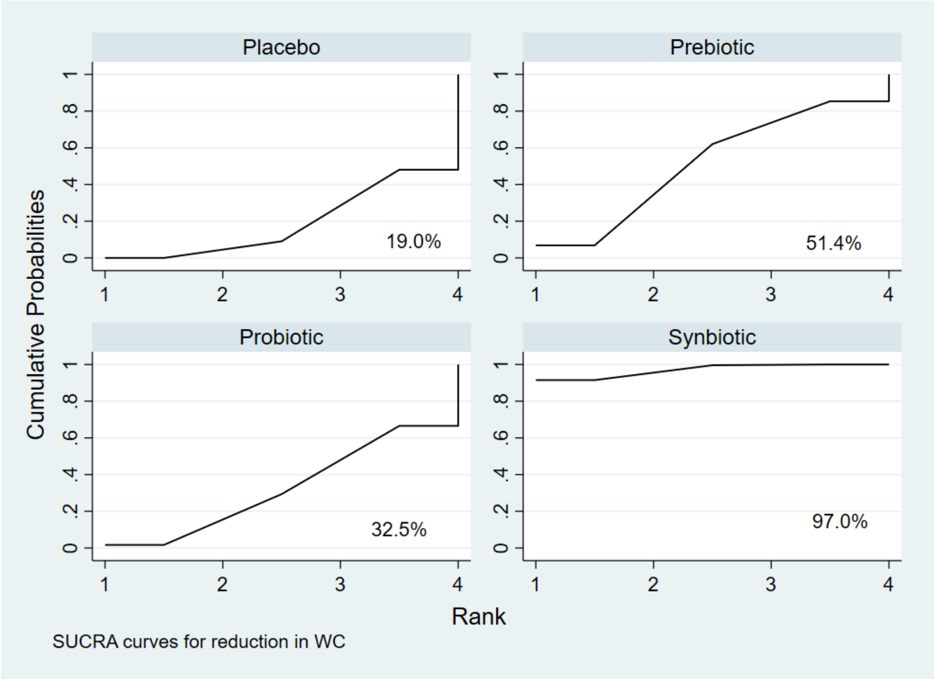

Figure S14 SUCRA curves for reduction in waist circumference

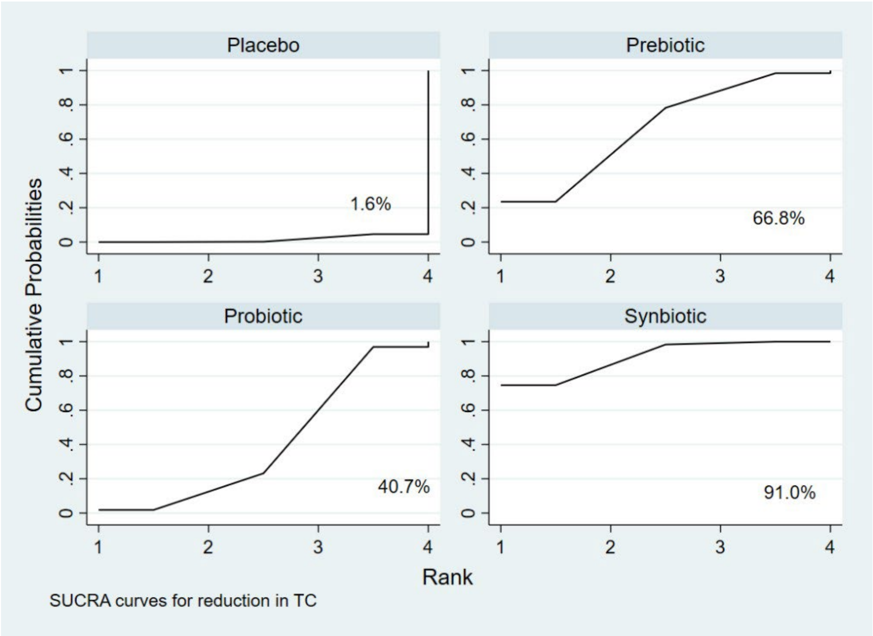

Figure S15 SUCRA curves for reduction in total cholesterol

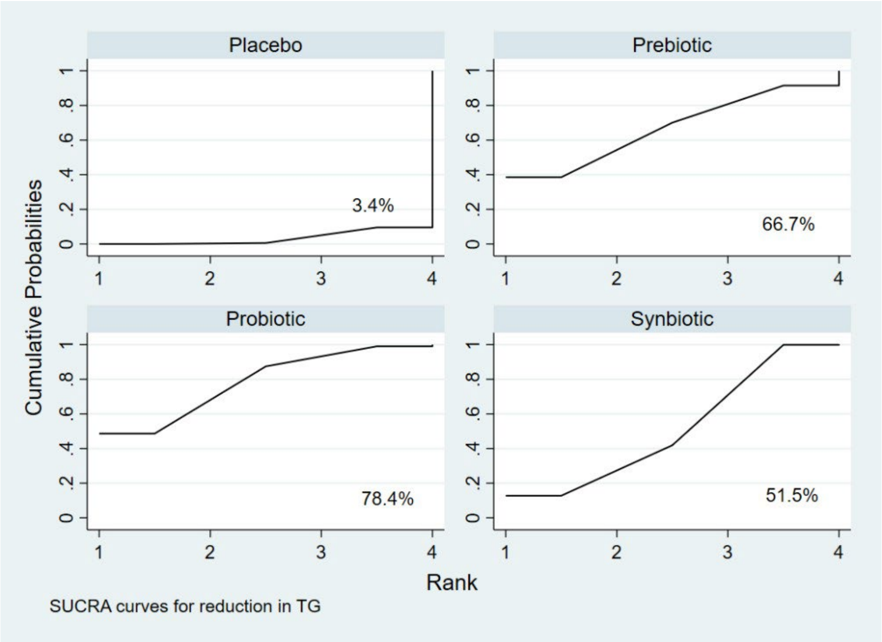

Figure S16 SUCRA curves for reduction in triglycerides

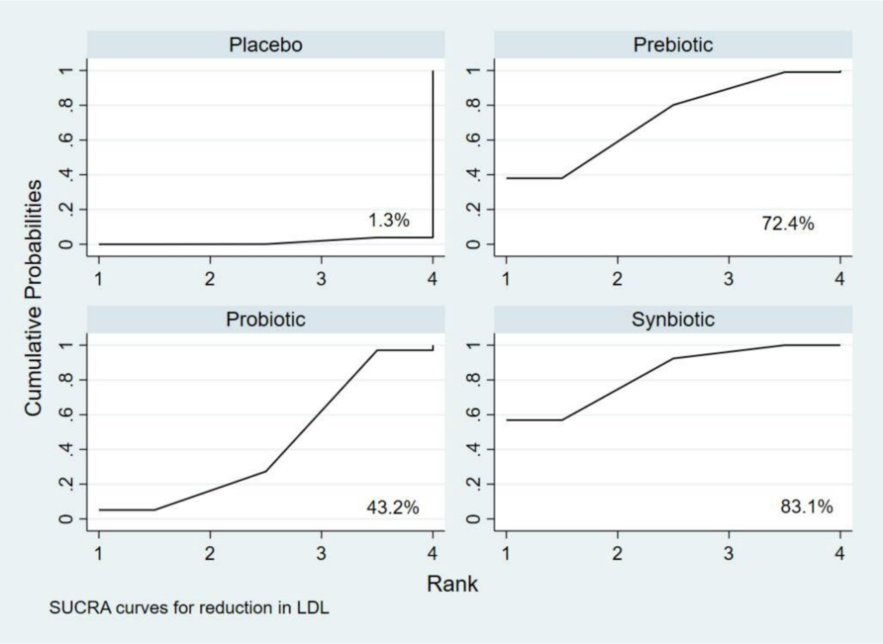

Figure S17 SUCRA curves for reduction in low-density lipoproteins

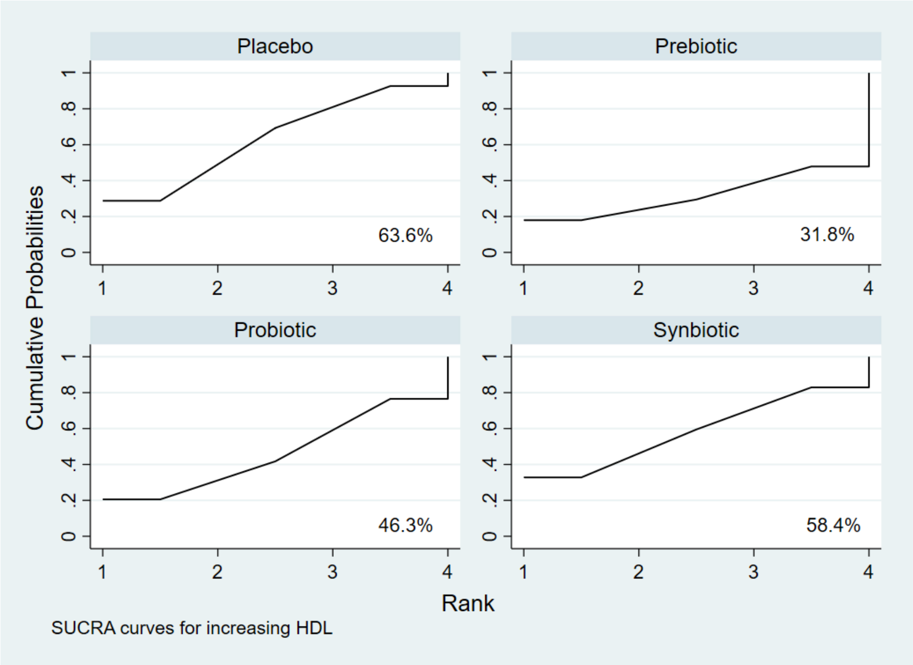

Figure S18 SUCRA curves for increasing high-density lipoproteins

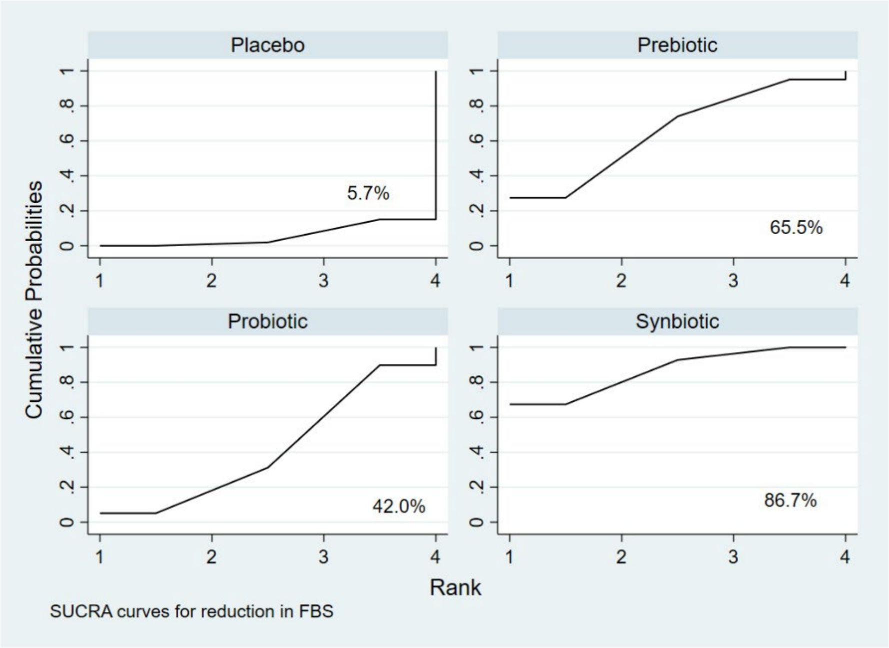

Figure S19 SUCRA curves for reduction in fasting blood sugar

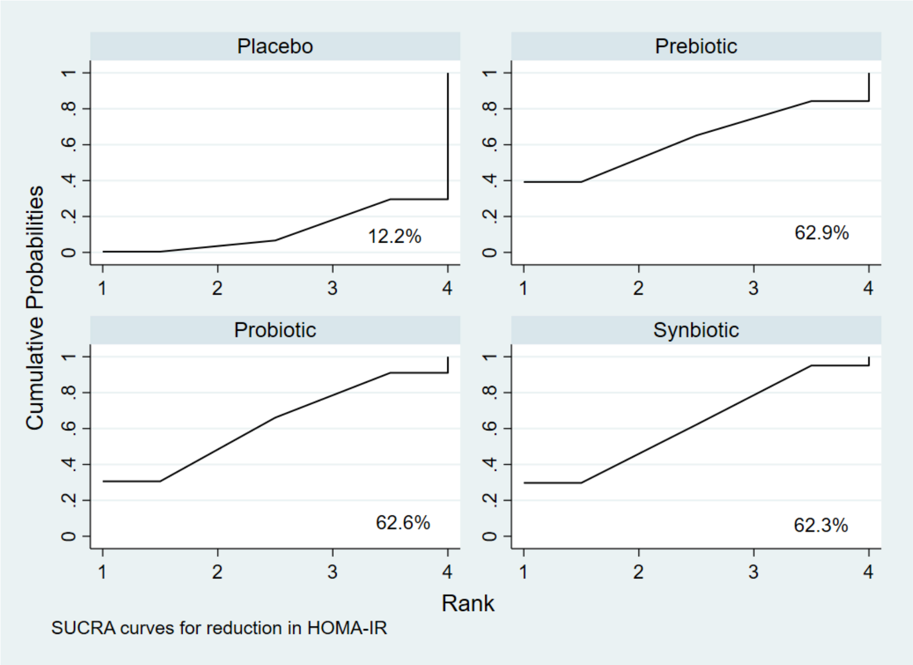

Figure S20 SUCRA curves for reduction in HOMA-IR

Appendix 11: Assessment of inconsistency

Table S19 Assessment of global inconsistency in network meta-analysis

Network outcome	*p*-value for test of global inconsistency
Decreasing AST	0.9369
Decreasing ALT	0.2703
Decreasing BMI	0.9990
Decreasing WC	0.3383
Decreasing TC	0.5528
Decreasing TG	0.7671
Decreasing LDL	0.7586
Increasing HDL	0.6329
Decreasing FBS	0.8826
Decreasing HOMA-IR	0.9837

ALT: alanine aminotransferase; AST: aspartate aminotransferase; BMI: body mass index; FBS: fasting blood sugar; HDL: high-density lipoproteins; HOMA-IR: homeostatic model assessment-insulin resistance; LDL: low-density lipoproteins; TC: total cholesterol; TG: triglycerides; WC: waist circumference

Appendix 12: Comparison-adjusted funnel plots

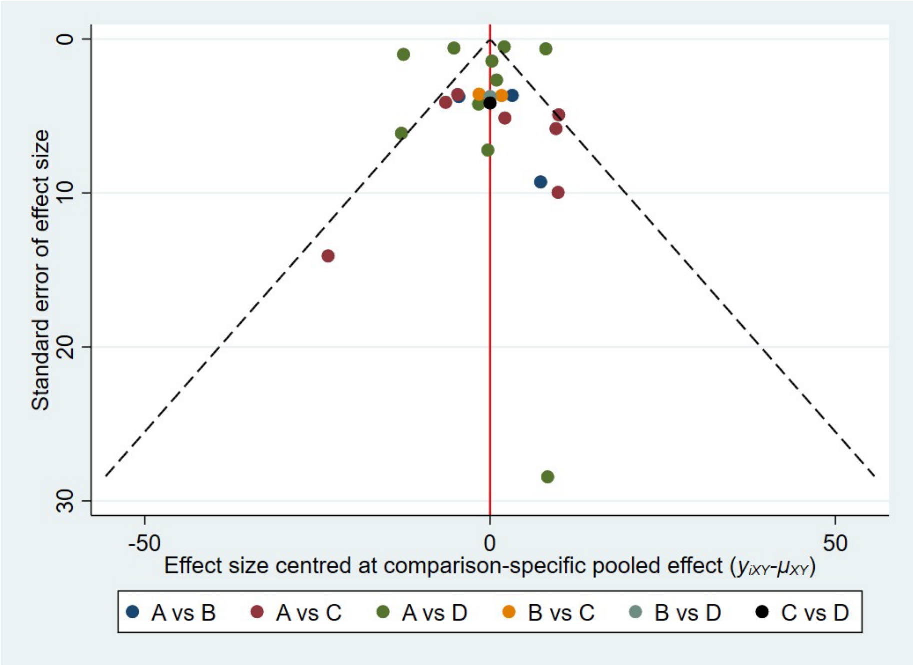

A) Placebo, B) Prebiotics, C) Probiotics, and D) Synbiotics

Figure S21 Comparison-adjusted funnel plot for the network of reduction in AST in all comparisons

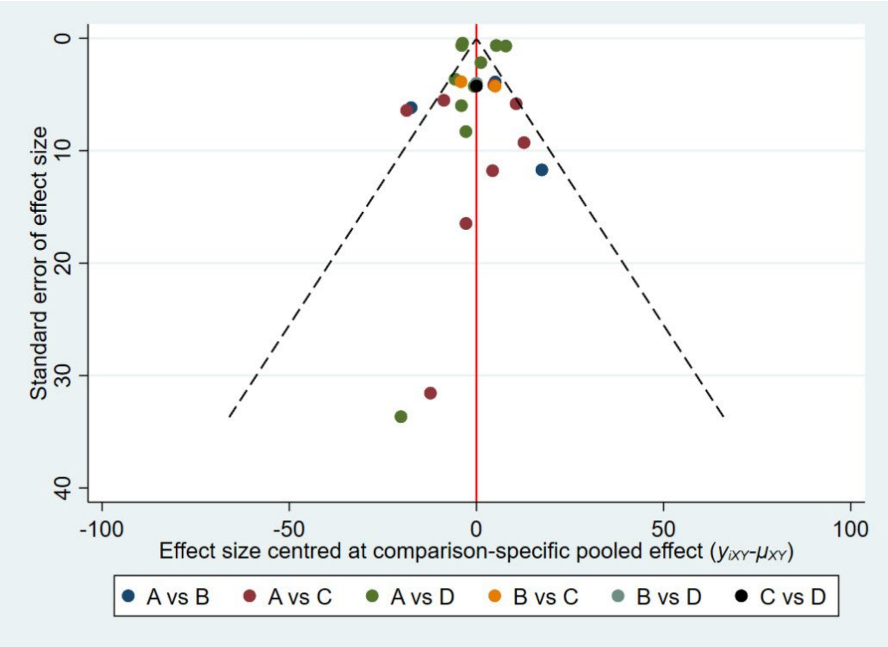

A) Placebo, B) Prebiotics, C) Probiotics, and D) Synbiotics

Figure S22 Comparison-adjusted funnel plot for the network of reduction in ALT in all comparisons

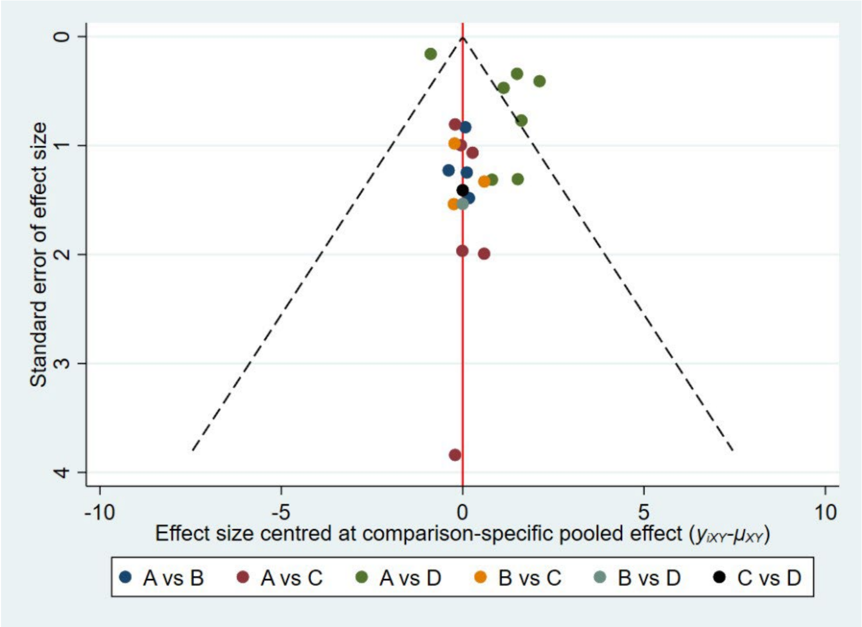

A) Placebo, B) Prebiotics, C) Probiotics, and D) Synbiotics

Figure S23 Comparison-adjusted funnel plot for the network of reduction in BMI in all comparisons

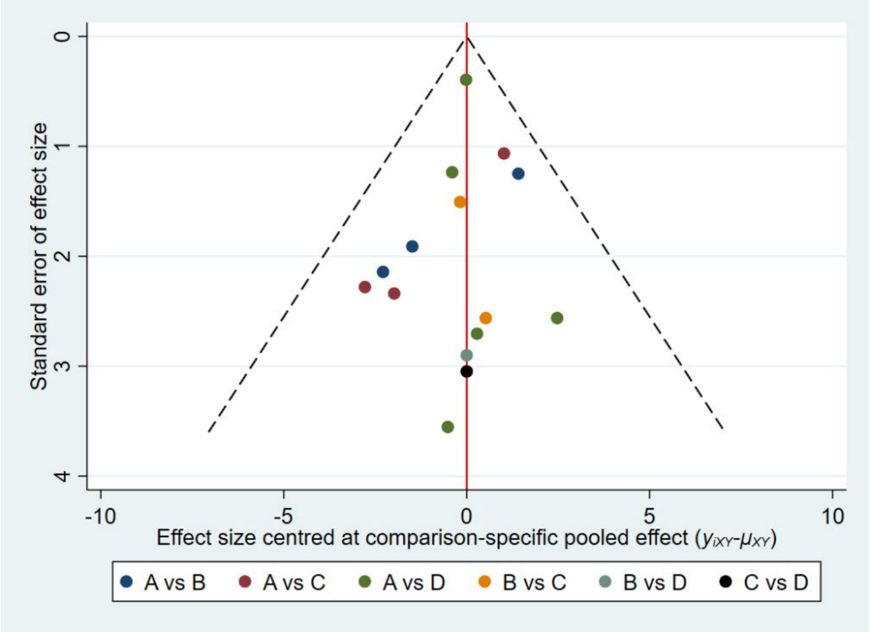

A) Placebo, B) Prebiotics, C) Probiotics, and D) Synbiotics

Figure S24 Comparison-adjusted funnel plot for the network of reduction in WC in all comparisons

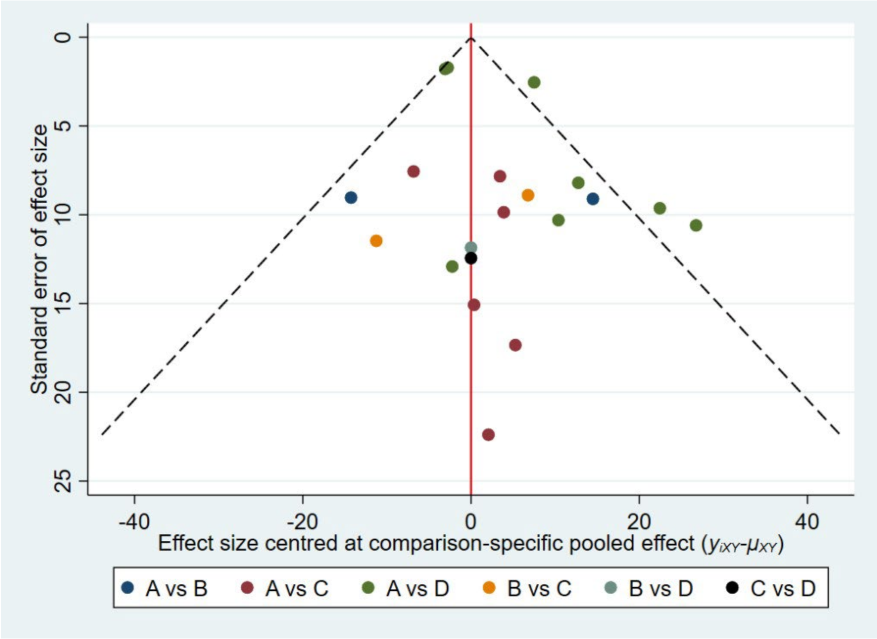

A) Placebo, B) Prebiotics, C) Probiotics, and D) Synbiotics

Figure S25 Comparison-adjusted funnel plot for the network of reduction in TC in all comparisons

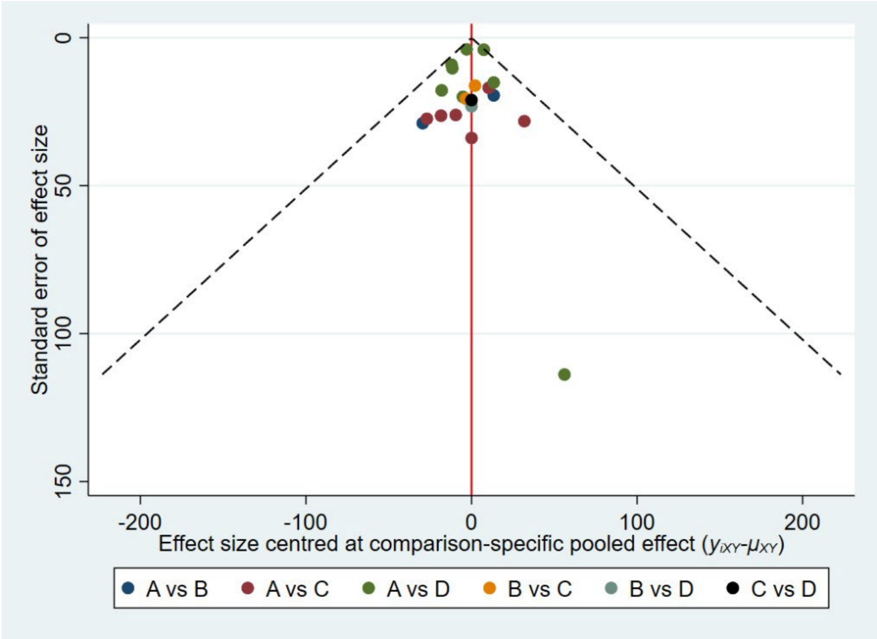

A) Placebo, B) Prebiotics, C) Probiotics, and D) Synbiotics

Figure S26 Comparison-adjusted funnel plot for the network of reduction in TG in all comparisons

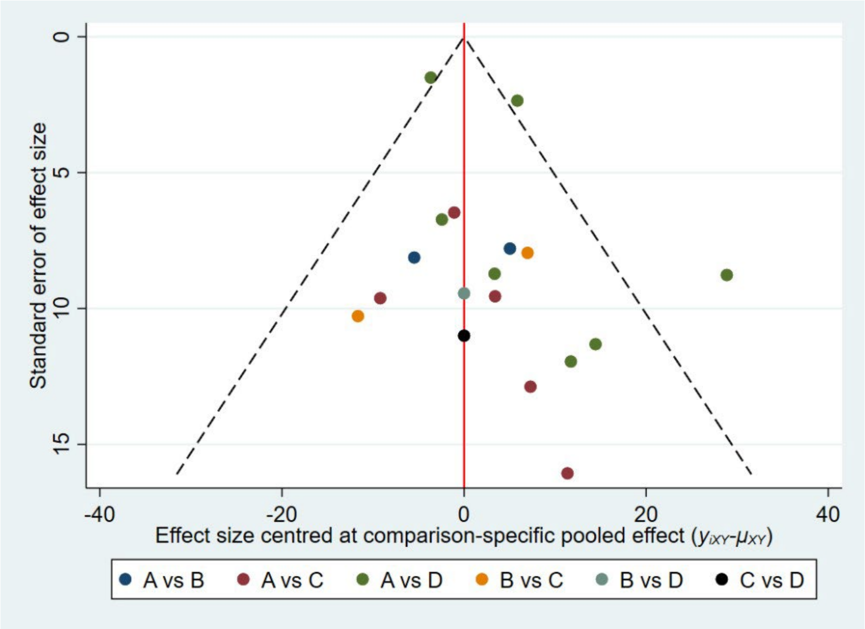

A) Placebo, B) Prebiotics, C) Probiotics, and D) Synbiotics

Figure S27 Comparison-adjusted funnel plot for the network of reduction in LDL in all comparisons

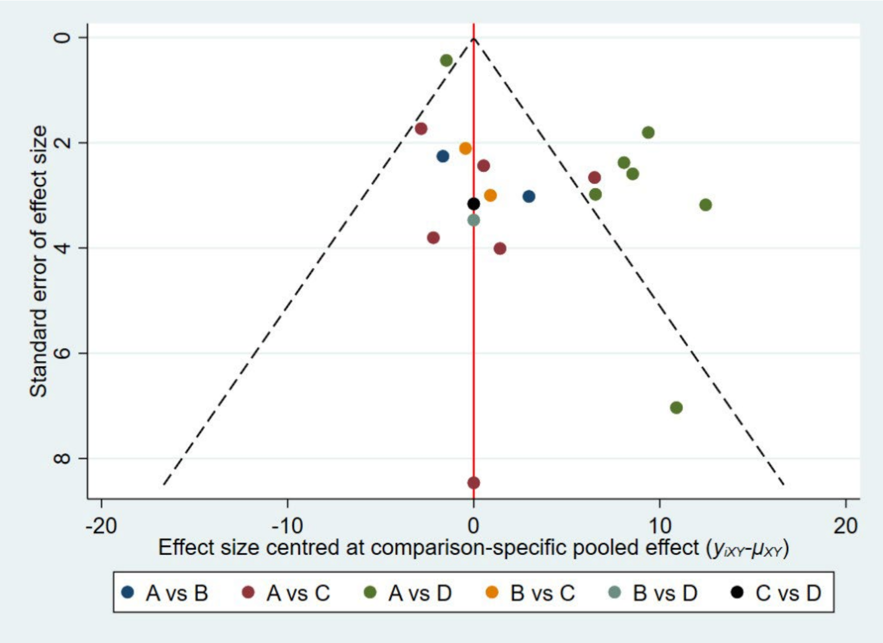

A) Placebo, B) Prebiotics, C) Probiotics, and D) Synbiotics

Figure S28 Comparison-adjusted funnel plot for the network of increasing HDL in all comparisons

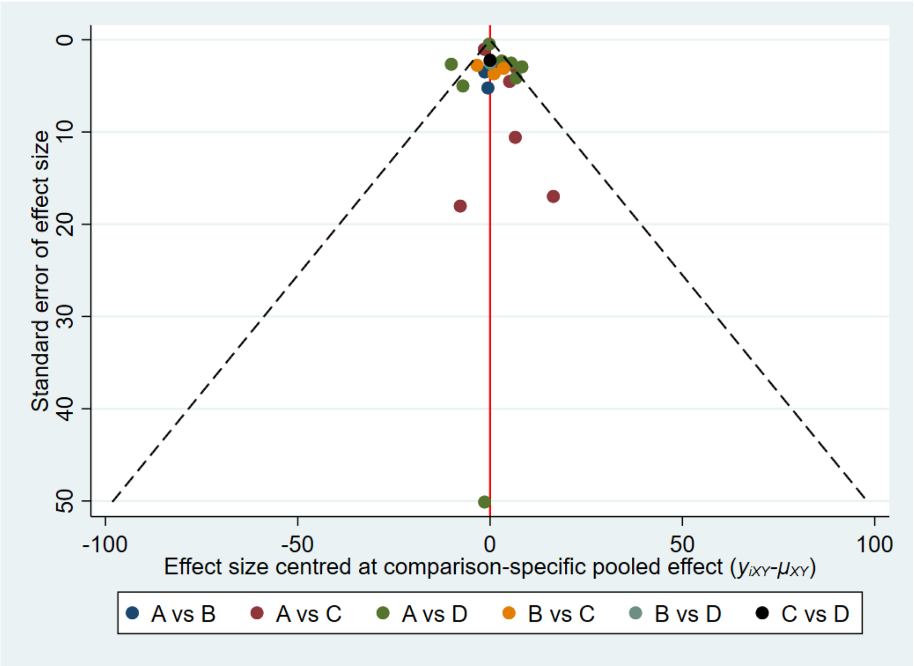

A) Placebo, B) Prebiotics, C) Probiotics, and D) Synbiotics

Figure S29 Comparison-adjusted funnel plot for the network of reduction in FBS in all comparisons

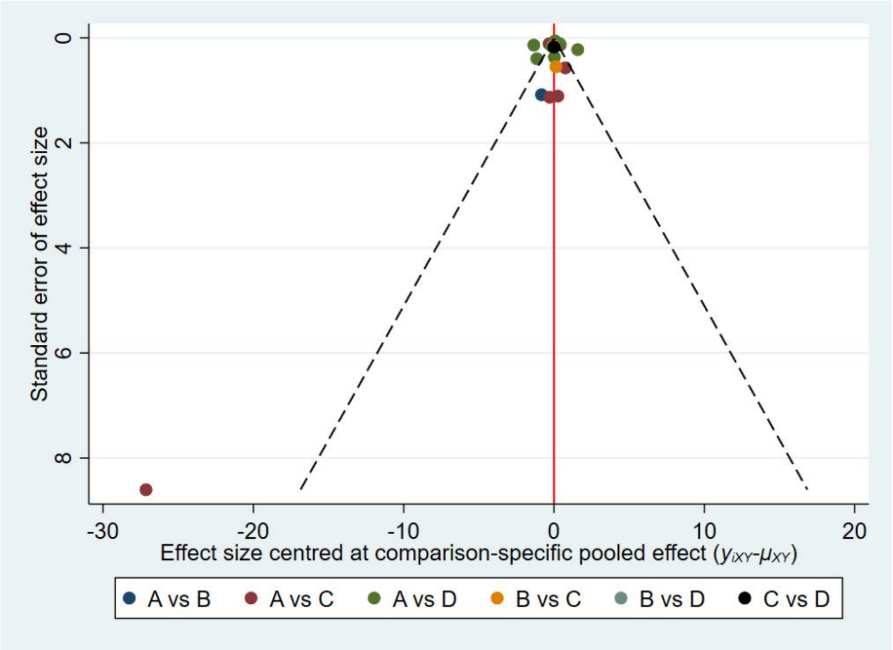

A) Placebo, B) Prebiotics, C) Probiotics, and D) Synbiotics

Figure S30 Comparison-adjusted funnel plot for the network of reduction in HOMA-IR in all comparisons

Appendix 13: Sensitivity analyses of AST outcomes

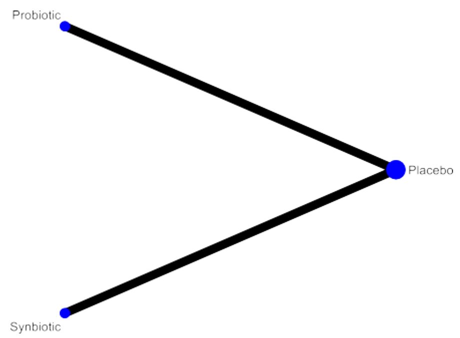

(a)

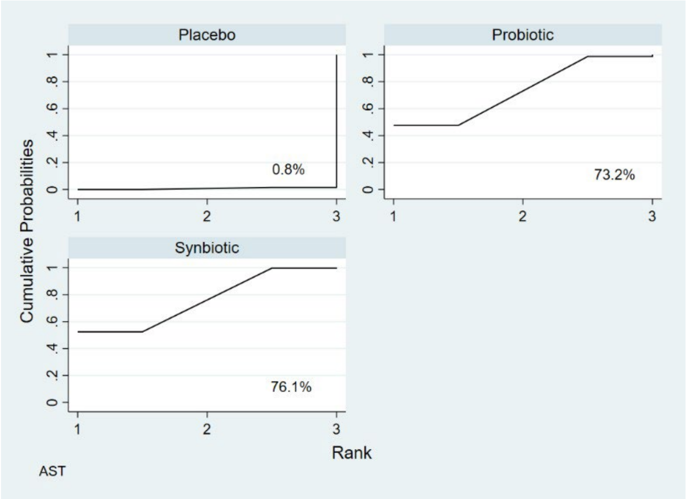

(b)

Figure S31 Sensitivity analysis of the reduction in AST among biopsy-proven NASH patients. (a) network graph and (b) SUCRA curves

Table S20 Network-estimated, weighted mean difference of options for reduction in AST among biopsy-proven NASH patients**.**

Synbiotics		
-0.94 (-28.86,26.97)	Probiotics	
**-22.34 (-38.02, -6.67)**	**-21.40 (-39.86, -2.94)**	Placebo

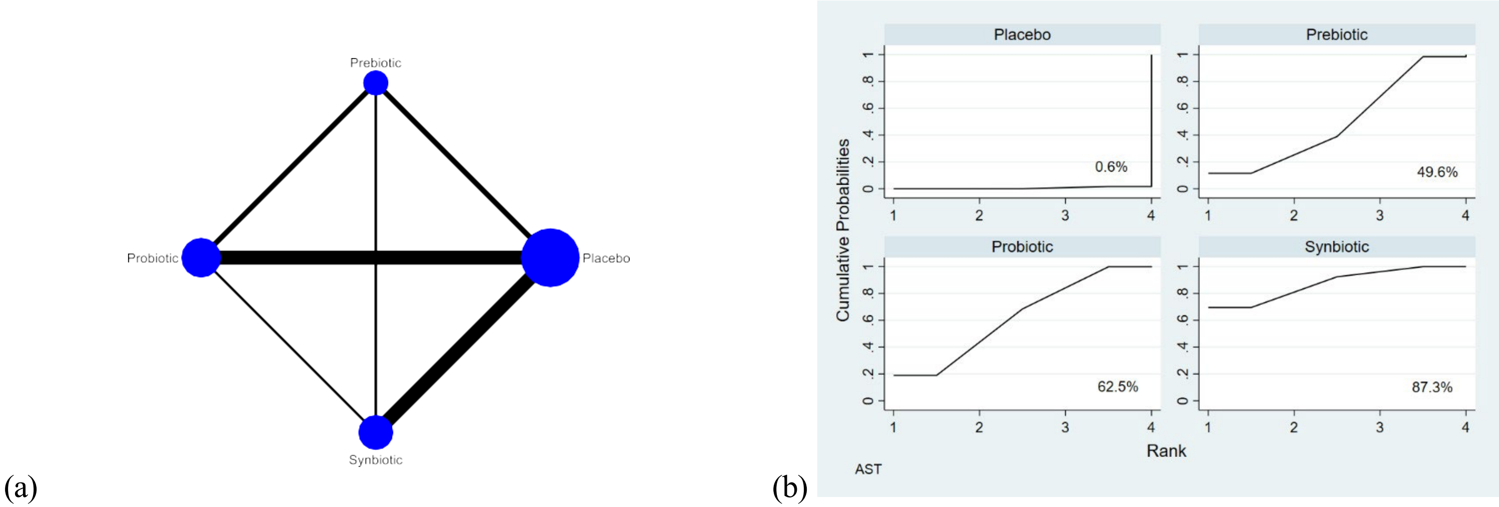

Figure S32 Sensitivity analysis of the reduction in AST duration of treatment not less than 12 weeks among NAFLD patients. (a) network graph and (b) SUCRA curves

Table S21 Network-estimated, weighted mean difference of options for reduction in AST duration of treatment not less than 12 weeks among NAFLD patients**.**

Synbiotics			
-2.95 (-10.75, 4.85)	Probiotics		
-4.69 (-13.74, 4.36)	-1.73 (-10.25, 6.78)	Prebiotics	
**-13.61 (-18.97, -8.24)**	**-10.65 (-17.09, -4.22)**	**-8.92 (-17.11, -0.72)**	Placebo

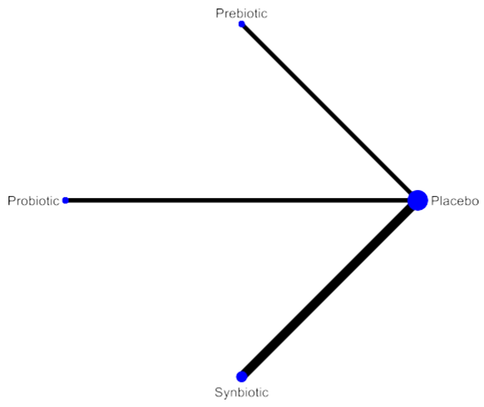

(a)

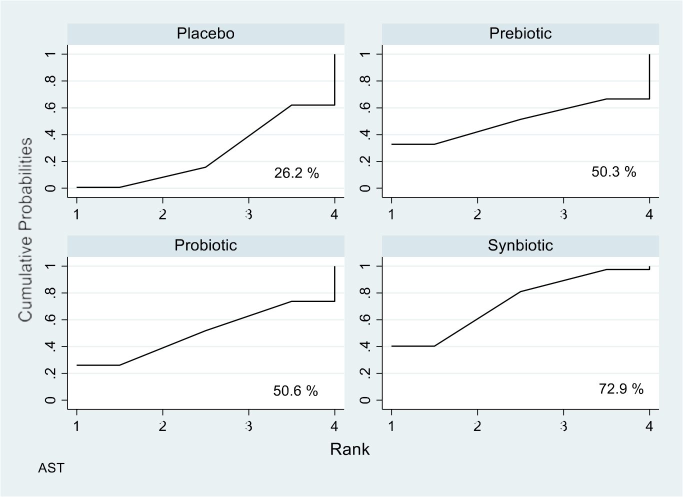

(b)

Figure S33 Sensitivity analysis of the reduction in AST duration of treatment less than 12 weeks among NAFLD patients. (a) network graph and (b) SUCRA curves

Table S22 Network-estimated, weighted mean difference of options for reduction in AST duration of treatment less than 12 weeks among NAFLD patients**.**

Synbiotics			
-2.89 (-19.11, 13.32)	Probiotics		
-2.89 (-24.43, 18.64)	0.00 (-25.05, 25.05)	Prebiotics	
-5.89 (-12.94, 1.15)	-3.00 (-17.60, 11.60)	-3.00 (-23.35, 17.35)	Placebo

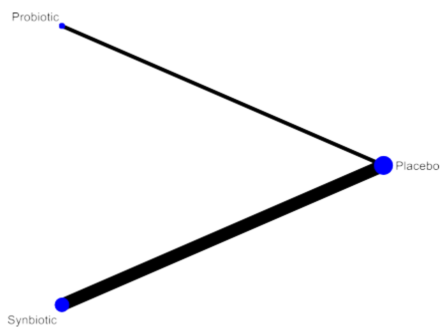

(a)

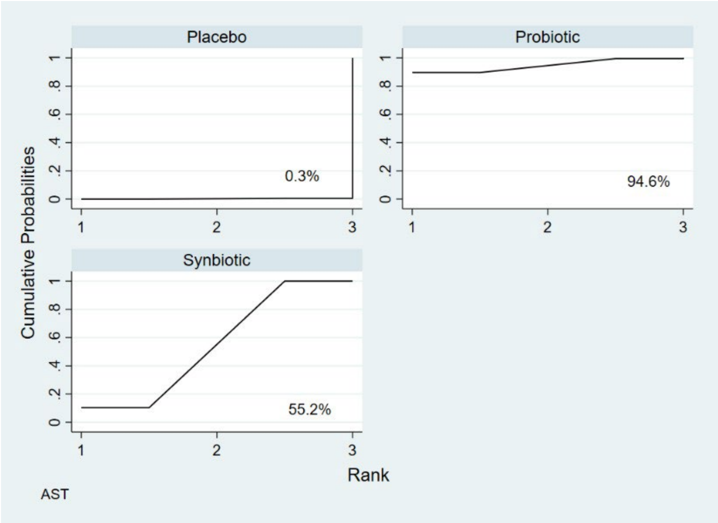

(b)

Figure S34 Sensitivity analysis of the reduction in AST duration of treatment not less than 12 weeks among NASH patients. (a) network graph and (b) SUCRA curves

Table S23 Network-estimated, weighted mean difference of options for reduction in AST duration of treatment not less than 12 weeks among NASH patients**.**

Probiotics		
-18.99 (-48.17, 10.20)	Synbiotics	
**-36.00 (-64.06, -7.94)**	**-17.01 (-25.03, -8.99)**	Placebo

(a)

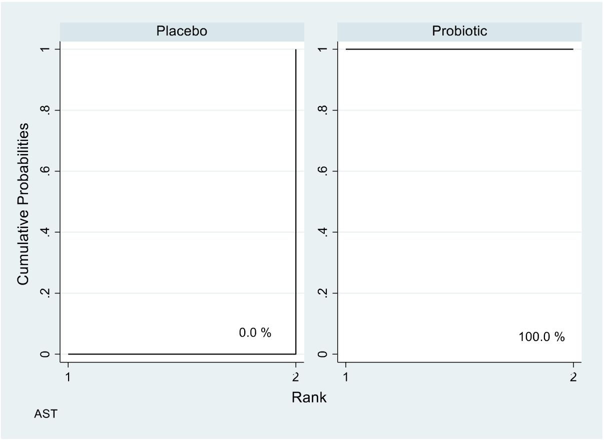

(b)

Figure S35 Sensitivity analysis of the reduction in AST duration of treatment less than 12 weeks among NASH patients. (a) network graph and (b) SUCRA curves

Table S24 Network-estimated, weighted mean difference of options for reduction in AST duration of treatment less than 12 weeks among NASH patients**.**

Probiotics	
**-18.98 (-25.37, -12.59)**	Placebo

Appendix 14: Sensitivity analyses of ALT outcomes

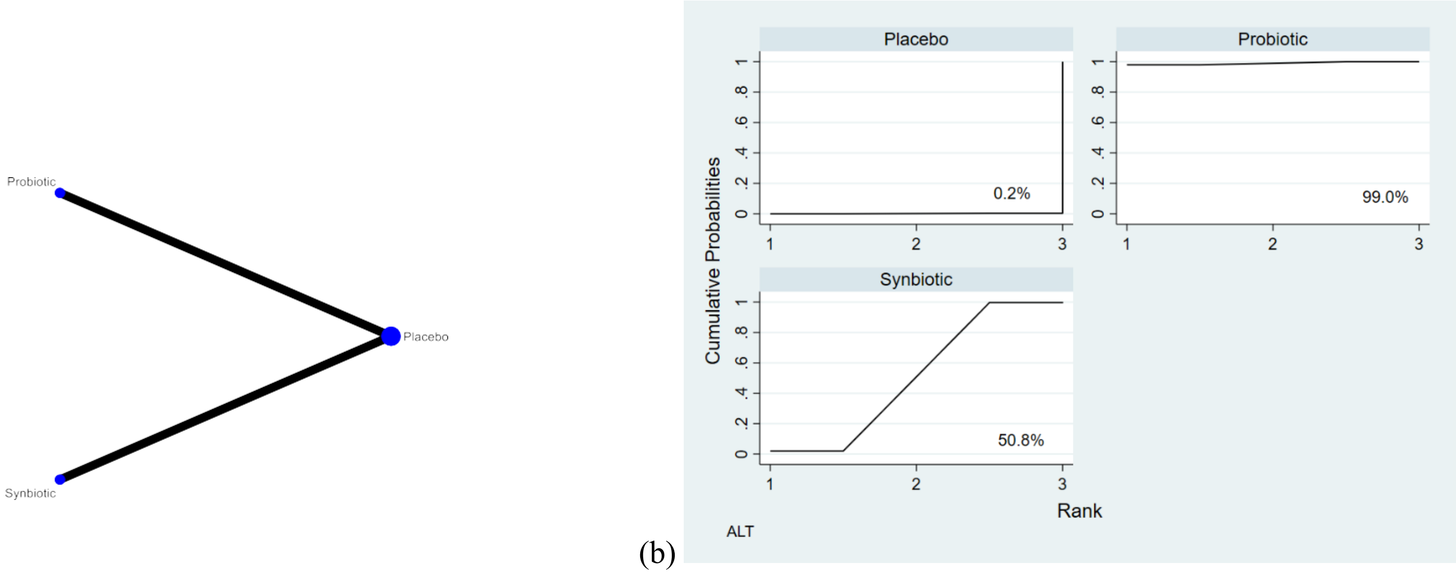

(a) (b)

Figure S36 Sensitivity analysis of the reduction in ALT among biopsy-proven NASH patients. (a) network graph and (b) SUCRA curves

Table S25 Network-estimated, weighted mean difference of options for reduction in ALT among biopsy-proven NASH patients**.**

Probiotics		
**-17.70 (-34.61, -0.79)**	Synbiotics	
**-34.10 (-46.43, -21.77)**	**-16.40 (-27.96, -4.83)**	Placebo

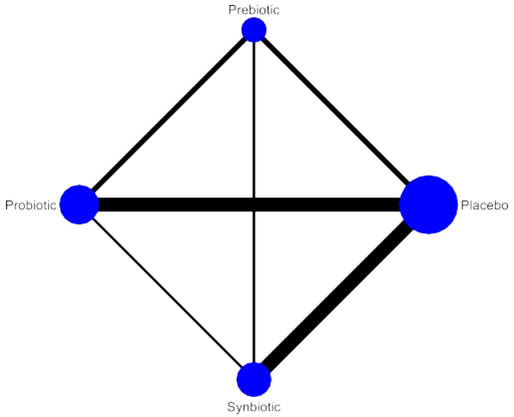

(a)

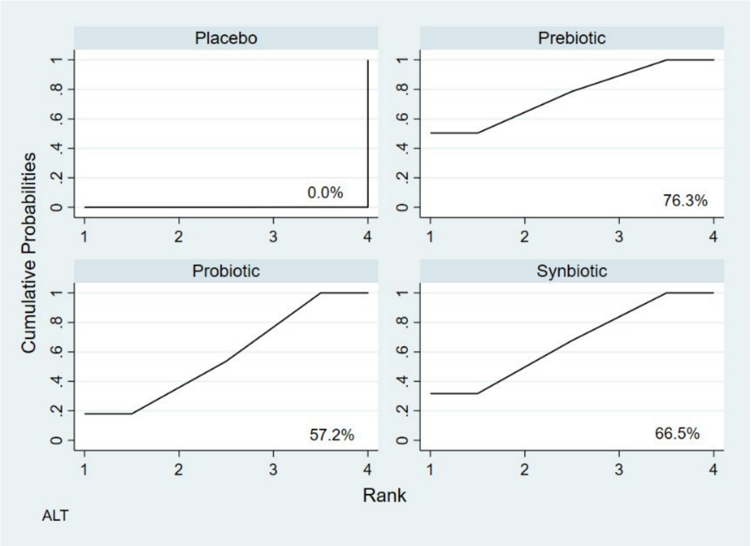

(b)

Figure S37 Sensitivity analysis of the reduction in ALT duration of treatment not less than 12 weeks among NAFLD patients. (a) network graph and (b) SUCRA curves

Table S26 Network-estimated, weighted mean difference of options for reduction in ALT duration of treatment not less than 12 weeks among NAFLD patients**.**

Prebiotics			
-1.30 (-11.09, 8.49)	Synbiotics		
-2.27 (-11.39, 6.86)	-0.97 (-9.57, 7.63)	Probiotics	
**-14.85 (-24.06, -5.64)**	**-13.55 (-19.20, -7.90)**	**-12.58 (-20.11, -5.06)**	Placebo

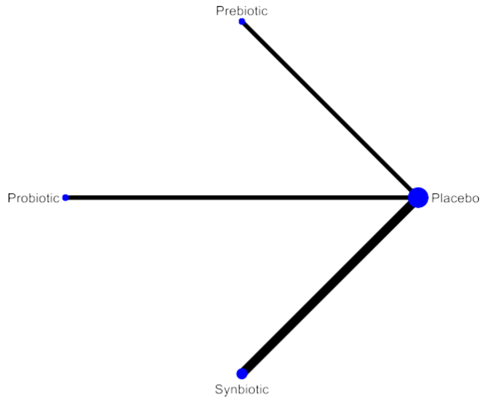

(a)

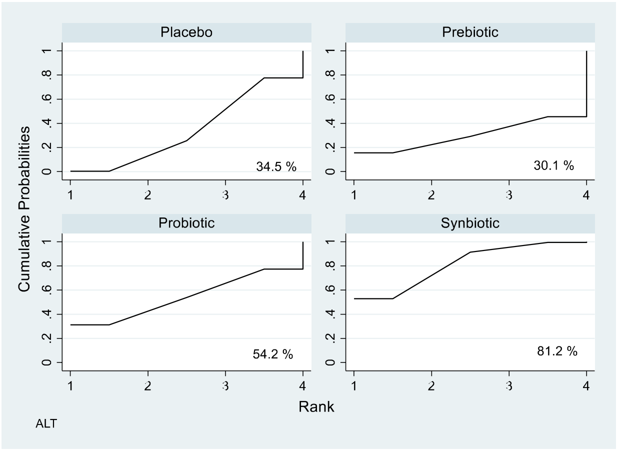

(b)

Figure S38 Sensitivity analysis of the reduction in ALT duration of treatment less than 12 weeks among NAFLD patients. (a) network graph and (b) SUCRA curves

Table S27 Network-estimated, weighted mean difference of options for reduction in ALT duration of treatment less than 12 weeks among NAFLD patients**.**

Synbiotics			
-4.05 (-25.25, 17.14)	Probiotics		
**-7.05 (-13.61, -0.50)**	-3.00 (-23.16, 17.16)	Placebo	
-11.25 (-36.64, 14.14)	-7.20 (-38.95, 24.55)	-4.20 (-28.73, 20.33)	Prebiotics

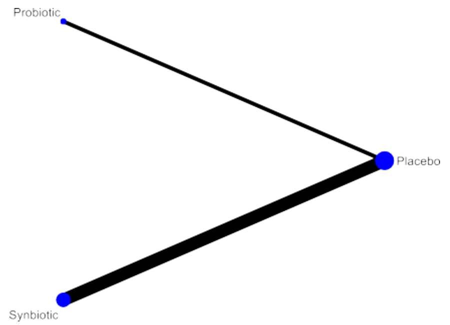

(a)

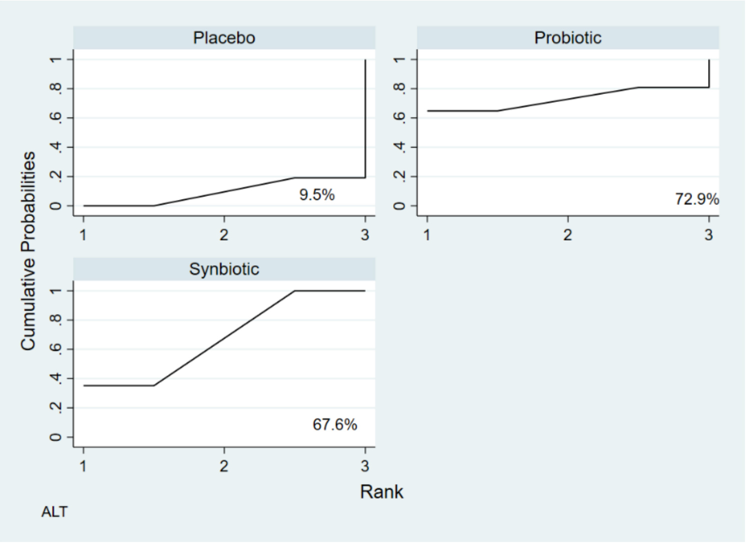

(b)

Figure S39 Sensitivity analysis of the reduction in ALT duration of treatment not less than 12 weeks among NASH patients. (a) network graph and (b) SUCRA curves

Table S28 Network-estimated, weighted mean difference of options for reduction in ALT duration of treatment not less than 12 weeks among NASH patients**.**

Probiotics		
-12.19 (-74.07, 49.68)	Synbiotics	
-28.00 (-89.86, 33.86)	**-15.81 (-17.06, -14.55)**	Placebo

(b)

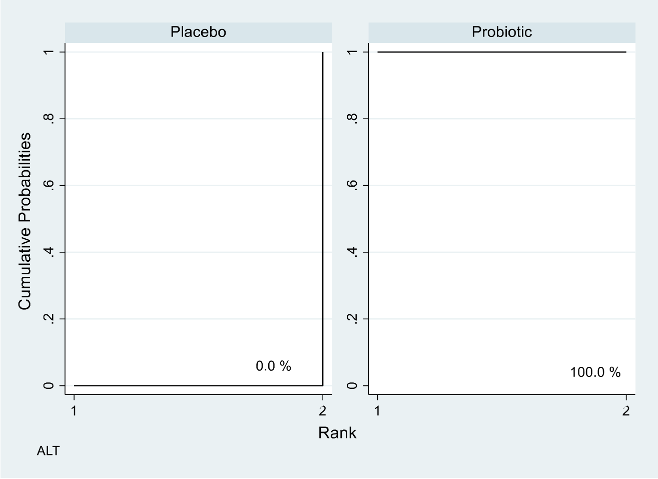

Figure S40 Sensitivity analysis of the reduction in ALT duration of treatment less than 12 weeks among NASH patients. (a) network graph and (b) SUCRA curves

Table S29 Network-estimated, weighted mean difference of options for reduction in ALT duration of treatment less than 12 weeks among NASH patients**.**

Probiotics	
**-34.35 (-45.91, -22.78)**	Placebo

Appendix 15: Sensitivity analyses of BMI outcomes

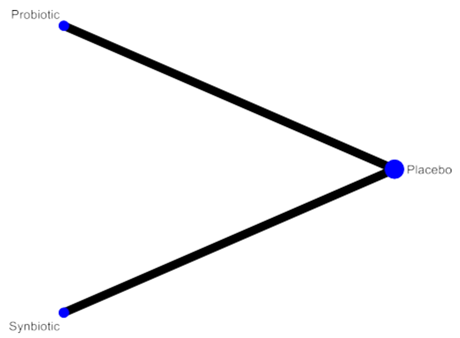

(a)

(b)

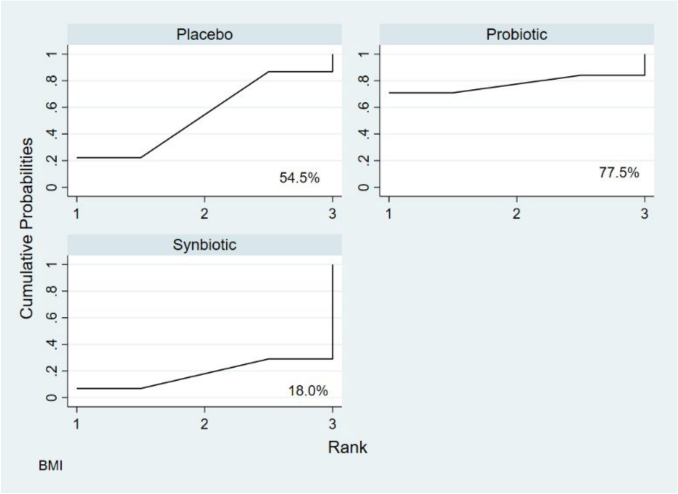

Figure S41 Sensitivity analysis of the reduction in BMI among biopsy-proven NASH patients. (a) network graph and (b) SUCRA curves

Table S30 Network-estimated, weighted mean difference of options for reduction in BMI among biopsy-proven NASH patients**.**

Probiotics		
-0.85 (-2.60, 0.91)	Synbiotics	
-0.50 (-2.08, 1.08)	0.35 (-0.42, 1.11)	Placebo

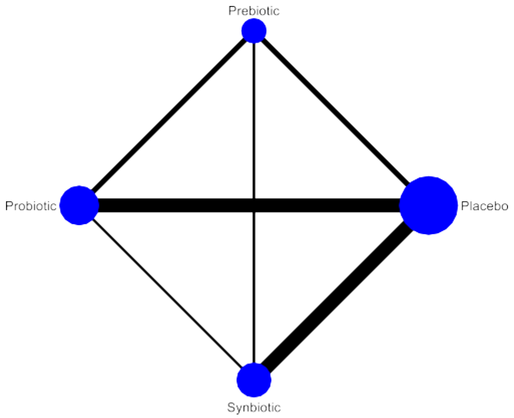

(a)

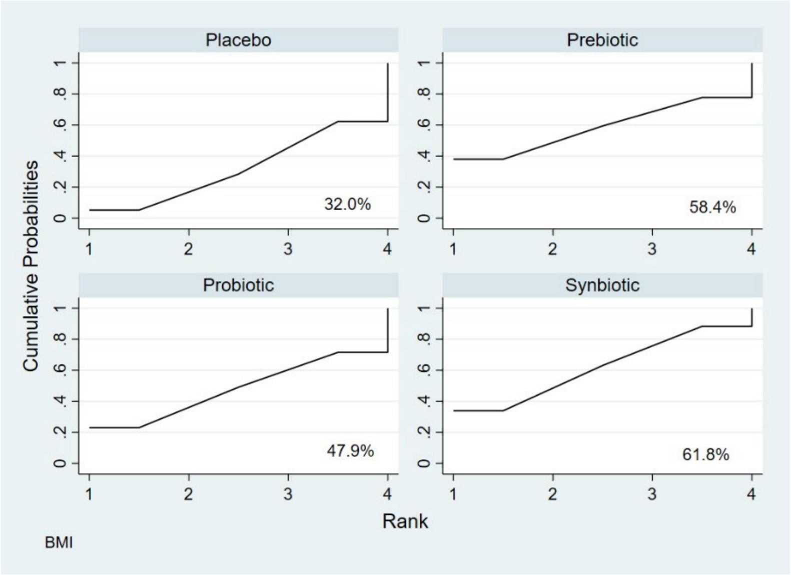

(b)

Figure S42 Sensitivity analysis of the reduction in BMI duration of treatment not less than 12 weeks among NAFLD patients. (a) network graph and (b) SUCRA curves

Table S31 Network-estimated, weighted mean difference of options for reduction in BMI duration of treatment not less than 12 weeks among NAFLD patients**.**

Synbiotics			
0.01 (-1.49, 1.51)	Prebiotics		
-0.13 (-1.46, 1.20)	-0.14 (-1.46, 1.18)	Probiotics	
-0.27 (-0.91, 0.38)	-0.28 (-1.67, 1.11)	-0.14 (-1.34, 1.06)	Placebo

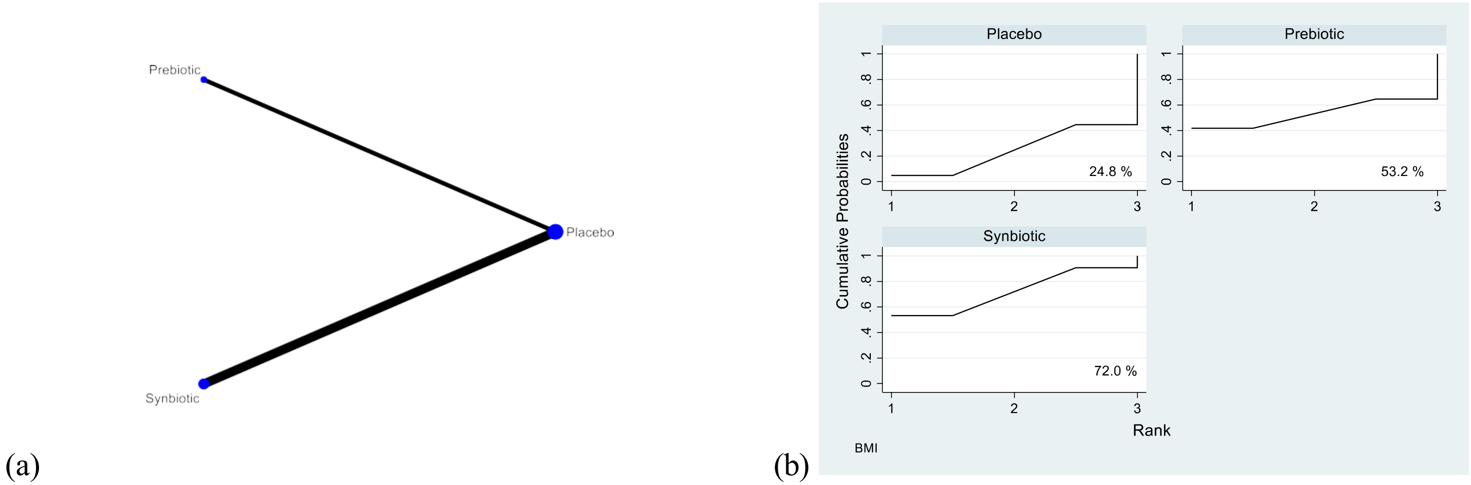

Figure S43 Sensitivity analysis of the reduction in BMI duration of treatment less than 12 weeks among NAFLD patients. (a) network graph and (b) SUCRA curves

Table S32 Network-estimated, weighted mean difference of options for reduction in BMU duration of treatment less than 12 weeks among NAFLD patients**.**

Synbiotics		
-0.16 (-1.97, 1.65)	Prebiotics	
-0.46 (-1.24, 0.33)	-0.30 (-1.93, 1.33)	Placebo

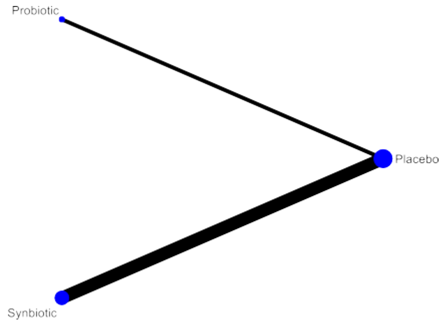

(a)

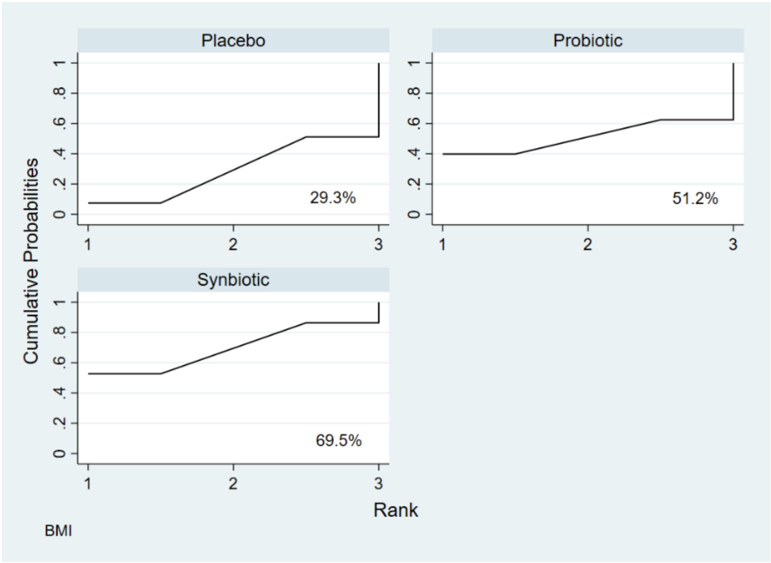

(b)

Figure S44 Sensitivity analysis of the reduction in BMI duration of treatment not less than 12 weeks among NASH patients. (a) network graph and (b) SUCRA curves

Table S33 Network-estimated, weighted mean difference of options for reduction in BMI duration of treatment not less than 12 weeks among NASH patients**.**

Synbiotics		
-0.41 (-4.54, 3.72)	Probiotics	
-0.91 (-2.94, 1.12)	-0.50 (-4.09, 3.09)	Placebo

Appendix 16: Sensitivity analyses of WC outcomes

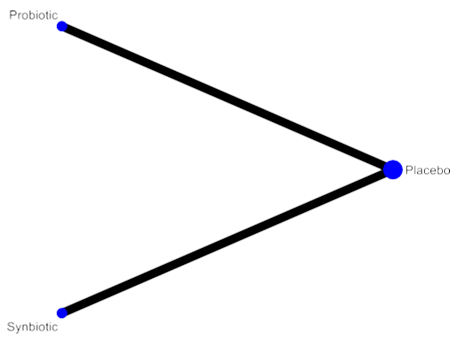

a)

(b)

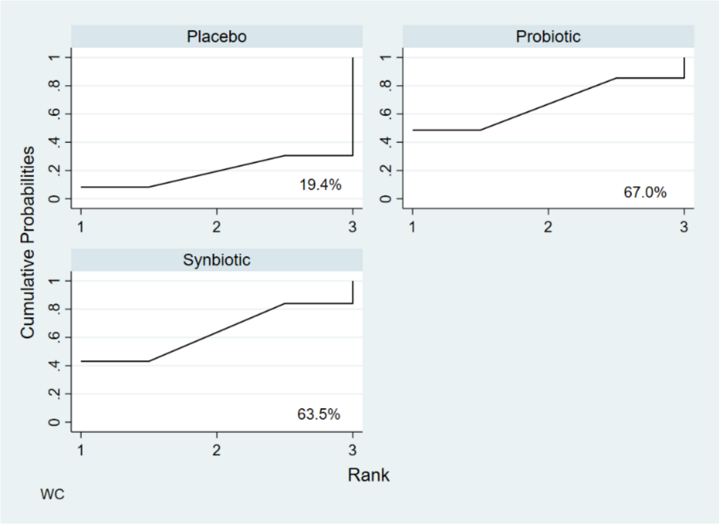

Figure S45 Sensitivity analysis of the reduction in WC among biopsy-proven NASH patients. (a) network graph and (b) SUCRA curves

Table S34 Network-estimated, weighted mean difference of options for reduction in WC among biopsy-proven NASH patients**.**

Probiotics		
-0.20 (-6.90,6.50)	Synbiotics	
-3.00 (-9.81, 3.81)	-2.80 (-9.39, 3.79)	Placebo

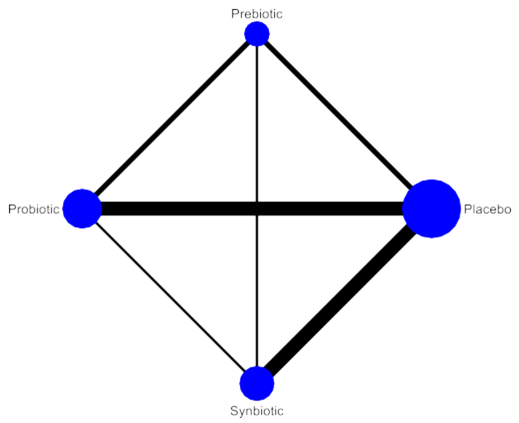

(a)

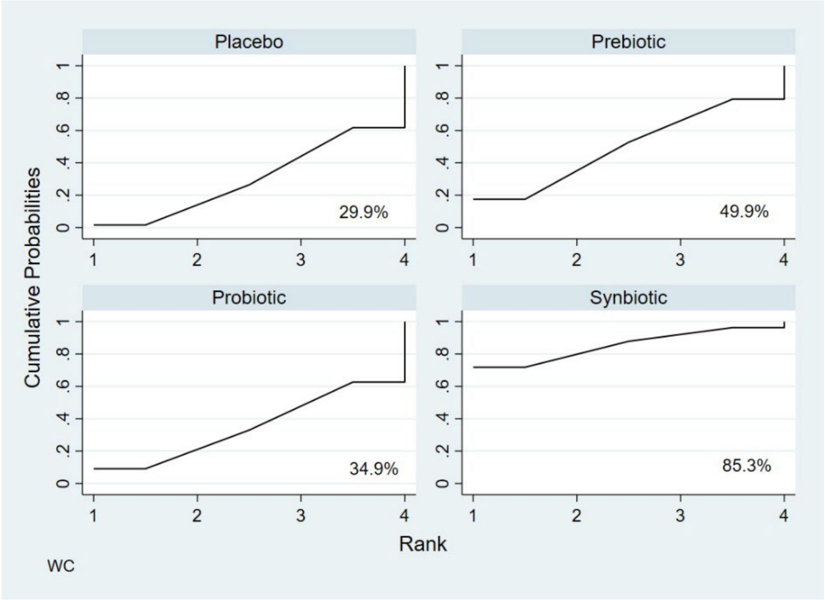

(b)

Figure S46 Sensitivity analysis of the reduction in WC duration of treatment not less than 12 weeks among NAFLD patients. (a) network graph and (b) SUCRA curves

Table S35 Network-estimated, weighted mean difference of options for reduction in WC duration of treatment not less than 12 weeks among NAFLD patients**.**

Synbiotics			
-1.61 (-5.66, 2.44)	Prebiotics		
-2.11 (-6.19, 1.96)	-0.50 (-3.47, 2.47)	Probiotics	
-2.17 (-4.93, 0.60)	-0.56 (-3.47, 2.36)	-0.05 (-2.94, 2.83)	Placebo

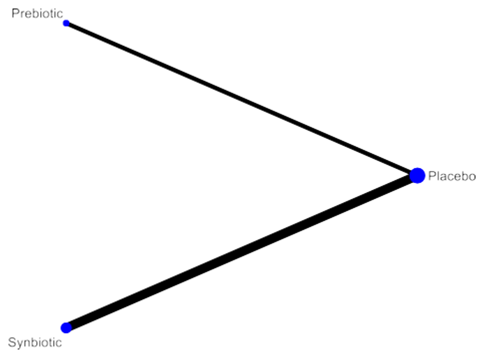

(a)

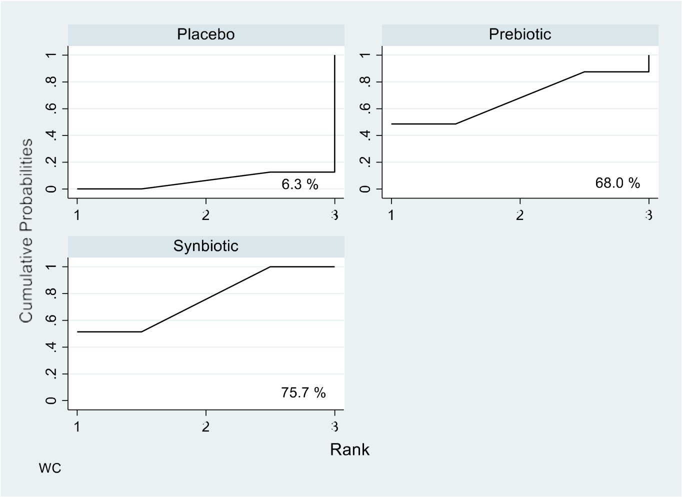

(b)

Figure S47 Sensitivity analysis of the reduction in WC duration of treatment less than 12 weeks among NAFLD patients. (a) network graph and (b) SUCRA curves

Table S36 Network-estimated, weighted mean difference of options for reduction in WC duration of treatment less than 12 weeks among NAFLD patients**.**

Synbiotics		
-0.04 (-3.86, 3.78)	Prebiotics	
**-2.24 (-3.01, -1.48)**	-2.20 (-5.95, 1.55)	Placebo

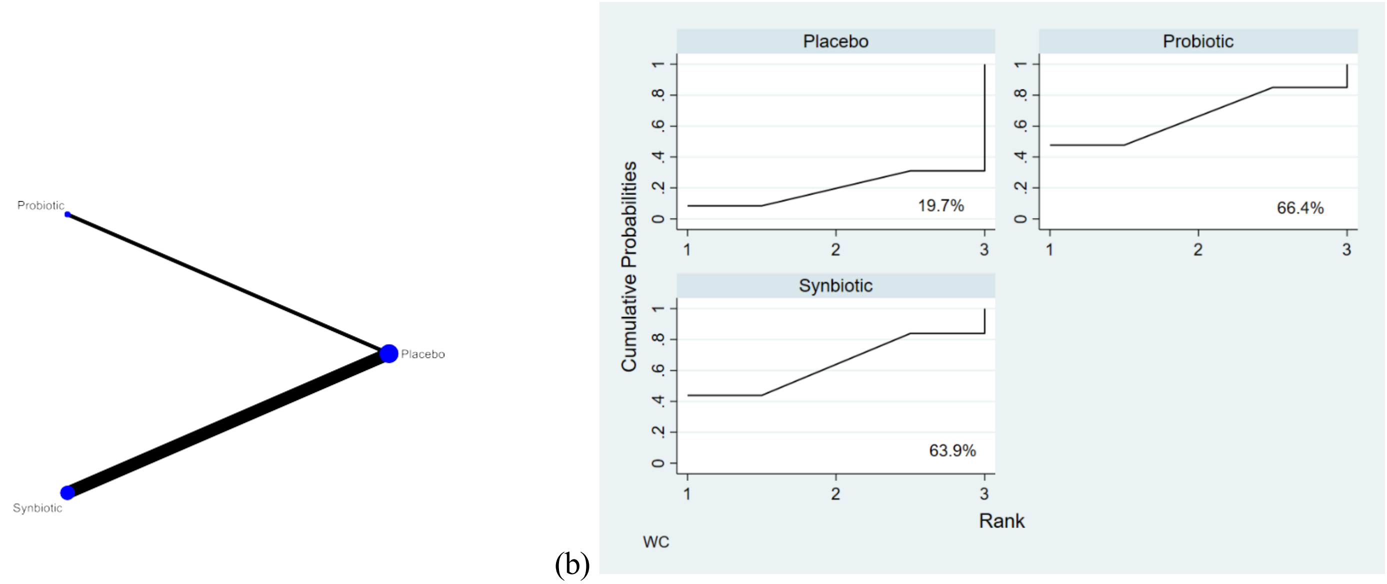

(a) (b)

Figure S48 Sensitivity analysis of the reduction in WC duration of treatment not less than 12 weeks among NASH patients. (a) network graph and (b) SUCRA curves

Table S37 Network-estimated, weighted mean difference of options for reduction in WC duration of treatment not less than 12 weeks among NASH patients**.**

Probiotics		
-0.20 (-6.90, 6.50)	Synbiotics	
-3.00 (-9.81, 3.81)	-2.80 (-9.39, 3.79)	Placebo

Appendix 17: Sensitivity analyses of lipid profile outcomes

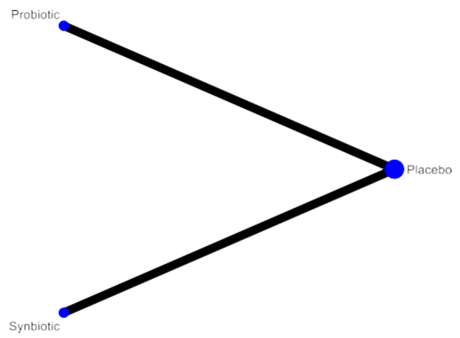

a)

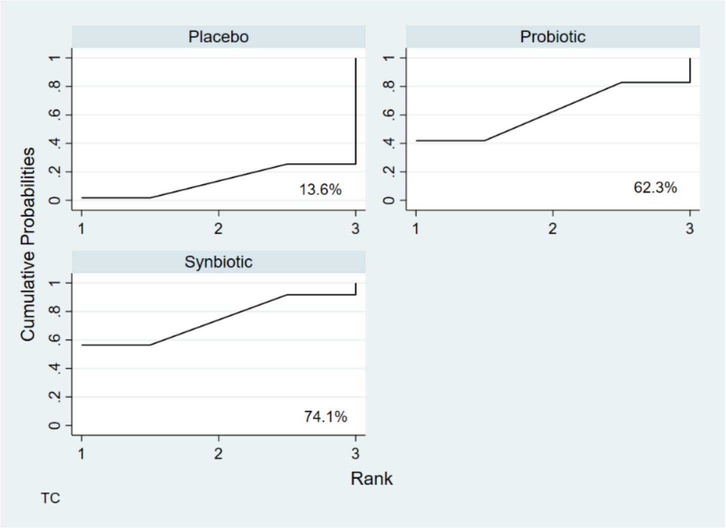

(b)

Figure S49 Sensitivity analysis of the reduction in TC among biopsy-proven NASH patients. (a) network graph and (b) SUCRA curves

Table S38 Network-estimated, weighted mean difference of options for reduction in TC among biopsy-proven NASH patients**.**

Synbiotics		
-1.98 (-23.55, 19.59)	Probiotics	
-9.71 (-23.79, 4.37)	-7.73 (-24.08, 8.62)	Placebo

(a)
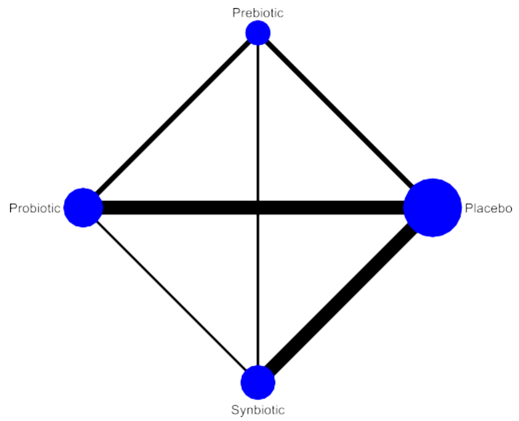

(b)
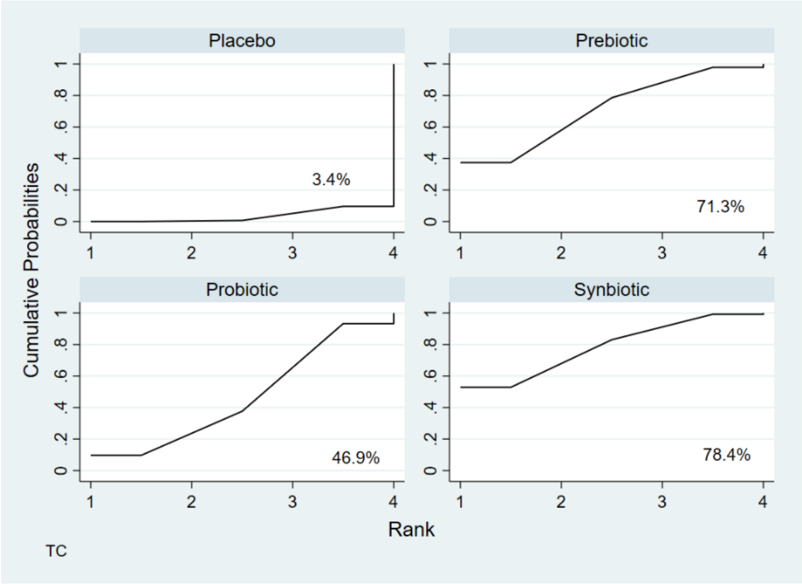

Figure S50 Sensitivity analysis of the reduction in TC duration of treatment not less than 12 weeks among NAFLD patients. (a) network graph and (b) SUCRA curves

Table S39 Network-estimated, weighted mean difference of options for reduction in TC duration of treatment not less than 12 weeks among NAFLD patients**.**

Synbiotics			
-2.01 (-22.22, 18.21)	Prebiotics		
-7.52 (-26.64, 11.60)	-5.51 (-22.58, 11.56)	Probiotics	
**-18.04 (-33.00, -3.09)**	**-16.04 (-32.03, -0.05)**	-10.53 (-24.45, 3.40)	Placebo

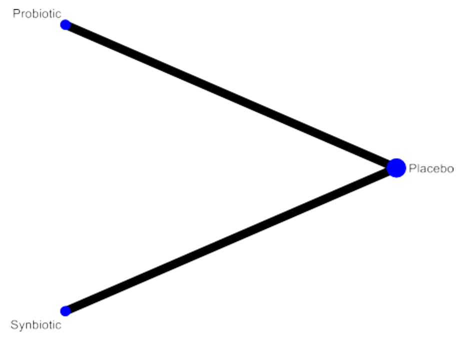

(a)

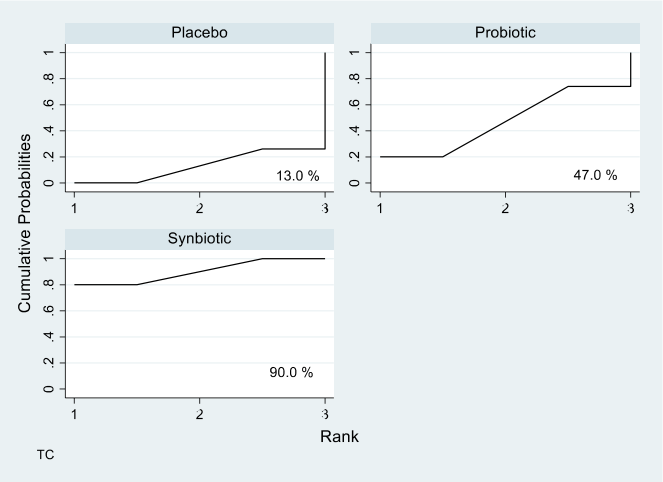

(b)

Figure S51 Sensitivity analysis of the reduction in TC duration of treatment less than 12 weeks among NAFLD patients. (a) network graph and (b) SUCRA curves

Table S40 Network-estimated, weighted mean difference of options for reduction in TC duration of treatment less than 12 weeks among NAFLD patients**.**

Synbiotics		
-14.86 (-48.91, 19.19)	Probiotics	
**-25.67 (-35.75, -15.59)**	-10.81 (-43.33, 21.71)	Placebo

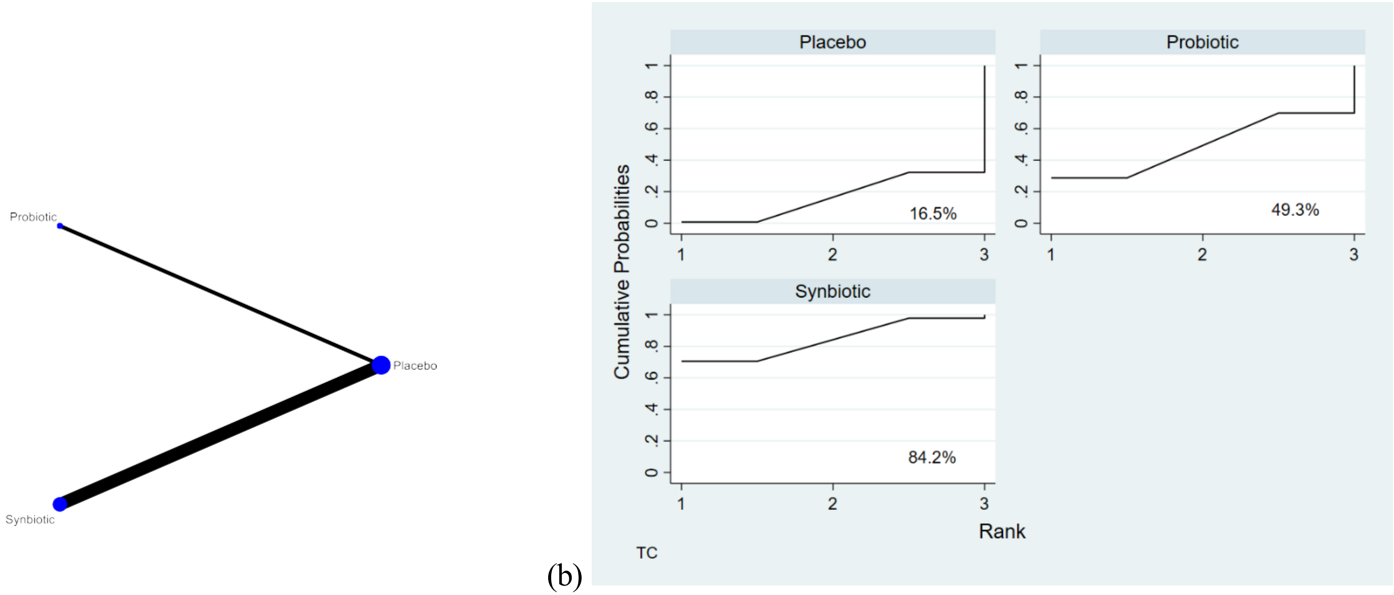

(a) (b)

Figure S52 Sensitivity analysis of the reduction in TC duration of treatment not less than 12 weeks among NASH patients. (a) network graph and (b) SUCRA curves

Table S41 Network-estimated, weighted mean difference of options for reduction in TC duration of treatment not less than 12 weeks among NASH patients**.**

Synbiotics		
-10.35 (-45.65, 24.94)	Probiotics	
**-18.08 (-35.94, -0.23)**	-7.73 (-38.18, 22.72)	Placebo

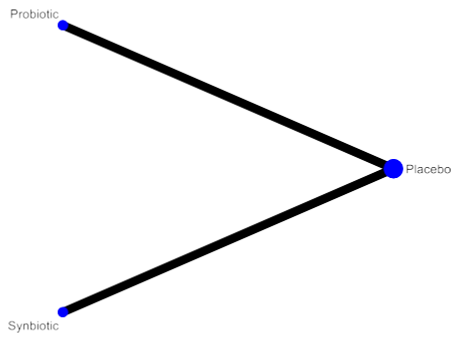

a)

(b)

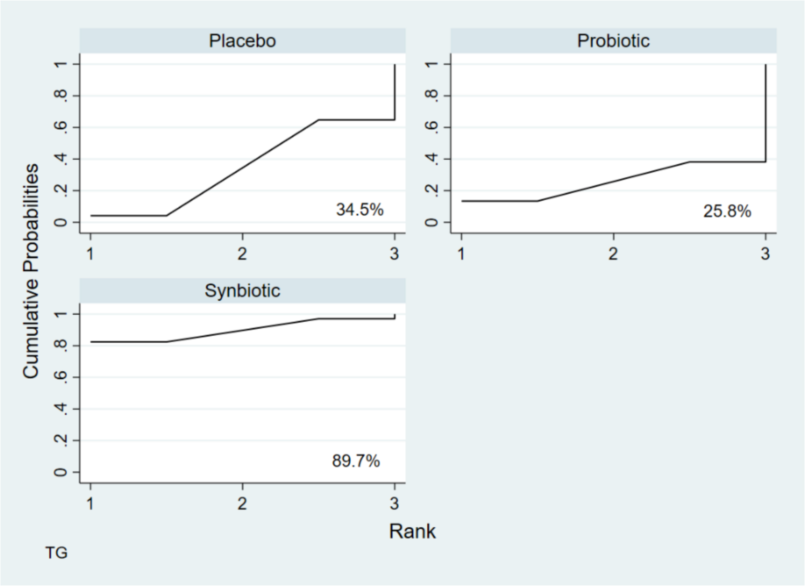

Figure S53 Sensitivity analysis of the reduction in TG among biopsy-proven NASH patients. (a) network graph and (b) SUCRA curves

Table S42 Network-estimated, weighted mean difference of options for reduction in TG among biopsy-proven NASH patients**.**

Synbiotics		
-36.08 (-101.19, 29.03)	Probiotics	
-27.23 (-61.66, 7.20)	8.85 (-46.41, 64.11)	Placebo

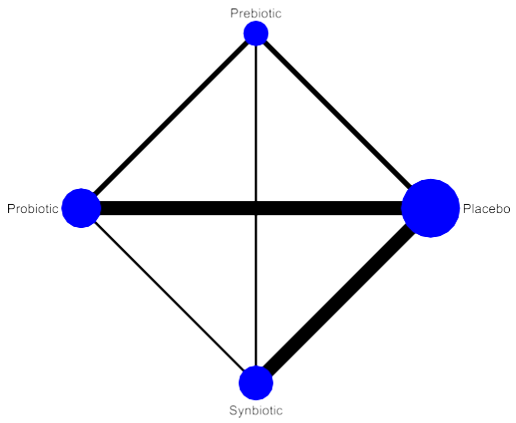

(a)

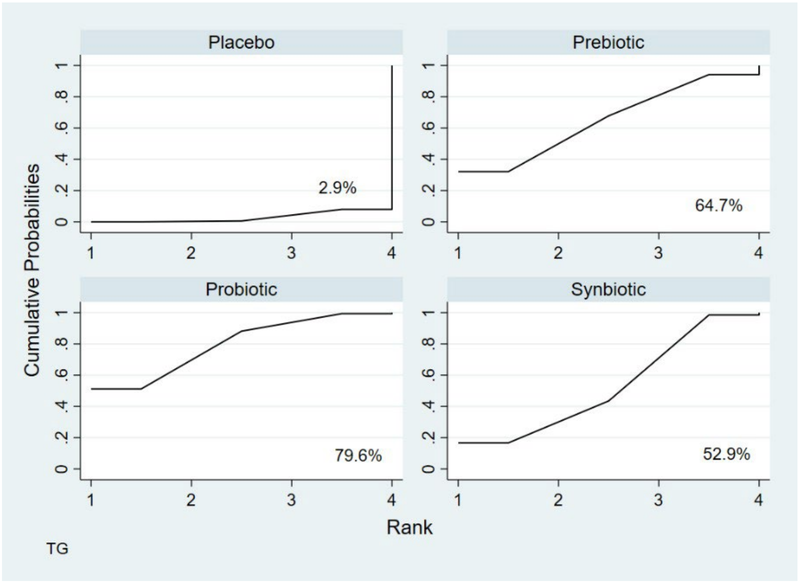

(b)

Figure S54 Sensitivity analysis of the reduction in TG duration of treatment not less than 12 weeks among NAFLD patients. (a) network graph and (b) SUCRA curves

Table S43 Network-estimated, weighted mean difference of options for reduction in TG duration of treatment not less than 12 weeks among NAFLD patients**.**

Probiotics			
-3.98 (-28.34, 20.38)	Prebiotics		
-9.19 (-33.73, 15.35)	-5.21 (-34.66, 24.25)	Synbiotics	
**-25.34 (-46.42, -4.27)**	-21.37 (-48.27, 5.54)	**-16.16 (-31.42, -0.90)**	Placebo

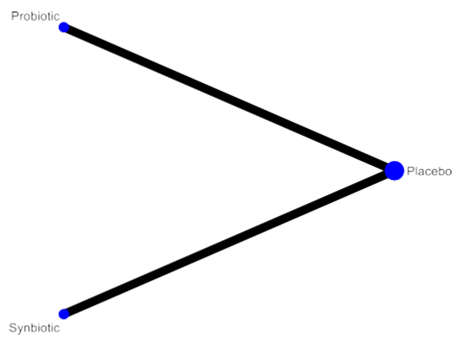

(a)

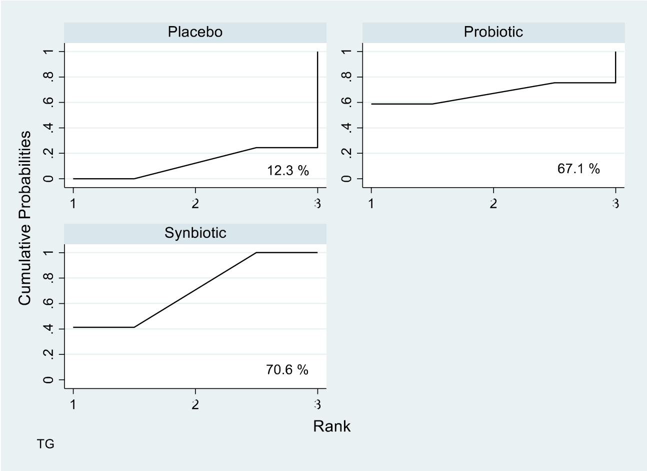

(b)

Figure S55 Sensitivity analysis of the reduction in TG duration of treatment less than 12 weeks among NAFLD patients. (a) network graph and (b) SUCRA curves

Table S44 Network-estimated, weighted mean difference of options for reduction in TG duration of treatment less than 12 weeks among NAFLD patients**.**

Synbiotics		
7.69 (-59.10, 74.47)	Probiotics	
**-15.33 (-22.39, -8.28)**	-23.02 (-89.43, 43.39)	Placebo

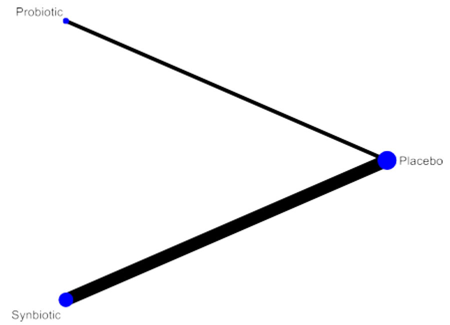

(a)

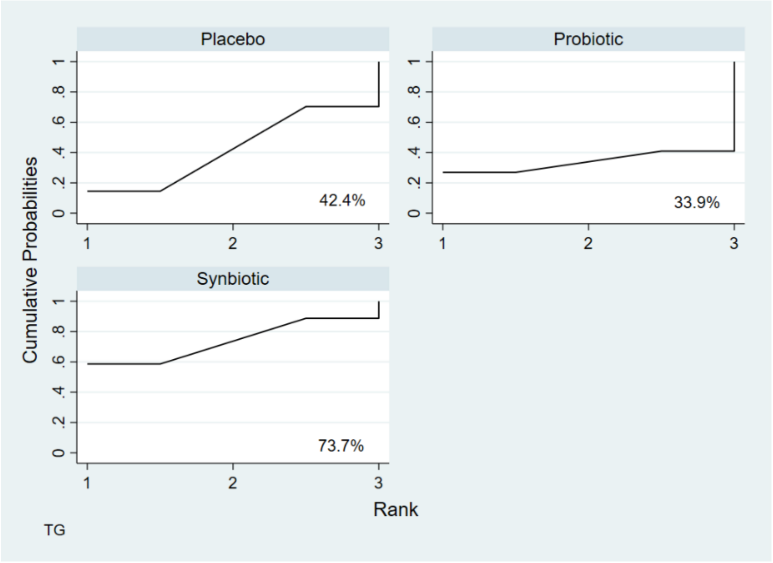

(b)

Figure S56 Sensitivity analysis of the reduction in TG duration of treatment not less than 12 weeks among NASH patients. (a) network graph and (b) SUCRA curves

Table S45 Network-estimated, weighted mean difference of options for reduction in TG duration of treatment not less than 12 weeks among NASH patients**.**

Synbiotics		
-18.28 (-83.23, 46.67)	Probiotics	
-9.43 (-34.97, 16.11)	8.85 (-50.87, 68.57)	Placebo

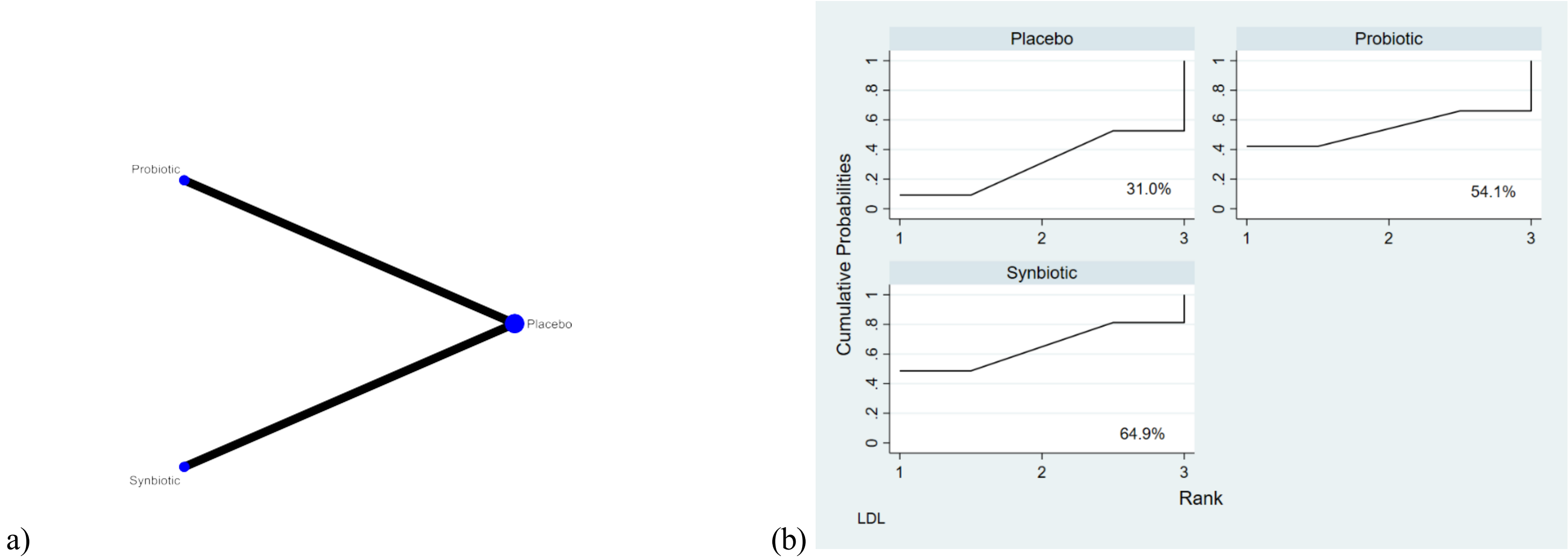

Figure S57 Sensitivity analysis of the reduction in LDL among biopsy-proven NASH patients. (a) network graph and (b) SUCRA curves

Table S46 Network-estimated, weighted mean difference of options for reduction in LDL among biopsy-proven NASH patients.

Synbiotics		
-2.71 (-56.92, 51.51)	Probiotics	
-10.46 (-41.14, 20.23)	-7.75 (-52.44, 36.94)	Placebo

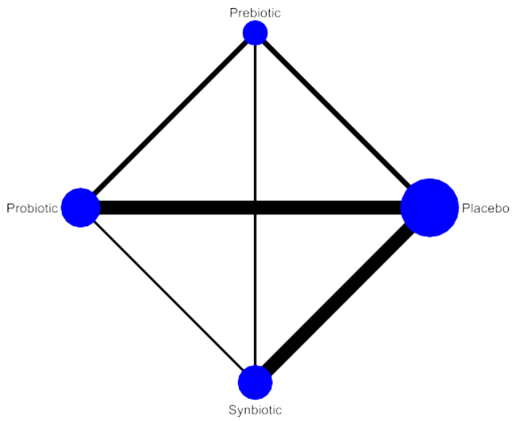

(a)

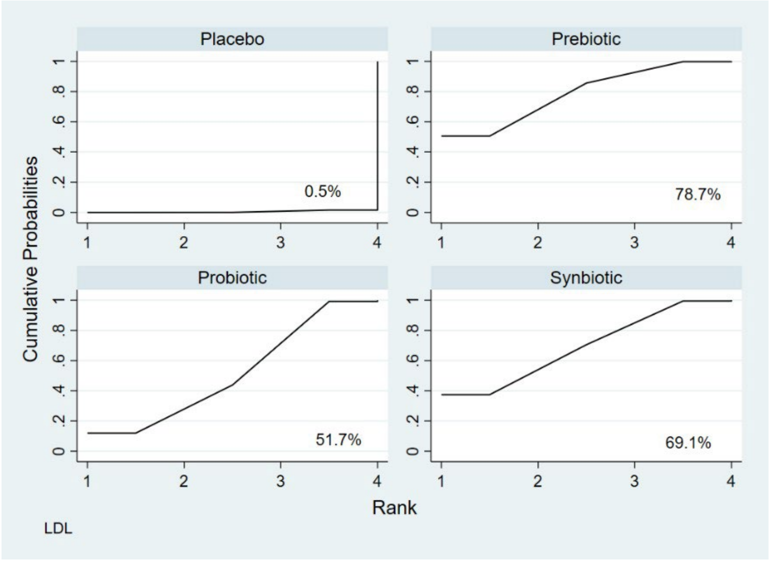

(b)

Figure S58 Sensitivity analysis of the reduction in LDL duration of treatment not less than 12 weeks among NAFLD patients. (a) network graph and (b) SUCRA curves

Table S47 Network-estimated, weighted mean difference of options for reduction in LDL duration of treatment not less than 12 weeks among NAFLD patients**.**

Prebiotics			
-1.55 (-15.77, 12.68)	Synbiotics		
-4.51 (-16.33, 7.30)	-2.97 (-17.05, 11.12)	Probiotics	
**-16.40 (-27.16, -5.63)**	**-14.85 (-26.31, -3.38)**	**-11.88 (-21.69, -2.08)**	Placebo

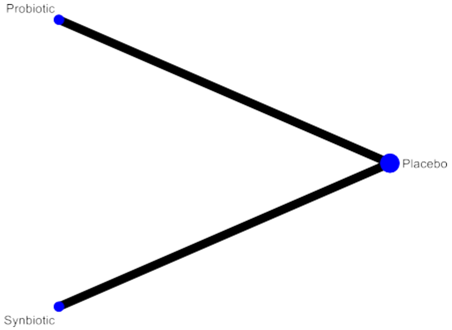

(a)

(b)

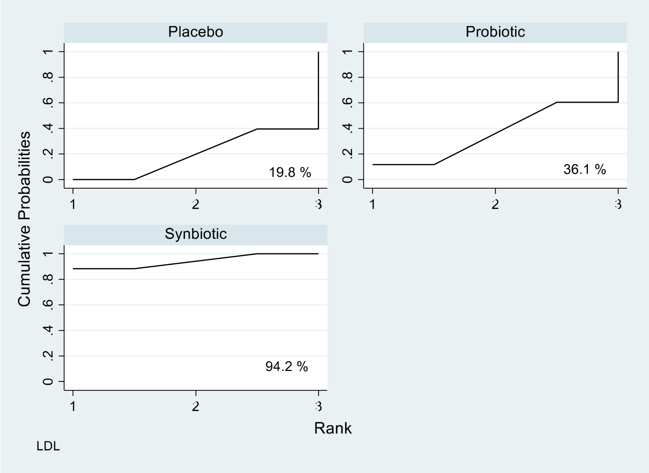

Figure S59 Sensitivity analysis of the reduction in LDL duration of treatment less than 12 weeks among NAFLD patients. (a) network graph and (b) SUCRA curves

Table S48 Network-estimated, weighted mean difference of options for reduction in LDL duration of treatment less than 12 weeks among NAFLD patients**.**

Synbiotics		
-18.38 (-48.09, 11.33)	Probiotics	
**-22.24 (-31.56, -12.91)**	-3.86 (-32.07, 24.35)	Placebo

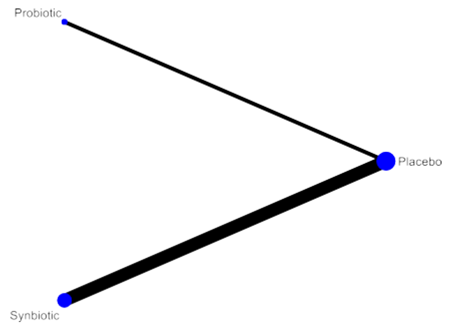

(a)

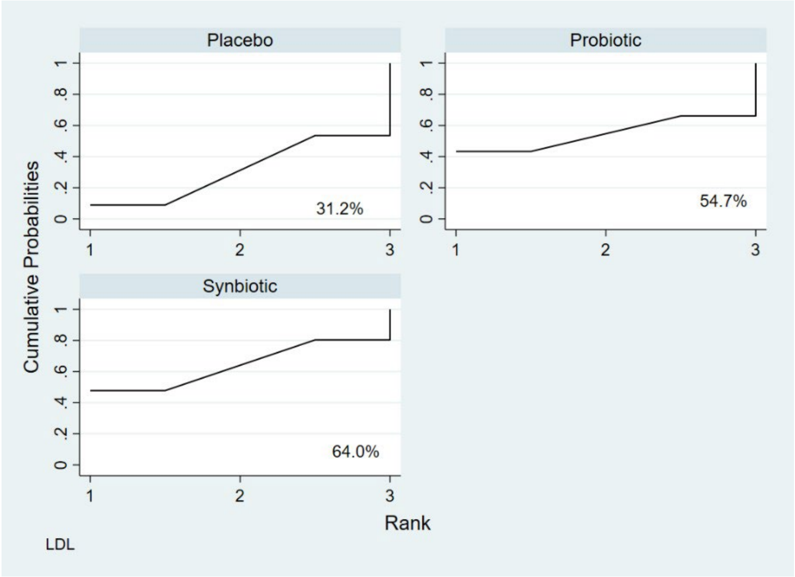

(b)

Figure S60 Sensitivity analysis of the reduction in LDL duration of treatment not less than 12 weeks among NASH patients. (a) network graph and (b) SUCRA curves

Table S49 Network-estimated, weighted mean difference of options for reduction in LDL duration of treatment not less than 12 weeks among NASH patients**.**

Synbiotics		
-2.71 (-56.92, 51.51)	Probiotics	
-10.46 (-41.14, 20.23)	-7.75 (-52.44, 36.94)	Placebo

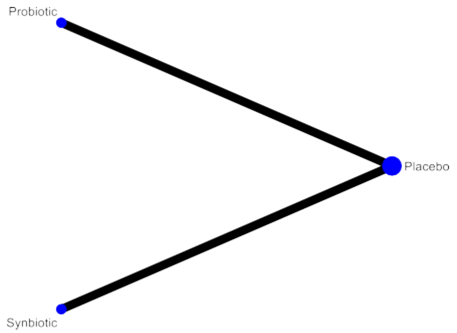

a)

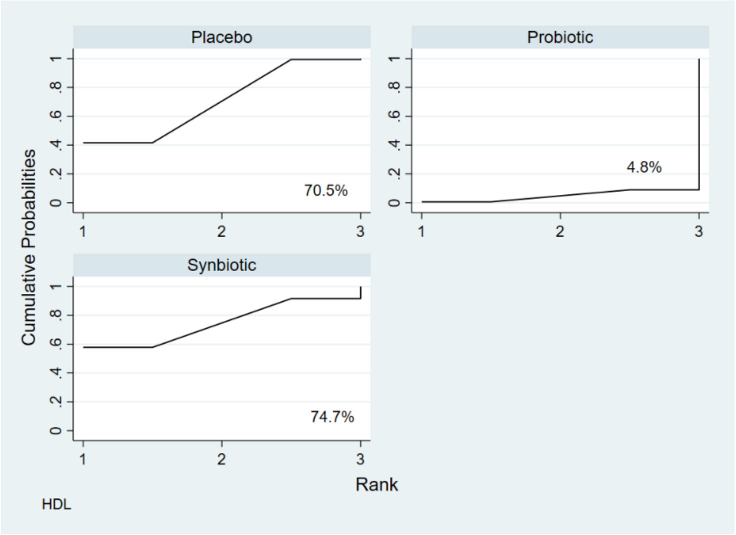

(b)

Figure S61 Sensitivity analysis of the increasing in HDL among biopsy-proven NASH patients. (a) network graph and (b) SUCRA curves

Table S50 Network-estimated, weighted mean difference of options for increasing in HDL among biopsy-proven NASH patients**.**

Synbiotics		
4.42 (-1.94, 10.78)	Probiotics	
0.56 (-4.81, 5.94)	**<u>-3.86 (-7.25, -0.47)</u>**	Placebo

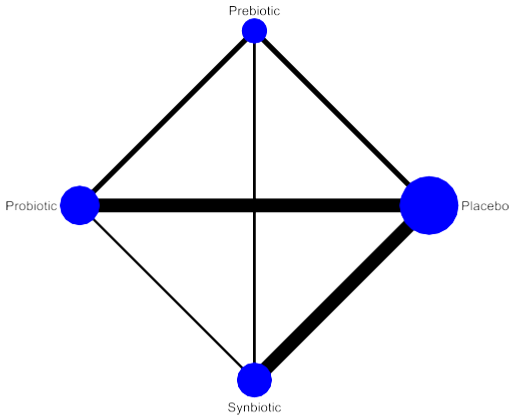

(a)

(b)

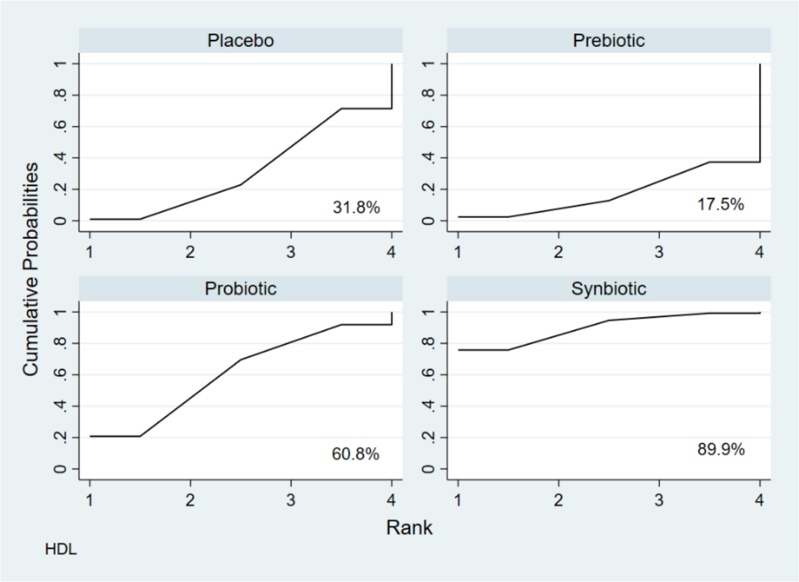

Figure S62 Sensitivity analysis of the increasing in HDL duration of treatment not less than 12 weeks among NAFLD patients. (a) network graph and (b) SUCRA curves

Table S51 Network-estimated, weighted mean difference of options for increasing in HDL duration of treatment not less than 12 weeks among NAFLD patients.

Synbiotics			
1.45 (-2.24, 5.14)	Probiotics		
3.22 (-0.72, 7.15)	1.77 (-1.51, 5.04)	Prebiotics	
2.48 (-0.05, 5.01)	1.03 (-2.00, 4.05)	-0.74 (-4.05, 2.57)	Placebo

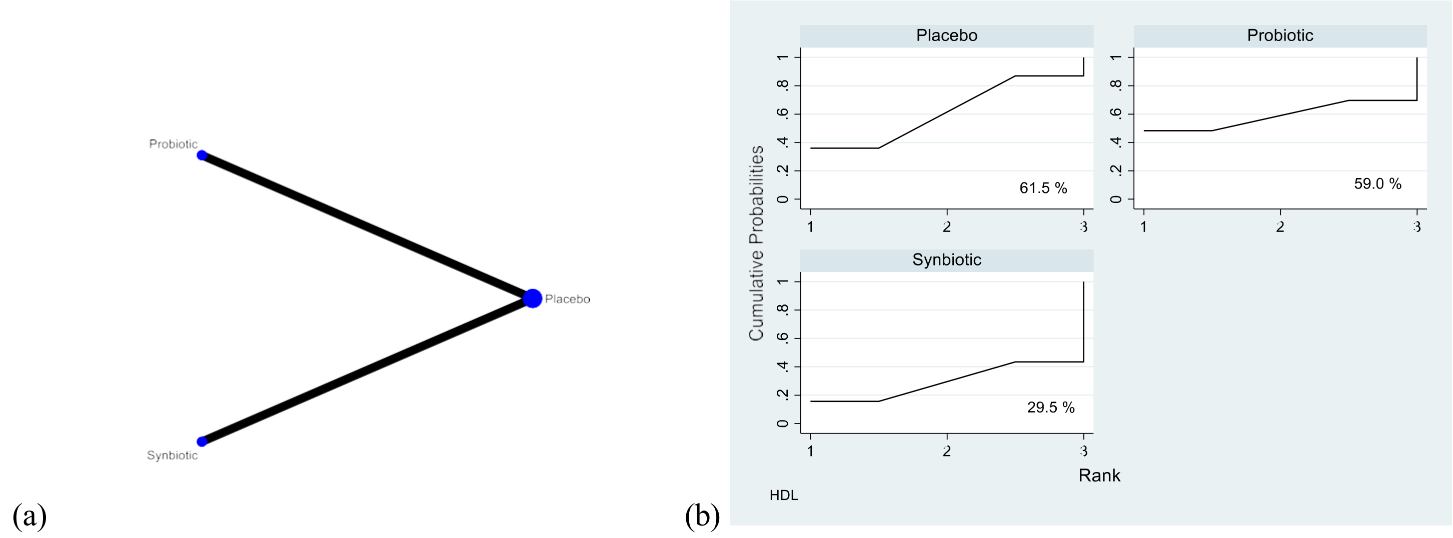

Figure S63 Sensitivity analysis of the increasing in HDL duration of treatment less than 12 weeks among NAFLD patients. (a) network graph and (b) SUCRA curves

Table S52 Network-estimated, weighted mean difference of options for increasing in HDL duration of treatment less than 12 weeks among NAFLD patients**.**

Synbiotics		
-3.81 (-22.19, 14.56)	Probiotics	
-3.43 (-13.26, 6.40)	0.38 (-15.14, 15.90)	Placebo

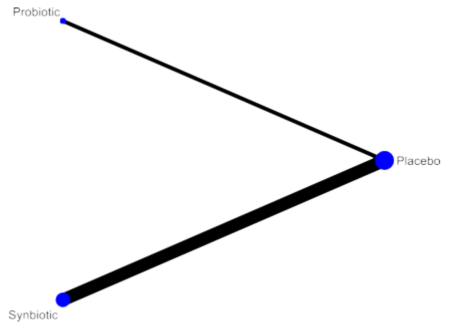

(a)

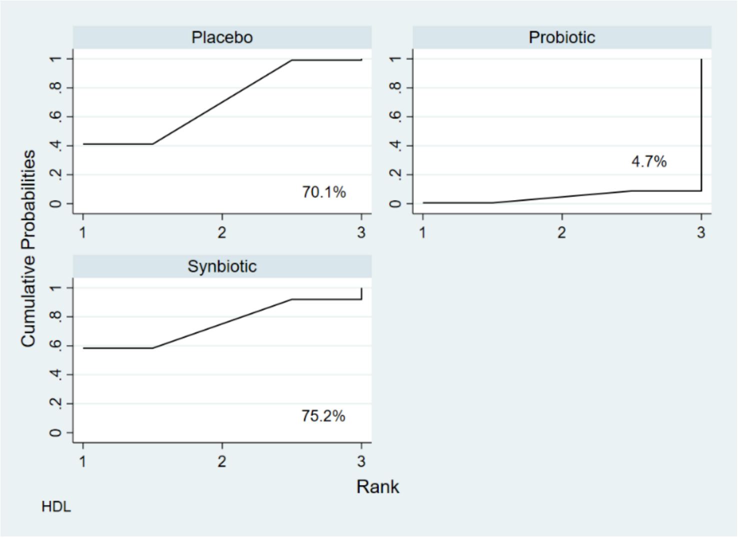

(b)

Figure S64 Sensitivity analysis of the increasing in HDL duration of treatment not less than 12 weeks among NASH patients. (a) network graph and (b) SUCRA curves

Table S53 Network-estimated, weighted mean difference of options for increasing in HDL duration of treatment not less than 12 weeks among NASH patients**.**

Synbiotics		
4.42 (-1.94, 10.78)	Probiotics	
0.56 (-4.81, 5.94)	**-3.86 (-7.25, -0.47)**	Placebo

Appendix 18: Sensitivity analyses of FBS outcome

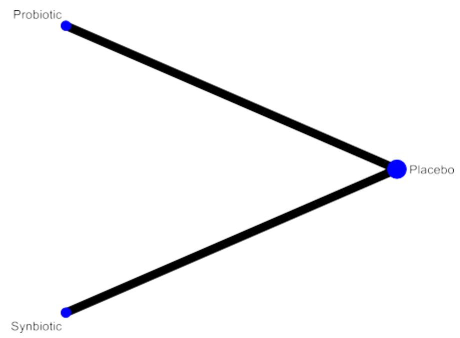

a)

(b)

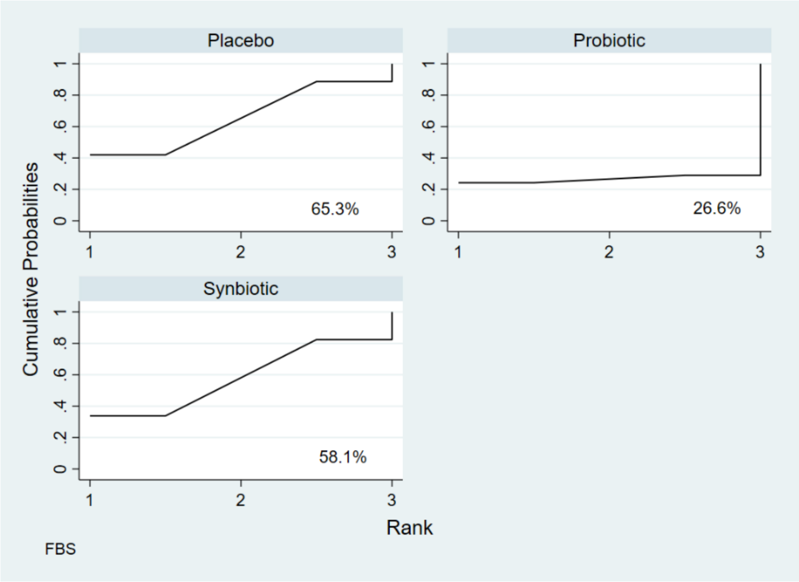

Figure S65 Sensitivity analysis of the reduction in FBS among biopsy-proven NASH patients. (a) network graph and (b) SUCRA curves

Table S54 Network-estimated, weighted mean difference of options for reduction in FBS among biopsy-proven NASH patients**.**

Placebo		
-0.46 (-6.20, 5.28)	Synbiotics	
-10.80 (-44.08, 22.48)	-10.34 (-44.12, 23.43)	Probiotics

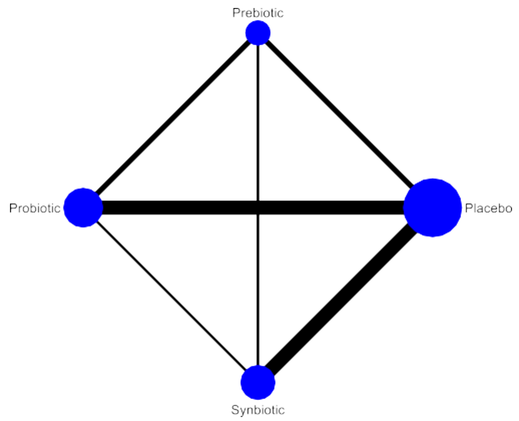

(a)

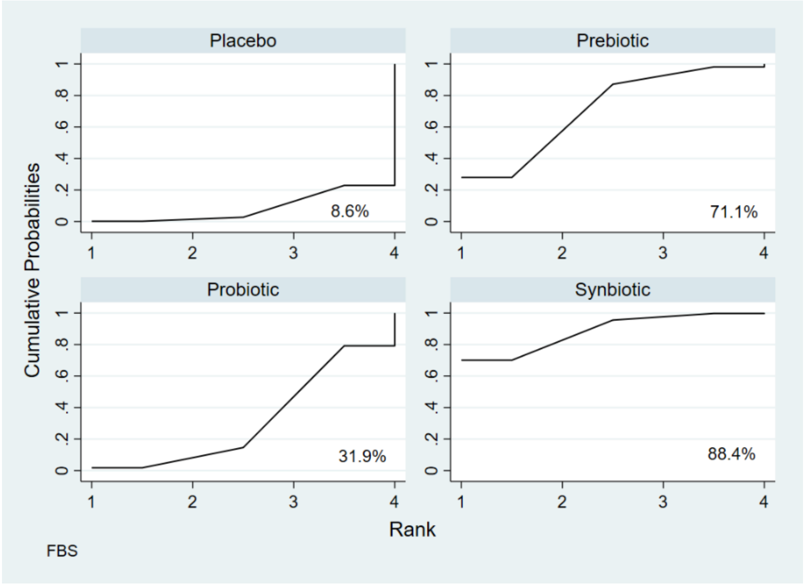

(b)

Figure S66 Sensitivity analysis of the reduction in FBS duration of treatment not less than 12 weeks among NAFLD patients. (a) network graph and (b) SUCRA curves

Table S55 Network-estimated, weighted mean difference of options for reduction in FBS duration of treatment not less than 12 weeks among NAFLD patients**.**

Synbiotics			
-1.34 (-6.00, 3.32)	Prebiotics		
-3.68 (-8.09, 0.74)	-2.34 (-6.24, 1.57)	Probiotics	
**-5.15 (-9.18, -1.12)**	-3.81 (-8.00, 0.38)	-1.47 (-5.18, 2.23)	Placebo

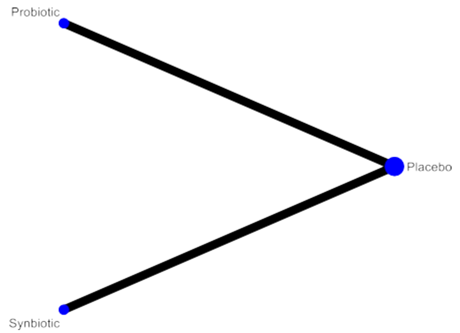

(a)

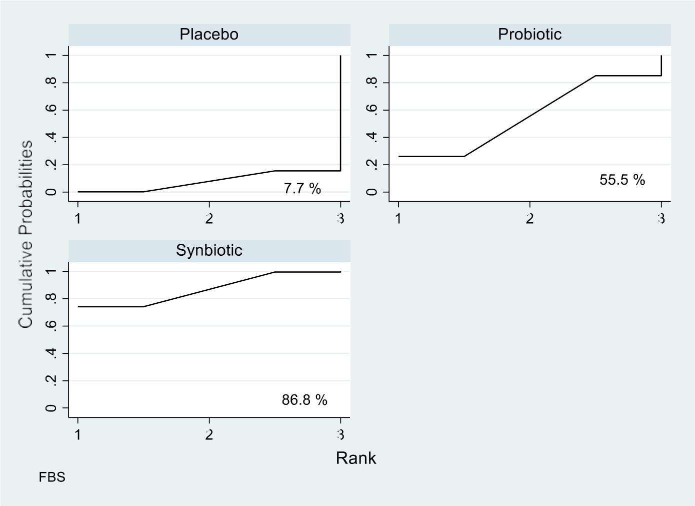

(b)

Figure S67 Sensitivity analysis of the reduction in FBS duration of treatment less than 12 weeks among NAFLD patients. (a) network graph and (b) SUCRA curves

Table S56 Network-estimated, weighted mean difference of options for reduction in FBS duration of treatment less than 12 weeks among NAFLD patients**.**

Synbiotics		
-5.45 (-21.88, 10.99)	Probiotics	
**-12.60 (-22.28, -2.92)**	-7.15 (-20.44, 6.14)	Placebo

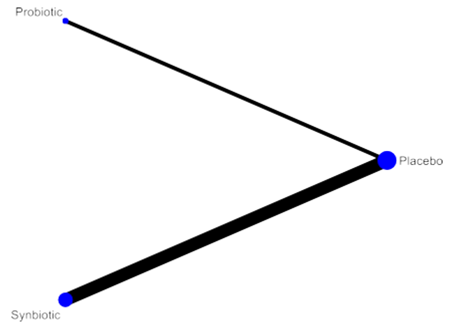

(a)

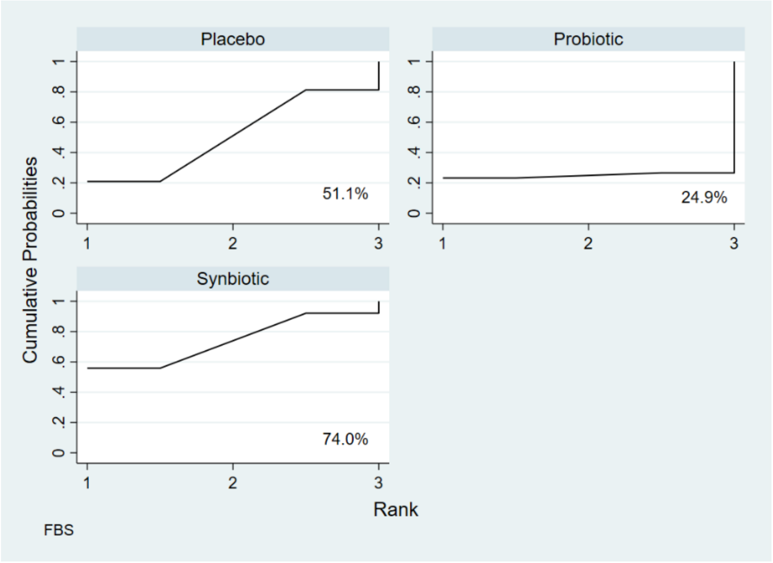

(b)

Figure S68 Sensitivity analysis of the reduction in FBS duration of treatment not less than 12 weeks among NASH patients. (a) network graph and (b) SUCRA curves

Table S57 Network-estimated, weighted mean difference of options for reduction in FBS duration of treatment not less than 12 weeks among NASH patients**.**

Synbiotics		
-11.92 (-45.42, 21.57)	Probiotics	
-1.12 (-4.87, 2.63)	10.80 (-22.48, 44.08)	Placebo

Appendix 19: Sensitivity analyses of HOMA-IR outcomes

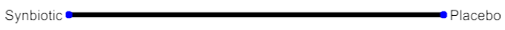

a)

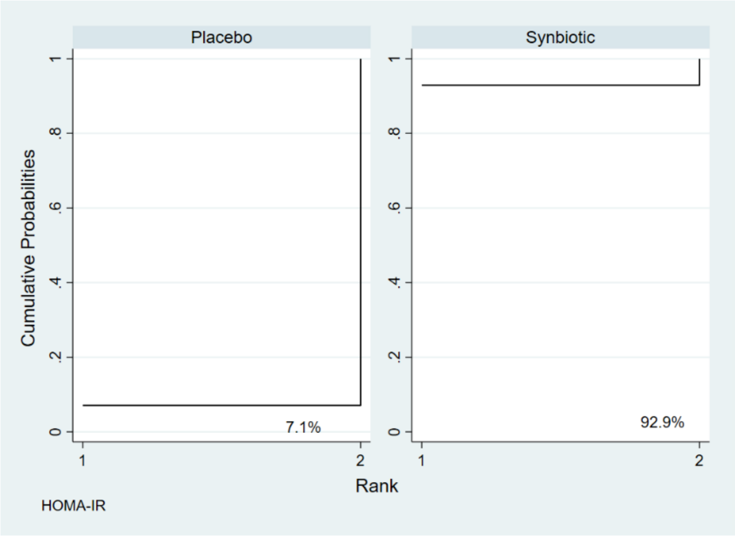

(b)

Figure S69 Sensitivity analysis of the reduction in HOMA-IR among biopsy-proven NASH patients. (a) network graph and (b) SUCRA curves

Table S58 Network-estimated, weighted mean difference of options for reduction in HOMA-IR among biopsy-proven NASH patients**.**

Synbiotics	
-1.70 (-3.99, 0.59)	Placebo

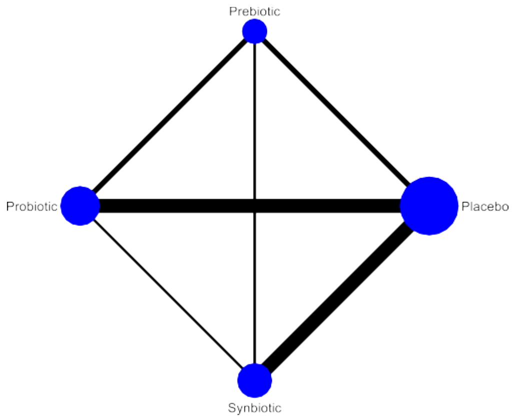

(a)

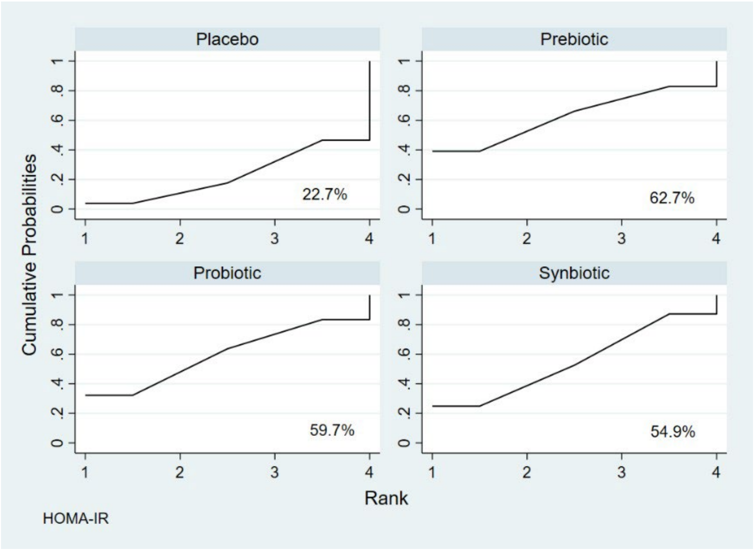

(b)

Figure S70 Sensitivity analysis of the reduction in HOMA-IR duration of treatment not less than 12 weeks among NAFLD patients. (a) network graph and (b) SUCRA curves

Table S59 Network-estimated, weighted mean difference of options for reduction in HOMA-IR duration of treatment not less than 12 weeks among NAFLD patients**.**

Prebiotics			
-0.05 (-1.21, 1.11)	Probiotics		
-0.14 (-1.37, 1.08)	-0.10 (-1.27, 1.08)	Synbiotics	
-0.44 (-1.62, 0.74)	-0.39 (-1.51, 0.73)	-0.30 (-1.01, 0.41)	Placebo

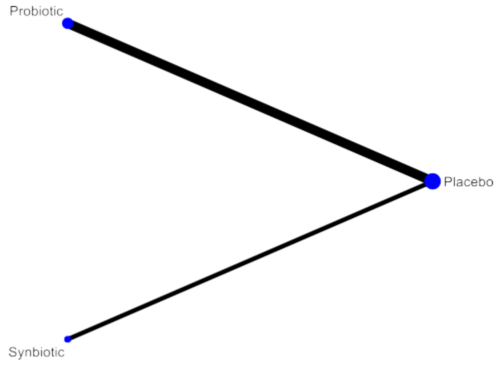

(a)

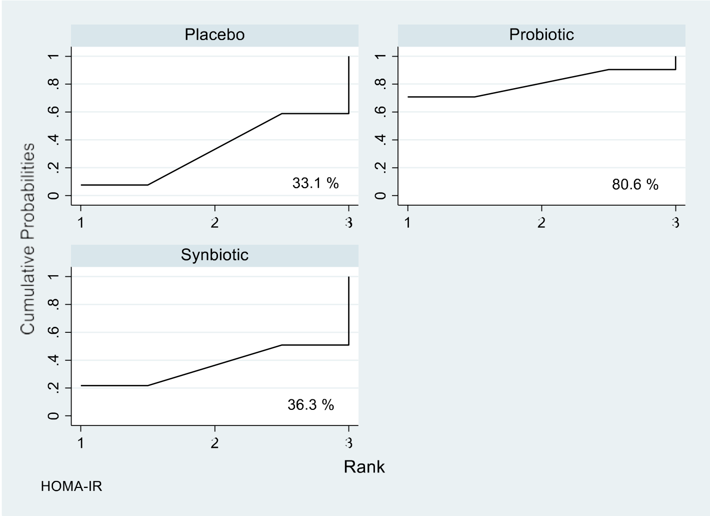

(b)

Figure S71 Sensitivity analysis of the reduction in HOMA-IR duration of treatment less than 12 weeks among NAFLD patients. (a) network graph and (b) SUCRA curves

Table S60 Network-estimated, weighted mean difference of options for reduction in HOMA-IR duration of treatment less than 12 weeks among NAFLD patients**.**

Probiotics		
-0.60 (-2.29, 1.08)	Synbiotics	
-0.58 (-1.68, 0.51)	0.02 (-1.26, 1.30)	Placebo

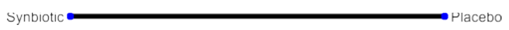

(a)

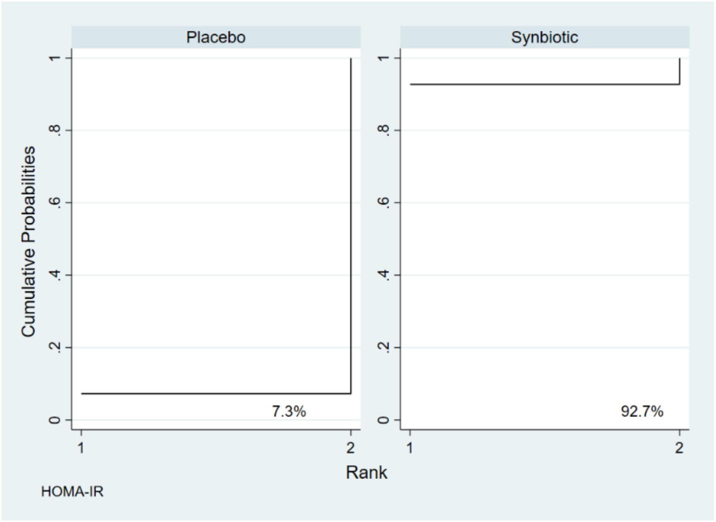

(b)

Figure S72 Sensitivity analysis of the reduction in HOMA-IR duration of treatment not less than 12 weeks among NASH patients. (a) network graph and (b) SUCRA curves

Table S61 Network-estimated, weighted mean difference of options for reduction in HOMA-IR duration of treatment not less than 12 weeks among NASH patients**.**

Synbiotics	
-1.70 (-3.99, 0.59)	Placebo
